# Global occurrence of the bacteria with capability for extracellular reduction of iodate

**DOI:** 10.3389/fmicb.2022.1070601

**Published:** 2022-11-25

**Authors:** Jinzhi Guo, Jie Jiang, Zhaofeng Peng, Yuhong Zhong, Yongguang Jiang, Zhou Jiang, Yidan Hu, Yiran Dong, Liang Shi

**Affiliations:** ^1^Department of Biological Sciences and Technology, School of Environmental Studies, China University of Geosciences, Wuhan, China; ^2^State Key Laboratory of Biogeology and Environmental Geology, China University of Geosciences, Wuhan, China; ^3^Hubei Key Laboratory of Yangtze Catchment Environmental Aquatic Science, China University of Geosciences, Wuhan, China; ^4^State Environmental Protection Key Laboratory of Source Apportionment and Control of Aquatic Pollution, Ministry of Ecology and Environment, China University of Geosciences, Wuhan, China

**Keywords:** DmsEFAB, MtrCAB, extracellular reduction of iodate, *Ferrimonas*, *Shewanella*, global biogeochemical cycling of iodine

## Abstract

The γ-proteobacterium *Shewanella oneidensis* MR-1 reduces iodate to iodide extracellularly. Both *dmsEFAB* and *mtrCAB* gene clusters are involved in extracellular reduction of iodate by *S. oneidensis* MR-1. DmsEFAB reduces iodate to hypoiodous acid and hydrogen peroxide (H_2_O_2_). Subsequently, H_2_O_2_ is reduced by MtrCAB to facilitate DmsEFAB-mediated extracellular reduction of iodate. To investigate the distribution of bacteria with the capability for extracellular reduction of iodate, bacterial genomes were systematically searched for both *dmsEFAB* and *mtrCAB* gene clusters. The *dmsEFAB* and *mtrCAB* gene clusters were found in three *Ferrimonas* and 26 *Shewanella* species. Coexistence of both *dmsEFAB* and *mtrCAB* gene clusters in these bacteria suggests their potentials for extracellular reduction of iodate. Further analyses demonstrated that these bacteria were isolated from a variety of ecosystems, including the lakes, rivers, and subsurface rocks in East and Southeast Asia, North Africa, and North America. Importantly, most of the bacteria with both *dmsEFAB* and *mtrCAB* gene clusters were found in different marine environments, which ranged from the Arctic Ocean to Antarctic coastal marine environments as well as from the Atlantic Ocean to the Indian and Pacific Oceans. Widespread distribution of the bacteria with capability for extracellular reduction of iodate around the world suggests their significant importance in global biogeochemical cycling of iodine. The genetic organization of *dmsEFAB* and *mtrCAB* gene clusters also varied substantially. The identified *mtrCAB* gene clusters often contained additional genes for multiheme *c*-type cytochromes. The numbers of *dmsEFAB* gene cluster detected in a given bacterial genome ranged from one to six. In latter, duplications of *dmsEFAB* gene clusters occurred. These results suggest different paths for these bacteria to acquire their capability for extracellular reduction of iodate.

## Introduction

Iodine (I) is a trace element of both health and environmental significance. As iodine is a crucial component of human thyroid hormones triiodothyronine and thyroxine, iodine deficiency disorders of humans (e.g., goiter and cretinism) are attributed to insufficient intake of iodine, such as drinking of the groundwater with low iodine level (Laurberg et al., 2001; Zimmermann, 2009; Zhang E. et al., 2013; Duan et al., 2016). Excessive intake of iodine, such as drinking of the groundwater with high iodine level, also results in thyroiditis and probably cancer (Laurberg et al., 2001; Andersen et al., 2009; Zhang E. et al., 2013; Duan et al., 2016). Thus, abnormal level of iodine in groundwater affects human health (Wen et al., 2013; Zhang E. et al., 2013; Duan et al., 2016). Furthermore, radioactive iodine-129 (^129^I) is an important risk driver of the Hanford and Savannah River Sites in Washington and South Carolina States, respectively, United States, where ^129^I level in groundwater is higher than that for the drinking water standard (Otosaka et al., 2011; Zhang S. et al., 2013; Kaplan et al., 2014). Finally, global cycling of iodine impacts air quality and climate (Carpenter et al., 2021).

In environment, iodate (IO_3_^−^) and iodide (I^−^) are the two dominant species of inorganic iodine. For example, IO_3_^−^ is the major iodine species found in groundwater of the Hanford and Savannah River Sites (Otosaka et al., 2011; Zhang S. et al., 2013), while the dominant species found in the groundwater in Datong and Taiyuan Basins, China and North China Plains is I^−^ (Li et al., 2013; Tang et al., 2013; Zhang E. et al., 2013). Both IO_3_^−^and I^−^ are found in oceans where their combined concentration is 0.4–0.5 μM (Chance et al., 2014).

Microorganisms play crucial roles in redox transformation of IO_3_^−^and I^−^ in environments. I^−^-oxidizing microorganisms oxidize I^−^ to molecular iodine (I_2_), while IO_3_^−^-reducing microorganisms reduce IO_3_^−^ to I^−^ (Iino et al., 2016; Yamazaki et al., 2020; Reyes-Umana et al., 2022). The enzymes involved in microbial oxidation of I^−^ to I_2_ include the extracellular multicopper oxidase LoxA (Suzuki et al., 2012; Shiroyama et al., 2015), while those involved in microbial reduction of IO_3_^−^ to I^−^ include two different types of enzymes (Yamazaki et al., 2020; Guo et al., 2022; Reyes-Umana et al., 2022; Shin et al., 2022).

IdrABP_1_P_2_ of the dissimilatory IO_3_^−^-reducing bacterium *Pseudomonas* sp. strain SCT is the first enzyme identified for IO_3_^−^ reduction (Yamazaki et al., 2020). This enzyme consists of four subunits, in which IdrA is suggested to be the catalytic subunit, IdrB is the electron transfer subunit and IdrP_1_ and IdrP_2_ are the detoxification subunits. All of these subunits are believed to be localized in the periplasm. During IO_3_^−^ reduction, IdrB is proposed to receive electrons from cytochrome *c* (Cyt-*c*) in the periplasm and then transfers the electrons to IdrA. IdrA is suggested to use the electrons to reduce IO_3_^−^ to hypoiodous acid (HIO) and hydrogen peroxide (H_2_O_2_). The generated H_2_O_2_ is proposed to be reduced to H_2_O by IdrP_1_ and IdrP_2_. Cyt-*c* may also supply electrons to IdrP_1_ and IdrP_2_. HIO is suggested to be further reduced to I^−^ probably by Cld (Yamazaki et al., 2020). Based on their polypeptide sequences, IdrA and IdrB are the homologs of dimethylsulfoxide (DMSO) reductase DmsA and DmsB, respectively. IdrP_1_ and IdrP_2_ are the *c*-type cytochromes (*c*-Cyt) that are peroxidases. The genes for IdrABP_1_P_2_ are clustered together in the genome of *Pseudomonas* sp. strain SCT (Yamazaki et al., 2020).

The *idrABP_1_P_2_* gene cluster also exists in the genome of the dissimilatory IO_3_^−^-reducing bacterium *Denitromonas* sp. IR-12 (Reyes-Umana et al., 2022). Deletion of *idrA* gene impairs bacterial ability to grow with IO_3_^−^ as the sole terminal electron acceptor. The proposed functions of IdrABP_1_P_2_ from *Denitromonas* sp. IR-12 in IO_3_^−^ reduction are the same to those proposed for the IdrABP_1_P_2_ of *Pseudomonas* sp. strain SCT except that HIO is proposed to be disproportionate to IO_3_^−^ and I^−^. The IO_3_^−^ is further reduced by IdrAB (Reyes-Umana et al., 2022). Furthermore, microorganisms with *idrABP_1_P_2_* gene cluster are widespread in oceans, suggesting their global significance in biogeochemical cycling of iodine (Reyes-Umana et al., 2022).

The dissimilatory metal-reducing bacterium *Shewanella oneidensis* MR-1 reduces IO_3_^−^ extracellularly *via* DmsEFAB and MtrCAB (Mok et al., 2018; Toporek et al., 2019; Guo et al., 2022; Shin et al., 2022). The DmsEFAB is the first enzyme demonstrated experimentally for reducing IO_3_^−^ to HIO and H_2_O_2_ (Guo et al., 2022). Correspondingly, MtrCAB is also confirmed experimentally to reduce H_2_O_2_ to H_2_O for facilitating DmsEFAB-mediated IO_3_^−^ reduction. The Mtr extracellular electron transfer pathway is suggested to transfer electrons from the cytoplasmic membrane and across the periplasm to the DmsEF and MtrAB in the outer membrane. DmsEF and MtrAB transfer electrons across the outer membrane to DmsAB and MtrC, respectively. On bacterial surface, DmsAB and MtrC work collaboratively to reduce IO_3_^−^ (Guo et al., 2022).

Unlike *Pseudomonas* sp. strain SCT and *Denitromonas* sp. IR-12, *S. oneidensis* MR-1 is not an IO_3_^−^-respiring bacterium (Toporek et al., 2019). In *S. oneidensis* MR-1, DmsEFAB and MtrCAB are for extracellular respiration of DMSO and ferric iron [Fe(III)]-containing minerals, respectively (Beliaev and Saffarini, 1998; Beliaev et al., 2001; Gralnick et al., 2006; Bretschger et al., 2007; Coursolle and Gralnick, 2010). It is believed that their extracellular localization enables DmsEFAB and MtrCAB to reduce IO_3_^−^ collaboratively and extracellular reduction of IO_3_^−^ by *S. oneidensis* MR-1 is a fortuitous function (Guo et al., 2022). The non-IO_3_^−^ -respiring bacteria with the capability for extracellular reduction of IO_3_^−^ are also believed to impact the fate, transport, and global biogeochemical cycling of iodine (Guo et al., 2022). However, to what extent the bacteria with the capability for extracellular reduction of IO_3_^−^ distribute around the world has never been investigated before.

Like *idrABP_1_P_2_* gene cluster, the genes for DmsEFAB and MtrCAB are also clustered together, respectively (Gralnick et al., 2006; Fredrickson et al., 2008; Wang et al., 2008). In this investigation, we searched bacterial genomes for *dmsEFAB* and *mtrCAB* gene clusters and found that the bacteria with both *dmsEFAB* and *mtrCAB* gene clusters were widespread, showing global distribution of the bacteria possessing capability for extracellular reduction of IO_3_^−^. Worldwide occurrence of the bacteria with capability for extracellular reduction of IO_3_^−^ suggests the importance of these bacteria in global biogeochemical cycling of iodine.

## Approach

### Identification of *dmsEFAB* and *mtrCAB* homologs

DmsE and DmsF are homologous to MtrA and MtrB, respectively (Gralnick et al., 2006). Thus, we searched microbial genomes for DmsE/MtrA and DmsF/MtrB homologs by the approach that was described before (Shi et al., 2012a, 2014; Zhong and Shi, 2018). The DmsE, DmsF, MtrA, and MtrB of *S. oneidensis* MR-1served as templates for identifying microbial open reading frames (ORFs) whose protein sequences shared similarity to the templates by BLAST programs of the National Center for Biotechnology Information (NCBI) and of the Universal Protein Resource (UniProt; *E* < 0.01; Altschul et al., 1990), in which scoring matrix = BLOSUM62, gapopen = 0, gapextend = 0 and databases = non-redundant protein sequences database (nr) and UniprotKB database. The in-house Perl scripts and a hidden Markov model-based PRED-TMBB software were used to verify identified homologs with the CX_2_CH motifs and the trans-outer membrane motifs, respectively (Bagos et al., 2004;Shi et al., 2012b; Shi et al., 2014; Zhong and Shi, 2018). After verification, the identified homologs served as the templates for next round of genome search. The polypeptide sequences from the genes immediately upstream and downstream of the identified *dmsEF*/*mtrAB* gene clusters were further compared with previously identified DmsA, DmsB, and MtrC. The identified *dmsEFAB* gene clusters and the *mtrCAB* gene clusters co-existed with *dmsEFAB* gene clusters in the same bacterial genome were subjected to the additional analyses.

### Phylogenetic reconstruction and identification of additional genes for *c*-Cyts

Clustal W (version 2.1) was used to align the polypeptide sequences identified. The parameters used were Gap Opening Penalty = 10; Gap Extension Penalty = 0.2; Protein matrix = BLOSUM series (Larkin et al., 2007). MEGA7 was used to analyze the aligned sequences of DmsA, DmsB, DmsE/MtrA, DmsF/MtrB, or MtrC homologs. Phylogenetic trees were constructed with Maximum Likelihood at a confidence level determined by 1,000 bootstrap replications (Kumar et al., 2016). The results of phylogenetic reconstruction were displayed with Evolview v2 (He et al., 2016). The genes for *c*-Cyts on the upstream and downstream of the *mtrCAB* gene clusters were also identified by the method described above (Shi et al., 2012b, 2014; Zhong and Shi, 2018). The map of distribution for the identified bacteria was constructed similarly to that described previously (Baker et al., 2022).

## Results and discussion

### Overviews

DmsE/MtrA is a decaheme *c*-Cyt that is inserted into the outer membrane porin protein DmsF/MtrB. The function of DmsF/MtrB is to insulate DmsE/MtrA from the outer membrane, which permits rapid electron transfer across the outer membrane by DmsE/MtrA (Hartshorne et al., 2009; White et al., 2013; Edwards et al., 2020). Thus, the identified microorganisms that possessed *dmsEFAB* and/or *mtrCAB* gene clusters were all the Gram-negative bacteria. Supplementary Table S1 listed the bacteria identified with both *dmsEFAB* and *mtrCAB* gene clusters from this investigation. These included three *Ferrimonas* and 26 *Shewanella* species. It should be noted that in *S. oneidensis* MR-1, *dmsEFAB* and *mtrCAB* gene clusters are for extracellular respiration of DMSO and Fe(III)-containing minerals, respectively (Beliaev and Saffarini, 1998; Beliaev et al., 2001; Gralnick et al., 2006; Bretschger et al., 2007; Coursolle et al., 2010). Involvement of *dmsEFAB* and *mtrCAB* gene clusters in extracellular reduction of IO_3_^−^ is fortuitous (Guo et al., 2022). Restrictive distribution of the bacteria with capability for extracellular reduction of IO_3_^−^ in *Ferrimonas* and *Shewanella* species may be attributed to this fortuitous function.

Possession of both *dmsEFAB* and *mtrCAB* gene clusters suggests that these bacteria are capable of reducing IO_3_^−^ extracellularly. This is consistent with the previous observations that in addition to *S. oneidensis* MR-1, other *Shewanella* species, such as *S. putrefaciens*, reduced IO_3_^−^ (Councell et al., 1997; Farrenkorf et al., 1997; Mok et al., 2018; Toporek et al., 2019; Guo et al., 2022; Shin et al., 2022).

### Global distribution

**Figure 1:**
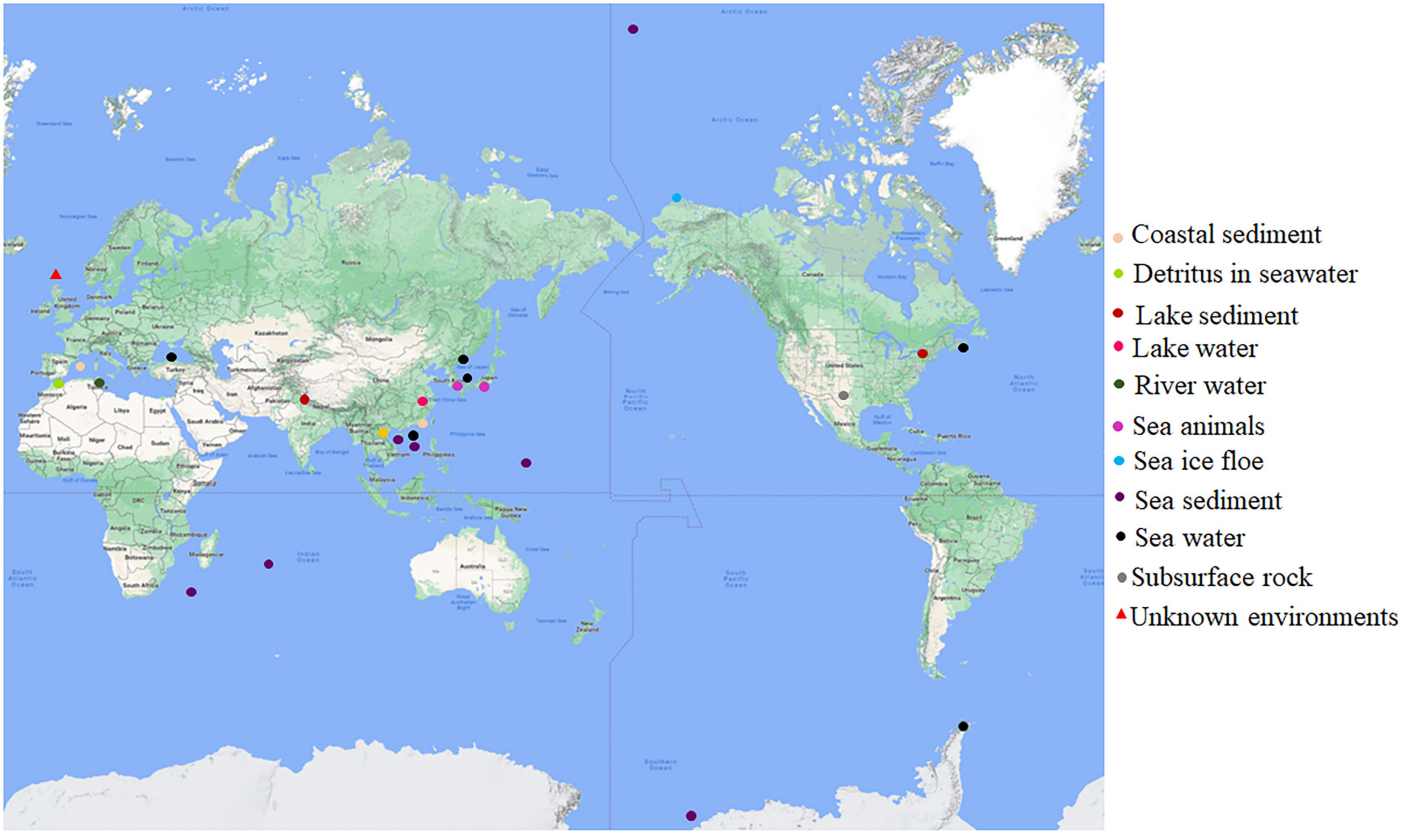
Global distribution of the bacteria with capability for extracellular reduction of iodate. Their original habits are indicated. See Supplementary Table S1 for details.

The original habitats for this group of bacteria with both *dmsEFAB* and *mtrCAB* gene clusters varied substantially (Figure 1; Supplementary Table S1). Some of them were isolated from the sediments of the lakes located in China, India, and the United States (Myers and Nealson, 1988; Li et al., 2014; Rathour et al., 2021); city drainage in Vietnam (Dao et al., 2022); subsurface rock in United States (Fredrickson et al., 1998), river water in Tunisia and rainbow trout in South Korea (Figure 1; Supplementary Table S1). Notably, >82% of the identified bacteria with *dmsEFAB* and *mtrCAB* gene clusters were isolated from a variety of marine environments around the world. These included the costal sediments in Mallorca, Spain; Xiamen, China, and Nova Scotia, Canada (Rossello-Mora et al., 1995; Zhao et al., 2005; Huang et al., 2010); sediments in Ross Sea, South China Sea, and Arctic Ocean (Ivanova et al., 2003; Hwang et al., 2019; Li et al., 2021); a cold seep field in South China Sea (Figure 1; Supplementary Table S1) and deep-sea sediments in Southwest Indian Ocean and West Pacific Ocean (Wang et al., 2004, 2021; Yu et al., 2021). Some were also found in seawater in East Sea, Korea; North Sea, United Kingdom; Troitsa Bay, Russia; Alboran Sea and Black Sea (Makemson et al., 1997; Reid and Gordon, 1999; Venkateswaran et al., 1999; Kim et al., 2017; Bae et al., 2021) as well as sea ice floe close to Point Barrow, Alaska, United States (Figure 1; Supplementary Table S1); the gastric cavity of galaxy coral in the coastal area near Hainan Island, China (Tang et al., 2020), and the intestines of sea animals in Japan (Satomi et al., 2003). Thus, bacteria with both *dmsEFAB* and *mtrCAB* gene clusters occur globally. Distribution of the bacteria with *dmsEFAB* and *mtrCAB* gene clusters is also comparable to the distribution of bacteria with porin-cytochrome gene clusters (Baker et al., 2022). Widespread occurrence of these bacteria around the world, especially their distribution in a variety of marine environments, suggests significant importance of the bacteria with capability for extracellular reduction of IO_3_^−^ in global biogeochemical cycling of iodine.

#### DmsA and DmsB homologs

**Figure 2:**
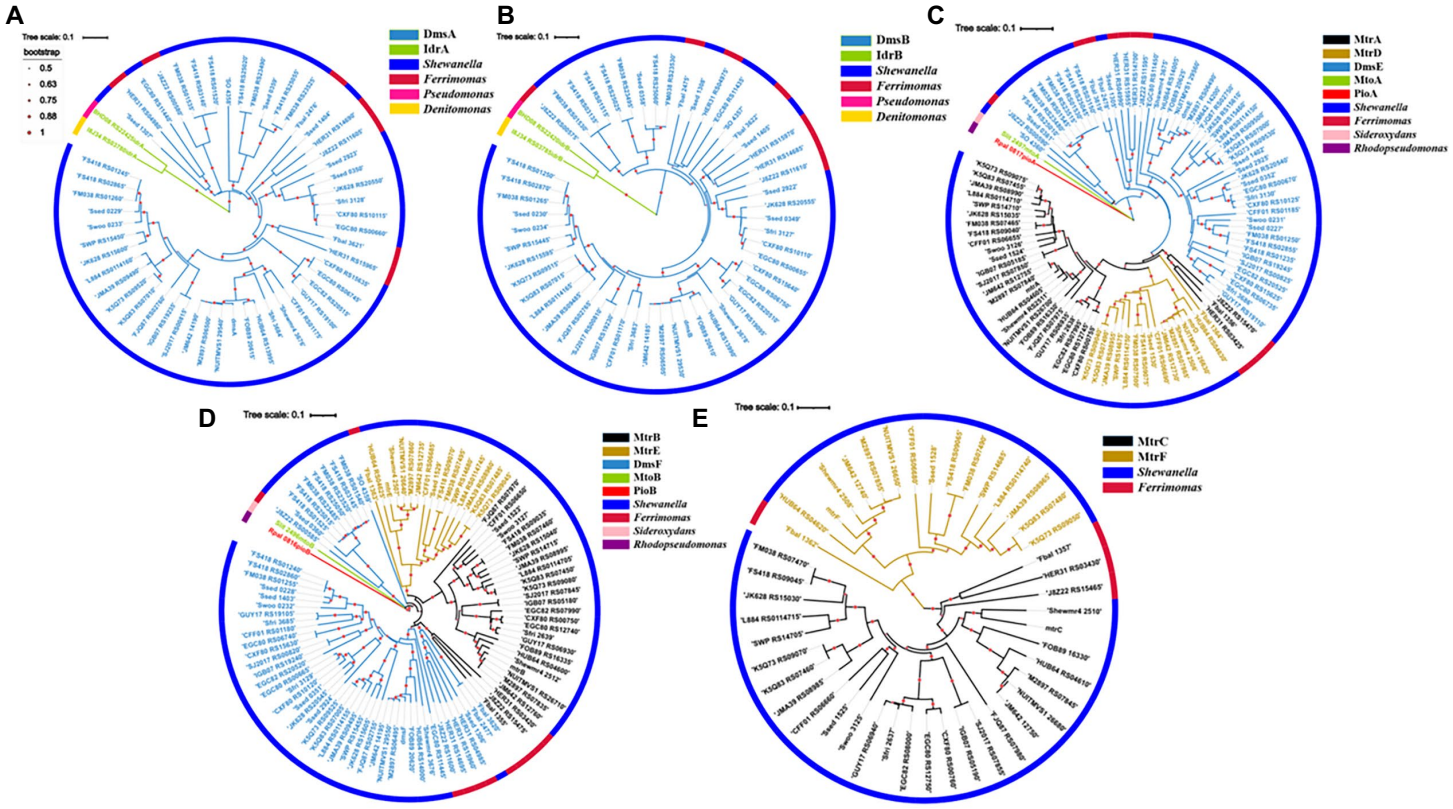
Phylogenetic analyses of identified DmsEFAB and MtrCAB homologs. **(A)** DmsA homologs, **(B)** DmsB homologs, **(C)** DmsE/MtrA/MtrD homologs, **(D)** DmsF/MtrB/MtrE homologs and **(E)**. MtrC/MtrF homologs. Construction and graphical display of phylogenetic trees were described in text. The outer layer indicates bacterial genera with identified DmsEFAB and MtrCAB homologs. For comparison, IdrAB of *Pseudomonas* sp. strain SCT and *Denitromonas* sp. IR-12, MtoAB of *Sideroxydans lithotrophicus* ES-1, and PioAB of *Rhodopseudomonas palustris*TIE-1 were included for the analyses.

A total of 52 DmsA homologs were identified from the bacteria with *dmsEFAB* and *mtrCAB* gene clusters (Figure 2A; Supplementary Table S2). For comparison, IdrA of *Pseudomonas* sp. strain SCT and *Denitromonas* sp. IR-12 were included for phylogenetic analyses (Yamazaki et al., 2020; Reyes-Umana et al., 2022). Like DmsA and SO_4358 of *S. oneidensis* MR-1, all the identified DmsA homologs possessed the twin-arginine sequence at their N-termini for their secretion to the periplasm *via* the twin-arginine protein secretion system (Supplementary Figure S1; Gralnick et al., 2006). The identified DmsA homologs were 35–98% identical to DmsA of *S. oneidensis* MR-1. Notably, SO_4358 of *S. oneidensis* MR-1 was 36% identical to DmsA of *S. oneidensis* MR-1 (Supplementary Table S2). SO_4358 is not involved in extracellular reduction of iodate by *S. oneidensis* MR-1 (Guo et al., 2022). The identified DmsA homologs were distantly related to IdrA of *Pseudomonas* sp. strain SCT and *Denitromonas* sp. IR-12 (Figure 2A; Supplementary Table S2).

The twin-arginine sequence was also detected in the DmsB homologs identified (Supplementary Figure S2; Gralnick et al., 2006). These DmsB homologs were 52%–99% identical to DmsB of *S. oneidensis* MR-1. Among them, SO_4359 of *S. oneidensis* MR-1 was 61% identical to DmsB of *S. oneidensis* MR-1 (Supplementary Table S3). The identified DmsB homologs were 11%–24% identical to IdrB of *Pseudomonas* sp. strain SCT and *Denitromonas* sp. IR-12 (Figure 2B; Supplementary Table S3).

#### DmsE/MtrA/MtrD and DmsF/MtrB/MtrE homologs

In *S. oneidensis* MR-1, MtrDEF are homologous to MtrABC, respectively, and *mtrDEF* genes are clustered together and are part of *mtrCAB* gene cluster (Fredrickson et al., 2008). However, the *mtrDEF* gene cluster is not involved in extracellular reduction of IO_3_^−^ by *S. oneidensis* MR-1 (Guo et al., 2022). Ninety-seven DmsE/MtrA/MtrD and DmsF/MtrB/MtrE homologs were identified, respectively, from the bacteria with *dmsEFAB* and *mtrCAB* gene cluster (Figures 2C,D; Supplementary Tables S4, S5). Identified DmsE/MtrA/MtrD homologs were 47%–98% identical to DmsE/MtrA/MtrD of *S. oneidensis* MR-1 (Supplementary Table S4), respectively; while the identified DmsF/MtrB/MtrE homologs were 24%–96% identical to DmsF/MtrB/MtrE of *S. oneidensis* MR-1, respectively (Supplementary Table S5). These DmsE/MtrA/MtrD and DmsF/MtrB/MtrE homologs were 32%–43% and 14%–23% identical to MtoA/PioA and MtoB/PioB of the Fe(II)-oxidizing bacteria *Sideroxydans lithotrophicus* ES-1 and *Rhodopseudomonas palustris*TIE-1, respectively (Figures 2C,D; Supplementary Tables S4, S5; Jiao et al., 2005; Jiao and Newman, 2007; Liu et al., 2012; Shi et al., 2012b; Liu et al., 2013). Previous results showed that MtoA/PioA and MtoB/PioB of *S. lithotrophicus* ES-1 and *R. palustris*TIE-1were homologous to MtrA and MtrB of *S. oneidensis* MR-1, respectively (Jiao and Newman, 2007; Shi et al., 2012b; Liu et al., 2014). Purified MtoA of *S. lithotrophicus* ES-1 was capable of oxidizing Fe(II), including the solid phase Fe(II) (Liu et al., 2012, 2013). PioA and PioB were also involved in extracellular oxidation of Fe(II) by *R. palustris*TIE-1 (Jiao and Newman, 2007). Given that they are more homologous to DmsE/MtrA and DmsF/MtrB of *S. oneidensis* MR-1 than to MtoA/PioA and MtoB/PioB of *S. lithotrophicus* ES-1 and *R. palustris*TIE-1, the identified DmsE/MtrA/MtrD and DmsF/MtrB/MtrE homologs are most likely to mediate electron transfer from inside cells to outside cells.

#### MtrC/MtrF homologs

Forty-five MtrC/MtrF homologs were identified from the bacteria with *dmsEFAB* and *mtrCAB* gene clusters (Figure 2E; Supplementary Figure S3; Supplementary Table S6). These homologs were 29%–71% identical to MtrC of *S. oneidensis* MR-1 (Supplementary Table S6). MtrC of *S. oneidensis* MR-1is a lipoprotein with 10 *c*-type hemes and is located on the bacterial surface (Shi et al., 2006, 2008; Lower et al., 2009; Edwards et al., 2020). It contains a lipid-binding site in its N-terminus and replacement of this site renders MtrC unable to bind to the outer membrane (Edwards et al., 2015). Similar to MtrC of *S. oneidensis* MR-1, all identified homologs possessed this lipid-binding site in their N-termini (Supplementary Figure S3). Thus, these MtrC/MtrF homologs are most likely on the bacterial cell surface. All the identified MtrC/MtrF homologs also possessed 10 *c*-type heme-binding sites (Supplementary Figure S3). Similar to MtrC and other *c*-Cyts, these MtrC homologs should possess intrinsic peroxidase activity to degrade the H_2_O_2_ formed from extracellular reduction of IO_3_^−^ (Thomas et al., 1976; Shi et al., 2006; Guo et al., 2022).

### Genetic organization

**Figure 3:**
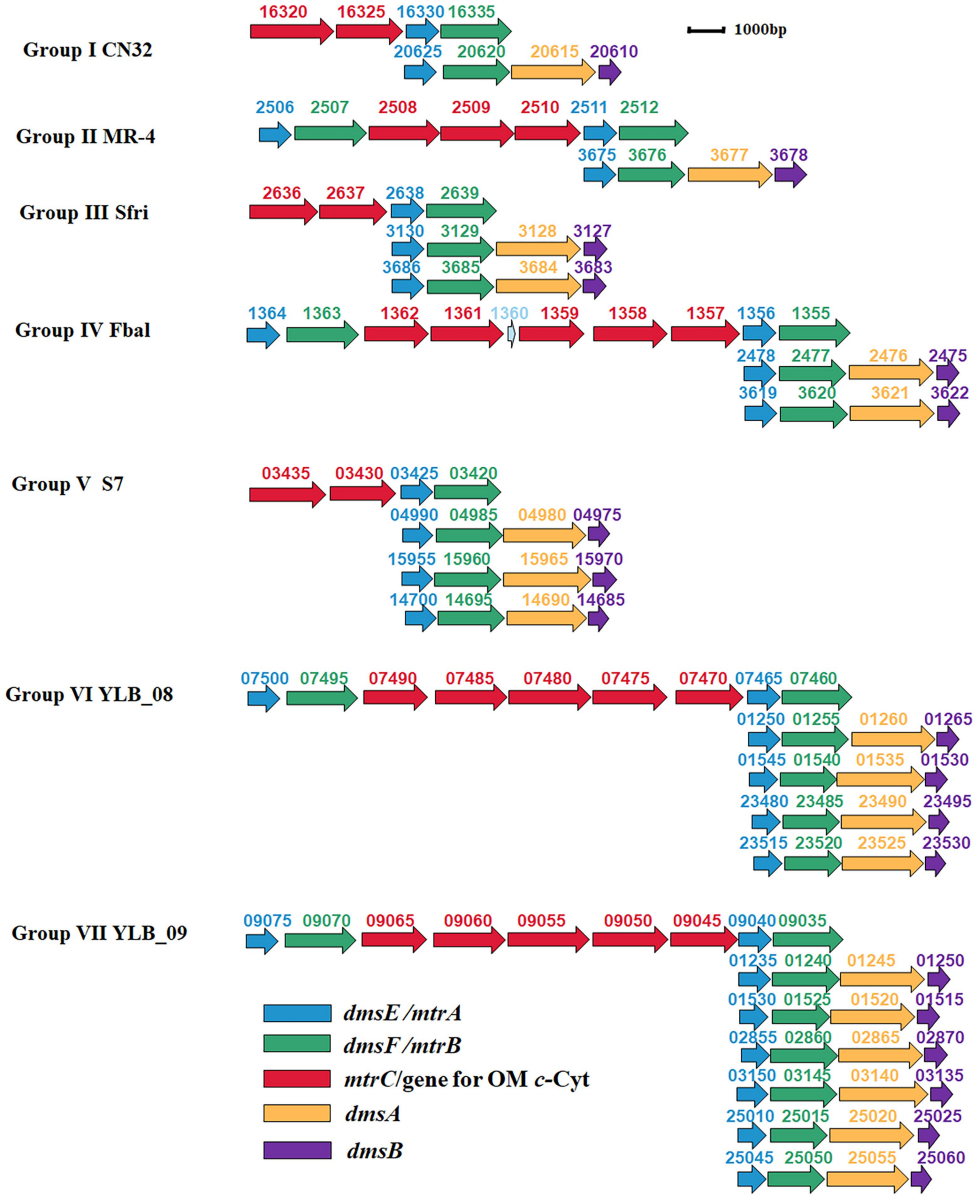
Genetic organization of identified *mtrCAB* and *dmsEFAB* gene clusters. The relative positions of genes identified within the complete genomes are shown. The identified genes are labeled with arrows. The arrow sizes indicate the relative lengths of identified genes. The arrow orientation indicates the presumed direction of gene transcription. The numbers above the identified genes are part of their locus tags. Group I: *S. japonica* KCTC 22435, *S. livingstonensis* LMG 19866, *S. putrefaciens* CN32 (CN32), *S. woodyi* ATCC 51908, *Shewanella* sp. ARC9_LZ, *Shewanella* sp. SUN WT4 and *Shewanella* sp. WPAGA9; Group II: *S. fidelis* ATCC-BAA-318, *S. marisflavi* EP1, *S. piezotolerans* WP3, *S. schlegeliana* JCM 11561, *S. xiamenensis* NUITM-VS1, *Shewanella* sp. 8A*, Shewanella* sp. ISTPL2, *Shewanella* sp. LZH-2, *Shewanella* sp. MBTL60-112-B1, *Shewanella* sp. MBTL60-112-B2 and *Shewanella* sp. MR-4 (MR-4); Group III: *Ferrimonas* sp. SCSIO 43195, *S. frigidimarina* NCIMB 400 (Sfri), *Shewanella* sp. Actino-trap-3 and *Shewanella* sp. KX20019; Group IV: *Ferrimonas balerica* DSM 9799 (Fbal) and *S. oneidensis* MR-1; Group V: *F. lipolytica* S7 (S7) and *S. psychromarinicola* M2; Group VI: *S. eurypsychrophilus* YLB-08 (YLB-08); and Group VII: *S. sediminis* HAW-EB3 and *Shewanella* sp. YLB-09 (YLB-09). OM *c*-Cyt: the outer membrane *c*-type cytochrome. See text for details.

Further investigation revealed that the numbers of *dmsEFAB* gene cluster and genes associated with *mtrCAB* gene cluster varied among the genomes of identified bacteria. Because of these differences, the identified bacteria could be categorized into seven different groups (Supplementary Table S1; Figure 3).

Group I bacteria contained a *dmsEFAB* gene cluster and a *mtrCAB* gene cluster. An additional gene for the outer membrane multiheme *c*-Cyt is also associated with the *mtrCAB* gene cluster (Group I, Figure 3). This group of bacteria included *S. japonica* KCTC 22435, *S. livingstonensis* LMG 19866, *S. putrefaciens* CN32, *S. woodyi* ATCC 51908, *Shewanella* sp. ARC9_LZ, *Shewanella* sp. SUN WT4, and *Shewanella* sp. WPAGA9 (Supplementary Table S1). Notably, the *dmsEFAB* and *mtrCAB* gene clusters of *S. japonica* KCTC 22435 were 99.5%–100% identical to those of *Shewanella* sp. WPAGA9, respectively (Figures 2A–E; Supplementary Tables S2–S6).

Like Group I bacteria, Group II bacteria also contained a *dmsEFAB* gene cluster and a *mtrCAB* gene cluster. However, the *mtrCAB* gene cluster of this group of bacteria also contained a *mtrDEF* gene cluster and the genes for other outer membrane multiheme *c*-Cyt (Group II, Figure 3). Group II bacteria included *S. fidelis* ATCC-BAA-318, *S. marisflavi* EP1, *S. piezotolerans* WP3, *S. schlegeliana* JCM 11561, *S. xiamenensis* NUITM-VS1, *Shewanella* sp. 8A*, Shewanella* sp. ISTPL2, *Shewanella* sp. LZH-2, *Shewanella* sp. MBTL60-112-B1, *Shewanella* sp. MBTL60-112-B2, and *Shewanella* sp. MR-4 (Supplementary Table S1). Among this group of bacteria, *Shewanella* sp. MBTL60-112-B1 and *Shewanella* sp. MBTL60-112-B2 shared identical *dmsEFAB* and *mtrCAB* gene clusters (Figures 2A–E; Supplementary Tables S2–S6). These results suggest that they are very closely related. Results also showed that *dmsEFAB* and *mtrCAB* gene clusters of *S. xiamenensis* NUITM-VS1, *Shewanella* sp. 8A, and *Shewanella* sp. LZH-2 were 97.6%–100% identical, respectively (Figures 2A–E; Supplementary Tables S2–S6), which suggest that these *Shewanella* spp. acquire *dmsEFAB* and *mtrCAB* gene clusters from a common ancestor.

Both Group III and IV bacteria contained two *dmsEFAB* gene clusters and a *mtrCAB* gene cluster. The major difference between these two groups of bacteria was that the *mtrCAB* gene of Group IV bacteria also had a *mtrDEF* gene cluster and one to three genes for the outer membrane *c*-Cyts (Group III and IV, Figure 3). Group III bacteria included *Ferrimonas* sp. SCSIO 43195, *S. frigidimarina* NCIMB 400, *Shewanella* sp. Actino-trap-3 and *Shewanella* sp. KX20019, among which an additional gene for the outer membrane multiheme *c*-Cyt associated with the *mtrCAB* gene cluster of *S. frigidimarina* NCIMB 400, *Shewanella* sp. Actino-trap-3 and *Shewanella* sp. KX20019 (Group III, Figure 3; Supplementary Table S1). The identified Group IV bacteria were *Ferrimonas balerica* DSM 9799 and *S. oneidensis* MR-1 (Supplementary Table S1).

Group V bacteria all possessed three *dmsEFAB* gene clusters and a *mtrCAB* gene cluster with an additional gene for the outer membrane *c*-Cyt (Group V, Figure 3), which included *F. lipolytica* S7 and *S. psychromarinicola* M2 (Supplementary Table S1). One of the *dmsEFAB* gene clusters, the *06735–06750* gene cluster, of *S. psychromarinicola* M2 was 100% identical to the *15625–15640* gene cluster, one of the *dmsEFAB* gene clusters of *Shewanella* sp. Actino-trap-3 from Group III (Figures 2A–D; Supplementary Tables S2–S5). Similarly, *mtrCAB* gene cluster of *S. psychromarinicola* M2 was 97.1%–99.7% identical to that of *Shewanella* sp. Actino-trap-3 (Figures 2C–E; Supplementary Tables S4–S6).

Both Group VI and VII bacteria had a *mtrCAB* gene cluster that also included a *mtrDEF* gene cluster and three genes for the outer membrane *c*-Cyts (Group VI and VI, Figure 3). However, the Group VI bacterium *S. eurypsychrophilus* YLB-08 possessed four *dmsEFAB* gene clusters (Group VI, Figure 3; Supplementary Table S1), while the Group VII bacteria *S. sediminis* HAW-EB3 and *Shewanella* sp. YLB-09 contained six *dmsEFAB* gene clusters (Group VII, Figure 3; Supplementary Table S1). Notably, among the six *dmsEFAB* gene clusters of *Shewanella* sp. YLB-09, the *01235–01250* and *01530–01515* gene cluster were 100% identical to the *02855–02870* and *03150–03135* gene clusters, respectively, which demonstrates duplications of *dmsEFAB* gene clusters in *Shewanella* sp. YLB-09 (Figures 2A–D; Group VII, Figure 3; Supplementary Tables S2–S5). Furthermore, the *mtrCAB* gene cluster and its associated genes (*07500–07460*) of *S. eurypsychrophilus* YLB-08 were 100% identical to the *mtrCAB* gene cluster and its associated genes (*09075–09035*) of *Shewanella* sp. YLB-09 (Group VI and VII, Figure 3; Supplementary Tables S4–S6). Similarly, *dmsEFAB* gene clusters *01250–01265*, *01545–01530*, *23480–23495*, and *23515–23530* of *S. eurypsychrophilus* YLB-08 were 100% identical to *dmsEFAB* gene clusters *01235–01250/02855–02870*, *01530–01515/03150–03135*, *25010–25025* and *25045–25060* of *Shewanella* sp. YLB-09, respectively (Group VI and VII, Figure 3; Supplementary Tables S2–S5). Thus, the *dmsEFAB* and *mtrCAB* gene clusters of *S. eurypsychrophilus* YLB-08 and *Shewanella* sp. YLB-09 must be acquired from the same ancestor.

## Conclusion

To investigate to what extent the extracellular IO_3_^−^-reducing organisms were distributed around world, the bacteria with both *dmsEFAB* and *mtrCAB* gene clusters were systemically searched. A total of 29 bacteria were identified to possess both gene clusters. They belonged to the genus of *Ferrimonas* and *Shewanella*. Possession of both *dmsEFAB* and *mtrCAB* gene clusters suggests the ability to mediate extracellular reduction of IO_3_^−^ by these bacteria. The identified bacteria with capability for extracellular reduction of IO_3_^−^ were widespread around the world. Although some were found in freshwater lakes and rivers as well as the rocks of deep continental subsurface, most of them were derived from geographically distributed marine environments. The latter included those found in the Arctic, Atlantic, Indian, and Pacific oceans. Widespread occurrence of the bacteria with capability for extracellular reduction of IO_3_^−^ suggests a crucial role of this group of bacteria in global biogeochemical cycling of iodine.

The genetic organizations of identified *dmsEFAB* and *mtrCAB* gene clusters varied significantly. The *mtrCAB* gene clusters often associated with genes for the outer membrane *c*-Cyt of multiheme. Some of the *mtrCAB* gene clusters also contained a *mtrDEF* gene cluster. The numbers of *dmsEFAB* gene cluster detected in a given bacterial genome ranged from one to six. Duplications of the detected *dmsEFAB* gene clusters also occurred. Thus, this group of bacteria acquire their capability for extracellular reduction of iodate differently.

Collectively, the results from this investigation provide new insights into the distribution and evolution of as well as the role in global biogeochemical cycling of iodine by the bacteria with capability for extracellular reduction of iodate. Physiological characterization of the iodate-reducing capacity for the predicted strains and their ecological roles on iodine cycling in different ecosystems are in need of further investigation.

## Data availability statement

The original contributions presented in the study are included in the article/Supplementary material, further inquiries can be directed to the corresponding authors.

## Author contributions

LS designed the experiment and acquired funding. JG, JJ, and YZ performed the experiment. ZP, YJ, ZJ, YH, YD, and LS analyzed the data and prepared manuscript. All authors contributed to the article and approved the submitted version.

## Funding

We would like to thank the National Key Research and Development Program of China (2018YFA0901303), National Natural Science Foundation of China (NSFC91851211, 42277065, 42272353), and the Fundamental Research Funds for the Universities of Chinese Central Government, China University of Geosciences-Wuhan (122-G1323522144) for their support.

## Conflict of interest

The authors declare that the research was conducted in the absence of any commercial or financial relationships that could be construed as a potential conflict of interest.

## Publisher’s note

All claims expressed in this article are solely those of the authors and do not necessarily represent those of their affiliated organizations, or those of the publisher, the editors and the reviewers. Any product that may be evaluated in this article, or claim that may be made by its manufacturer, is not guaranteed or endorsed by the publisher.

## Supplementary material

The Supplementary material for this article can be found online at: https://www.frontiersin.org/articles/10.3389/fmicb.2022.1070601/full#supplementary-material
