# Supplementary material for: Global occurrence of the bacteria with capability for extracellular reduction of iodate
Source: Front Microbiol. 2022 Nov 25;13:1070601. doi: 10.3389/fmicb.2022.1070601 (PMC9732548; doi:10.3389/fmicb.2022.1070601)
Supplement: Supplementary file 1 [file Table_1.DOCX]

**Global Occurrence of the Bacteria with Capability for Extracellular Reduction of Iodate**

Jinzhi Guo^1,#^, Jie Jiang^1,#^, Zhaofeng Peng^1,^*, Yuhong Zhong^1^, Yongguang Jiang^1^, Zhou Jiang^1,2^, Yidan Hu^1^, Yiran Dong^1,2,3,4^ and Liang Shi^1,2,3,4,^*

^1^Department of Biological Sciences and Technology, School of Environmental Studies;^2^State Key Laboratory of Biogeology and Environmental Geology; ^3^Hubei Key Laboratory of Yangtze Catchment Environmental Aquatic Science; and ^4^State Environmental Protection Key Laboratory of Source Apportionment and Control of Aquatic Pollution, Ministry of Ecology and Environment; China University of Geosciences, Wuhan, Hubei, 430074, People’s Republic of China

**Supplemental information**

Table S1. The bacteria identified with both *dmsEFAB* and *mtrCAB* gene clusters.

| Name | Isolation Sites | References | Group# |
| --- | --- | --- | --- |
| *Ferrimonas balerica* DSM 9799 | Coastal sediment, Mallorca, Spain | (Rossello-Mora et al., 1995) | IV |
| *F. lipolytica* S7 | Seawater, Uljin marina, East Sea, Korea | (Bae et al., 2021) | V |
| *Ferrimonas* sp. SCSIO 43195 | Gastric cavity of *Galaxea fascicularis*, Hainan Island, China | (Tang et al., 2020) | III |
| *Shewanella eurypsychrophilus* YLB-08 | Deep-sea sediment, Southwest Indian Ocean | (Yu et al., 2021) | VI |
| *S. fidelis* ATCC-BAA-318 | Sediment, South China Sea | (Ivanova et al., 2003) | II |
| *S. frigidimarina* NCIMB 400 | North Sea, UK | (Reid and Gordon, 1999) | III |
| *S. marisflavi* EP1 | Costal sediment, Xiamen, China | (Huang et al., 2010) | II |
| *S. japonica* KCTC 22435 | Sea water, Troitza Bay, Russia | (Kim et al., 2017) | I |
| *S. livingstonensis*  LMG 19866 | Antarctic coastal marine environments | (Bozal et al., 2002) | I |
| *S. oneidensis* MR-1 | Sediment, Lake Oneida, NY, US | (Myers and Nealson, 1988) | IV |
| *S. piezotolerans* WP3 | Deep sea sediment, West Pacific Ocean | (Wang et al., 2004) | II |
| *S. psychromarinicola* M2 | Pelagic surface-sediment, Ross Sea, Antarctica | (Hwang et al., 2019) | V |
| *S. putrefaciens* CN32 | Subsurface rock, Cerro Negro, NM, US | (Fredrickson et al., 1998) | I |
| *S. schlegeliana* JCM 11561 | Sea animal intestines, Japan | (Satomi et al., 2003) | II |
| *S. sedimins* HAW-EB3 | Sediment, Halifax Harbour, Nova Scotia, Canada | (Zhao et al., 2005) | VII |
| *S. woodyi* ATCC 51908 | Detritus in seawater; Alboran Sea | (Makemson et al., 1997) | I |
| *S. xiamenensis* NUITM-VS1 | Urban drainage, Hanoi, Vietnam, | (Dao et al., 2022) | II |
| *Shewanella* sp. 8A | River water, Tunisia | NCBI | II |
| *Shewanella* sp. Actino-trap-3 | Sea ice floe, Point Barrow, AK, US | NCBI | III |
| *Shewanella* sp. ARC9_LZ | Marine sediment, Arctic Ocean | (Li et al., 2021) | I |
| *Shewanella* sp. ISTPL2 | Sediment, Pangong Lake, India | (Rathour et al., 2021) | II |
| *Shewanella* sp. KX20019 | Cold seep field, South China Sea | NCBI | III |
| *Shewanella* sp. LZH-2 | Lake Taihu, China | (Li et al., 2014) | II |
| *Shewanella* sp. MBTL60-112-B1 | NA* | NCBI | II |
| *Shewanella* sp. MBTL60-112-B2 | NA | NCBI | II |
| *Shewanella* sp. MR-4 | Oxic zone of water column, Black Sea | (Venkateswaran et al., 1999) | II |
| *Shewanella* sp. SUN WT4 | Kidney of diseased rainbow trout, Jeonbuk province, South Korea | NCBI | I |
| *Shewanella* sp. WPAGA9 | Deep-sea sediment | (Wang et al., 2021) | I |
| *Shewanella* sp. YLB-09 | Deep-sea sediment, Southwest Indian Ocean | (Yu et al., 2021) | VII |

#Group based on genetic organization, *NA, not available.

Table S2. Similarity analysis of identified DmsA homologs. For comparison, IdrA of *Pseudomonas* sp. strain SCT and *Denitromonas* sp. IR-12 are included.

Table S3. Similarity analysis of identified DmsB homologs. For comparison, IdrB of *Pseudomonas* sp. strain SCT and *Denitromonas* sp. IR-12 are included.

Table S4. Similarity analysis of identified DmsE/MtrA/MtrD homologs. For comparison, MtoA of *S. lithotrophicus* ES-1 and PioA of *R. palustris*TIE-1 are included.

Table S5. Similarity analysis of identified DmsF/MtrB/MtrE homologs. For comparison, MtoB of *S. lithotrophicus* ES-1 and PioB of *R. palustris*TIE-1 are included.

Table S6. Similarity analysis of identified MtrC/MtrF homologs.

Figure S1. Sequence alignment of identified DmsA homologs. For comparison, IdrA of *Pseudomonas* sp. strain SCT and *Denitromonas* sp. IR-12 are included.

Figure S2. Sequence alignment of identified DmsB homologs. For comparison, IdrB of *Pseudomonas* sp. strain SCT and *Denitromonas* sp. IR-12 are included.

Figure S3. Sequence alignment of identified MtrC/MtrF homologs.
