# Supplementary material for: Global occurrence of the bacteria with capability for extracellular reduction of iodate
Source: Front Microbiol. 2022 Nov 25;13:1070601. doi: 10.3389/fmicb.2022.1070601 (PMC9732548; doi:10.3389/fmicb.2022.1070601)
Supplement: Supplementary file 7 [file Data_Sheet_1.PDF]

```
                                                              .         .         .         .
S_oneidensis_MR-1_dmsA                                   1 ......................MERRSFLKMSAAMGCAATVTGCNSGSSDVNV..VPP
S_oneidensis_MR-1_SO_4358                                1 ......................MKRRAFLKMSASCAAAATVVGCGSD..KNPVLKP.E
S_sp._LZH-2_JM642_14190                                  1 ......................MERRSFLKMSAAMGCAATVTGCNSGSSDVNV..VPP
S_xiamenensis_NUITM-VS1_NUITMVS1_29540                   1 ......................MERRSFLKMSAAMGCAATVTGCNSGSSDVNV..VPP
S_putrefaciens_strain_FDAARGOS_681_FOB89_20615           1 ......................MERRSFLKMSAAMGCAATVTGCNSGSSDVNV..VPP
S_sp._MR-4_Shewmr4_3676                                  1 ......................MERRSFLKMSAALSAAAAVTGCDSSSKDVDV..VPP
S_fidelis_ATCC-BAA-318__L884__L884_RS0114160             1 ......................MERRSFLKMSAALGCAATVTGCNSSSDDANV..IPP
S_piezotolerans_WP3_SWP_RS15450                          1 ......................MERRSFLKMSAALGCAATVTGCNSSSDDANV..IPP
S_schlegeliana_strain_JCM_JMA39_11561_RS09490            1 ......................MERRSFLKMSAALGCAATVTGCNSSSDDANV..IPP
S_marisflavi_strain_EP1_CFF01_RS01175                    1 ......................MERRSFLKASAALGCAATVTGCKTSSDEPNK..VPP
S_sp.SUN_WT4_FJQ87_RS02760                               1 ......................MERRSFLKMSAAMSCAATVTGCNSSSKDANL..VPP
S_sp.MBTL60-112-B2_K5Q73_RS09520                         1 ......................MERRSFLKMSAALGCAATVTGCNSSSDDANV..IPP
S_sp.MBTL60_112_B1_K5Q83_RS07010                         1 ......................MERRSFLKMSAALGCAATVTGCNSSSDDANV..IPP
S_enrypsychrophilus_strain_YLB-08_FM038_RS01260          1 ......................MERRSFLKMSAAMGCAASVTGCKTSSDDANV..VPP
S_enrypsychrophilus_strain_YLB-08_FM038_RS01535          1 ......................MKRRDFLKVSAASAAVASVTGCSSDSAEPAKPIEQP
S_enrypsychrophilus_strain_YLB-08_FM038_RS23490          1 ......................MKRRDFLKVSAAAAAATAITGCDSSSAEDPILNPGV
S_enrypsychrophilus_strain_YLB-08_FM038_RS23525          1 ......................MERRTFLKFSAAAAAVATVTGCESSSASDPLPTPSE
S_sp.YLB_09_FS418_RS01245                                1 ......................MERRSFLKMSAAMGCAASVTGCKTSSDDANV..VPP
S_sp.YLB_09_FS418_RS01520                                1 ......................MKRRDFLKVSAASAAVASVTGCSSDSAEPAKPIEQP
S_sp.YLB_09_FS418_RS02865                                1 ......................MERRSFLKMSAAMGCAASVTGCKTSSDDANV..VPP
S_sp.YLB_09_FS418_RS03140                                1 ......................MKRRDFLKVSAASAAVASVTGCSSDSAEPAKPIEQP
S_sp.YLB_09_FS418_RS25020                                1 ......................MKRRDFLKVSAAAAAATAITGCDSSSAEDPILNPGV
S_sp.YLB_09_FS418_RS25055                                1 ......................MERRTFLKFSAAAAAVATVTGCESSSASDPLPTPSE
S_sp._WPAGA9_IGB07_RS19235                               1 ......................MERRSFLKMSAALGCAATVTGCNSSSKDAEP..LPP
S_sp._ARC9_LZ_GUY17_RS19100                              1 ......................MERRNFLKMSAAIGAAATITGCEDSAKEINV..VPP
S_psychromarinicola_strain_M2_EGC80_RS00660              1 ......................MERRSFLKMSAALSCVATVSGCNSSSKDVEV..VPP
S_psychromarinicola_strain_M2_EGC80_RS06745              1 ......................MERRNFLKMSAAIGAAATITGCEDSAKEINV..IPP
S_psychromarinicola_strain_M2_EGC80_RS11440              1 ......................MERRDFLKVSASIAALSSITACNTTSEDSEE.....
S_sp.Actino-trap-3_CXF80_RS10115                         1 ......................MERRSFLKMSAALSCVATVSGCNSSSKDVEV..VPP
S_sp.Actino-trap-3_CXF80_RS15635                         1 ......................MERRNFLKMSAAIGAAATITGCEDSAKEINV..IPP
S_livingstonensis_strain_LMG_19866_EGC82_RS20515         1 ......................MERRSFLKMSAAMGAAATITGCENSTKEINV..VEP
Ferrimonas_lipolytica_strain_S7_HER31_RS14690            1 ......................MERRNFLKLSATAGAVSCITACGSKSSNS....STP
Ferrimonas_lipolytica_strain_S7_HER31_RS15965            1 ......................MQRRDFLKLSATAGAVSCITACGSKSSDS......P
Ferrimonas_lipolytica_strain_S7_HER31_RS04980            1 ......................MQRRDFLKLSAATGTVSCVTACGSKSNDEPV.....
S_sp.ISTPL2_CCLCJOKE_1_HUB64_RS13995                     1 ......................MERRSFLKISAAMGCAATVTGCNSGSSDVDV..VPP
S_japonica_strain_KCTC_22435_SJ2017_RS00815              1 ......................MERRSFLKMSAALGCAATVTGCNSSSKDAEP..LPP
S_sp.8A_M2897_RS06500                                    1 ......................MERRSFLKMSAAMGCAATVTGCNSGSSDVNV..VPP
S_woodyi_ATCC_51908_Swoo_0233                            1 ......................MERRSFLKMSAALGCAASVTGCKTSSDDANV..VPP
Ferrimonas_balerica_DSM_9799_Fbal_2476                   1 ......................MQRRDFLKSAALVSAAASLAGCNGKRTETVI..PEP
Ferrimonas_balerica_DSM_9799_Fbal_3621                   1 ......................MQRRDFLKLSATAGAVSCLSACGGKDTEN......V
S_frigidimarina_NCIMB_400_Sfri_3128                      1 ......................MERRSFLKMSAALSCVATVSGCNSSSKDVEV..VPP
S_frigidimarina_NCIMB_400_Sfri_3684                      1 ......................MERRSFLKASAALSCVATVTGCNTSSDDANV..IPP
S_sediminis_HAW-EB3_Ssed_0229                            1 ......................MERRSFLKMSAAMGCAASVTGCKTSSDDANV..VPP
S_sediminis_HAW-EB3_Ssed_0350                            1 ......................MERRSFLKMSAALSCAATVSGCNSSSKDVEV..VPP
S_sediminis_HAW-EB3_Ssed_0359                            1 ......................MERRTFLKFSAAAAAVATVTGCESSSASNPLPAPSE
S_sediminis_HAW-EB3_Ssed_1307                            1 ......................MQRREFLKLSAAASAVTCVTACGSK.KTETI.....
S_sediminis_HAW-EB3_Ssed_1404                            1 ......................MQRRDFLKLSAAAGAVSCVTACGSKKTET....VIP
S_sediminis_HAW-EB3_Ssed_2923                            1 ......................MERRSFLKMSAAMGCAATVAGCNSSSSDVDV..VPP
S_sp.KX20019_JK628_RS15600                               1 ......................MERRSFLKMSAALGCAATVTGCNSSSDDANV..IPP
S_sp.KX20019_JK628_RS20550                               1 ......................MERRSFLKMSAALSCAATVSGCNSSSKDVEV..VPP
Ferrimonas_sp.SCSIO_43195_J8Z22_RS00580                  1 ......................MKRRDFLKVSAATAAAASVTGCSSDSADEAQPVEPP
Ferrimonas_sp.SCSIO_43195_J8Z22_RS11605                  1 ......................MERREFLKMSAAMSAAAAVTGCSSES..AQT..TPP
Pseudomonas_sp.SCT_BHD08_RS22425(idrA)                   1 MSK......PDEYLSS..NSVPLPPQDADVLTTACDYCIVACGYKVYRWPVGKEGGAK
Denitromonas_sp.IR12_I8J34_RS03780(idrA)                 1 MSENIKQGGAGTFMQAPQDSVPLPPKDAEVMTTACDYCTVACGYKVYRWPVGKEGGMK
```

```
                                                                                 .              .             .             .                   .
S_oneidensis_MR-1_dmsA                                          35  KPPVGD.EVQTWSSCLVNCGSNCPIKVFSRDGIITRIETDHET....NDEYG......
S_oneidensis_MR-1_SO_4358                                       34  VSPEQVPEISKLSACIGNCFQTCPLRVYSRNGIITRIESESGGDIKFVDNWSVDGA..
S_sp._LZH-2_JM642_14190                                         35  KPPVGD.EVQTWSSCLVNCGSNCPIKVFSRDGIITRIEADHET....SDEYG......
S_xiamenensis_NUITM-VS1_NUITMVS1_29540                          35  KPPVGD.EVQTWSSCLVNCGSNCPIKVFSRDGIITRIEADHET....SDEYG......
S_putrefaciens_strain_FDAARGOS_681_FOB89_20615                  35  KPPIGD.EIQTWSSCLVNCGSNCPIKVFSRDGVITRIEADHET....NDEYG......
S_sp._MR-4_Shewmr4_3676                                         35  TPPVTDGEIQTWSSCLVNCGSNCPIKVFSKDGVITRVEADNET....TDEYG....Y.
S_fidelis_ATCC-BAA-318__L884__L884_RS0114160                    35  QPPVTD.EVVNWSSCLVNCGSNCPIKVFTKDGIVTRVETDHEV....EDNYG......
S_piezotolerans_WP3_SWP_RS15450                                 35  KPPVGD.EQVTWSSCLVNCGSNCPVKVFSRDGVITRVETDHDI....TDGDD......
S_schlegeliana_strain_JCM_JMA39_11561_RS09490                   35  QPPVKD.EVVNWSSCLVNCGSNCPIKVFTKDGIVTRVETDHEV....EDNYG......
S_marisflavi_strain_EP1_CFF01_RS01175                           35  TPPVEE..NQTWSACLVNCGSNCPIKVFSRDGVITRVEADNET....TDEYG....H.
S_sp.SUN_WT4_FJQ87_RS02760                                      35  QPPVSE.EAINWSSCLVNCGSNCPIKVFSRDGVITRVETDHEV....DDVYG......
S_sp.MBTL60-112-B2_K5Q73_RS09520                                35  QPPVTD.EVVNWSSCLVNCGSNCPIKVFTKDGIVTRVETDHEV....EDEYG......
S_sp.MBTL60_112_B1_K5Q83_RS07010                                35  QPPVTD.EVVNWSSCLVNCGSNCPIKVFTKDGIVTRVETDHEV....EDEYG......
S_enrypsychrophilus_strain_YLB-08_FM038_RS01260                 35  KPPVGE.EQITWSSCLVNCGSNCPVKVFSRDGVITRVETDHDV....VDGDD......
S_enrypsychrophilus_strain_YLB-08_FM038_RS01535                 37  ITDPIAGESHAWAQCQSNCWAWCPLKVITKDGKIIRIEPHTE....GDDNFD....SG
S_enrypsychrophilus_strain_YLB-08_FM038_RS23490                 37  PVTPPTGEKITWSNCASNCFQSCPLKVHSKDGVVTRIEAEQ....KEHDDW..DTF..
S_enrypsychrophilus_strain_YLB-08_FM038_RS23525                 37  PVLPPVGEGVTWGSCATNCWQACPLKVHSKDGVITRIETEG....KATDDW..DSF..
S_sp.YLB_09_FS418_RS01245                                       35  KPPVGE.EQITWSSCLVNCGSNCPVKVFSRDGVITRVETDHDV....VDGDD......
S_sp.YLB_09_FS418_RS01520                                       37  ITDPIAGESHAWAQCQSNCWAWCPLKVITKDGKIIRIEPHTE....GDDNFD....SG
S_sp.YLB_09_FS418_RS02865                                       35  KPPVGE.EQITWSSCLVNCGSNCPVKVFSRDGVITRVETDHDV....VDGDD......
S_sp.YLB_09_FS418_RS03140                                       37  ITDPIAGESHAWAQCQSNCWAWCPLKVITKDGKIIRIEPHTE....GDDNFD....SG
S_sp.YLB_09_FS418_RS25020                                       37  PVTPPTGEKITWSNCASNCFQSCPLKVHSKDGVVTRIEAEQ....KEHDDW..DTF..
S_sp.YLB_09_FS418_RS25055                                       37  PVLPPVGEGVTWGSCATNCWQACPLKVHSKDGVITRIETEG....KATDDW..DSF..
S_sp._WPAGA9_IGB07_RS19235                                      35  QPPV.D.EALTWSACLVNCGSNCPVKVYSRDGMITRVETDHEV....EDVYG......
S_sp._ARC9_LZ_GUY17_RS19100                                     35  EVPESSLEQLNWSACLVNCGSNCPIKVFSKDGVITRIETDHET....TDEYG....V.
S_psychromarinicola_strain_M2_EGC80_RS00660                     35  TPPVAG.EKLNWSACLVNCGSNCPVQVFSTDGVITRVESQFTT....TDKYG......
S_psychromarinicola_strain_M2_EGC80_RS06745                     35  GVPESSLEQLNWSACLVNCGSNCPIKVFSKDGVITRIETDHET....TDEYG....V.
S_psychromarinicola_strain_M2_EGC80_RS11440                     32  VKELAPEEVTTMSSCNVNCKATCPLVITTVDGIITKVTPETTTI.DSEDGFV....IR
S_sp.Actino-trap-3_CXF80_RS10115                                35  TAPVAA.EKLNWSACLVNCGSNCPVQVFSTDGVITRVESQFTT....TDKFG......
S_sp.Actino-trap-3_CXF80_RS15635                                35  GVPESSLEQLNWSACLVNCGSNCPIKVFSKDGVITRIETDHET....TDEYG....V.
S_livingstonensis_strain_LMG_19866_EGC82_RS20515                35  AVPESSLEQLNWSACLVNCGSNCPIKVFSKDGVITRIETDHET....TDEYG....V.
Ferrimonas_lipolytica_strain_S7_HER31_RS14690                   33  TVPEVSEEQVNWAACTCNCGASCPIKIVTQDGTITRVETDDMG....DDSWA......
Ferrimonas_lipolytica_strain_S7_HER31_RS15965                   31  APVAPAEEQLNWTACLVNCGSNCPLKVYSVDGVCTRVETDWAE....SDDYG......
Ferrimonas_lipolytica_strain_S7_HER31_RS04980                   32  PTIPEVAEVETLSACEANCAATCAFKVISQDGVLKRVEQEN....FYEDGFD....IR
S_sp.ISTPL2_CCLCJOKE_1_HUB64_RS13995                            35  KPPIGD.EIQTWSSCLVNCGSNCPIKVFSRDGVITRIEADHET....NDEYG......
S_japonica_strain_KCTC_22435_SJ2017_RS00815                     35  QPPV.D.EALTWSACLVNCGSNCPVKVYSRDGMITRVETDHEV....EDVYG......
S_sp.8A_M2897_RS06500                                           35  KPPVGD.EVQTWSSCLVNCGSNCPIKVFSRDGIITRIEADHET....SDEYG......
S_woodyi_ATCC_51908_Swoo_0233                                   35  KPPVGE.EQITWSSCLVNCGSNCPVKVFSRDGVITRVETDHDV....VDGDD......
Ferrimonas_balerica_DSM_9799_Fbal_2476                          35  ELPVTQ....GWQSCYVNCGSNCPIKFTVQDGQITQLETDDHG....DDQYE......
Ferrimonas_balerica_DSM_9799_Fbal_3621                          31  IPEVPADENVNFSACLVNCGSNCPLKIFSQDGRIVRVETDHSG....TDQYG......
S_frigidimarina_NCIMB_400_Sfri_3128                             35  TPPVAG.ENLNWSACLVNCGSNCPVQVFSTDGVITRVESQFTT....TDKYG......
S_frigidimarina_NCIMB_400_Sfri_3684                             35  TPPVSD.ETITWSACLVNCGSNCPVKVFSSDGIITRVEADNET....TDEYG....V.
S_sediminis_HAW-EB3_Ssed_0229                                   35  QPPVGE.EQITWSSCLVNCGSNCPVKVFSRDGVITRVETDHDV....ADGDD......
S_sediminis_HAW-EB3_Ssed_0350                                   35  TPPVAG.ENLNWSACLVNCGSNCPVQVFSTDGVITRVESEFTT....TDKYG......
S_sediminis_HAW-EB3_Ssed_0359                                   37  PVLPPVGEGITWGSCATNCWQACPLKVHSKDGVITRIETEG....KATDDW..DNF..
S_sediminis_HAW-EB3_Ssed_1307                                   31  IPEVPVSEKHFWSACLGNCGSNCPLKVITVDGKITRIEPET....YGEDSFD....SP
S_sediminis_HAW-EB3_Ssed_1404                                   33  EVPVA..EKINWSSCTCNCGHSCPLKVVTRDGKIARIESDDLG....DDSWE......
S_sediminis_HAW-EB3_Ssed_2923                                   35  TPPVTEGETMNWSSCLVNCGSNCPVRVYSKDGVITRIESQFTT....EDTYG......
S_sp.KX20019_JK628_RS15600                                      35  KPPVGD.EQITWSACLVNCGSNCPVKVFSRDGVITRVETDHDI....TDGDD......
S_sp.KX20019_JK628_RS20550                                      35  TPPVVG.ENLNWSACLVNCGSNCPVQVFSTDGVITRVESQFTT....TDKYG......
Ferrimonas_sp.SCSIO_43195_J8Z22_RS00580                         37  LTNPTTGESFAWAQCQGNCWAWCPLKVITKEGKIVRVEPHTE....GDDNFN....TG
Ferrimonas_sp.SCSIO_43195_J8Z22_RS11605                         33  VTVPVE.EKLNWSACLVNCGSNCPVKVYSRDGIITRIETDYAV....EDDYG......
Pseudomonas_sp.SCT_BHD08_RS22425(idrA)                          51  ASENALGADFPHQMMMGAWASPAQHNVVSYRDQPHHVVVIADK...DATVVN......
Denitromonas_sp.IR12_I8J34_RS03780(idrA)                        59  AKDNAFNADFPHQQIFGAWASPAQHNIVNHNGRSHHVLVLPDR...DTTVVN......
```

```
S_oneidensis_MR-1_dmsA                                 82  NHQIRACARGRSLRSRTYAP.....DRLRTPMKRVG..KRGEAK.FVPISWDEAASTI
S_oneidensis_MR-1_SO_4358                              90  HHEIRPCLKGRAQKQKAYSA.....DRVKYPMKRVG..PRGSGQ.YVRISWDEAINTV
S_sp._LZH-2_JM642_14190                                82  NHQIRACARGRSLRSRTYAP.....DRLRTPMKRVG..KRGEAK.FVPISWDEAASTI
S_xiamenensis_NUITM-VS1_NUITMVS1_29540                 82  NHQIRACARGRSLRSRTYAP.....DRLRTPMKRVG..KRGEAK.FVPISWDEAASTI
S_putrefaciens_strain_FDAARGOS_681_FOB89_20615         82  NHQIRACARGRSLRSRTYAP.....DRLRTPMKRVG..KRGEAK.FVPITWDEAASTI
S_sp._MR-4_Shewmr4_3676                                84  NHQIRACARGRSLRQRTYAA.....DRLKTPMKRVG..KRGEGK.FVPITWDEAASTI
S_fidelis_ATCC-BAA-318__L884__L884_RS0114160           82  LHQVRACARGRSLRQRNYAV.....DRLKTPMKRVG..ERGKAE.FVPISWDEATSII
S_piezotolerans_WP3_SWP_RS15450                        82  IYQVRACARGRSLRQRTYAV.....DRLKSPMKRVG..KRGEGK.FVPITWDEAAKTI
S_schlegeliana_strain_JCM_JMA39_11561_RS09490          82  LHQVRACARGRSLRQRNYAV.....DRLKTPMKRVG..ERGKAE.FVPISWDEATSII
S_marisflavi_strain_EP1_CFF01_RS01175                  82  NHQIRACARGRSLKQRTYAP.....DRLKRPMKRVG..KRGEGK.FVPISWDEAASTV
S_sp.SUN_WT4_FJQ87_RS02760                             82  LHQVRACARGRSLRQRTYAP.....DRLKTPMKRVG..KRGEGK.FVPISWDEATSIV
S_sp.MBTL60-112-B2_K5Q73_RS09520                       82  LHQVRACARGRSLRQRNYAV.....DRLKTPMKRVG..ERGKAE.FVPISWDEATSII
S_sp.MBTL60_112_B1_K5Q83_RS07010                       82  LHQVRACARGRSLRQRNYAV.....DRLKTPMKRVG..ERGKAE.FVPISWDEATSII
S_enrypsychrophilus_strain_YLB-08_FM038_RS01260        82  IYQVRACARGRSLRQRTYAV.....DRLKTPMKRVG..KRGEGK.FVPISWDEAAKTI
S_enrypsychrophilus_strain_YLB-08_FM038_RS01535        87  FIHNKACAKGLMLKELVYSP.....ERLTKPLKRVG..KRGEGK.FEEISWEQAASEI
S_enrypsychrophilus_strain_YLB-08_FM038_RS23490        87  EHEIRPCLRGRSMRQKAYNA.....DRLKYPMKRVG..PRGSGE.FVRIDWEEAFSEV
S_enrypsychrophilus_strain_YLB-08_FM038_RS23525        87  DHEIRPCPRGRSMRQKVYNH.....DRLKYPMKRVG..RRGSDE.FVRISWEEAVSSV
S_sp.YLB_09_FS418_RS01245                              82  IYQVRACARGRSLRQRTYAV.....DRLKTPMKRVG..KRGEGK.FVPISWDEAAKTI
S_sp.YLB_09_FS418_RS01520                              87  FIHNKACAKGLMLKELVYSP.....ERLTKPLKRVG..KRGEGK.FEEISWEQAASEI
S_sp.YLB_09_FS418_RS02865                              82  IYQVRACARGRSLRQRTYAV.....DRLKTPMKRVG..KRGEGK.FVPISWDEAAKTI
S_sp.YLB_09_FS418_RS03140                              87  FIHNKACAKGLMLKELVYSP.....ERLTKPLKRVG..KRGEGK.FEEISWEQAASEI
S_sp.YLB_09_FS418_RS25020                              87  EHEIRPCLRGRSMRQKAYNA.....DRLKYPMKRVG..PRGSGE.FVRIDWEEAFSEV
S_sp.YLB_09_FS418_RS25055                              87  DHEIRPCPRGRSMRQKVYNH.....DRLKYPMKRVG..RRGSDE.FVRISWEEAVSSV
S_sp._WPAGA9_IGB07_RS19235                             81  MHQVRACARGRSLRQRTYAL.....DRLKSPMKRVG..ARGEGK.FVPITWDEAAETI
S_sp._ARC9_LZ_GUY17_RS19100                            84  NHQARACPRGRSLRQRTYAT.....DRLRSPMKRVG..KRGEAK.FVPISWEQAYSEI
S_psychromarinicola_strain_M2_EGC80_RS00660            82  DHDVRACPRGRSLRKRVYAP.....DRLKYPMKRVG..PRGSGQ.FERISWDEALDLV
S_psychromarinicola_strain_M2_EGC80_RS06745            84  NHQARACPRGRSLRQRTYAT.....DRLRSPMKRVG..KRGEAK.FVPISWEQAYSEI
S_psychromarinicola_strain_M2_EGC80_RS11440            85  QQR..PCARGHSAKQKIYNP.....DRILHPLKRIG..ERGSGQ.FEQISWDQAFSEI
S_sp.Actino-trap-3_CXF80_RS10115                       82  DHDVRACPRGRSLRKRVYAP.....DRLKYPMKRVG..PRGSGQ.FERISWDEALDLV
S_sp.Actino-trap-3_CXF80_RS15635                       84  NHQARACPRGRSLRQRTYAT.....DRLRSPMKRVG..KRGEAK.FVPISWEQAYSEI
S_livingstonensis_strain_LMG_19866_EGC82_RS20515       84  NHQARACPRGRSLRQRTYAT.....DRLRSPMKRVG..KRGEAK.FVPISWEQAYSEI
Ferrimonas_lipolytica_strain_S7_HER31_RS14690          81  EHQSRACARGRSFKRKVYAA.....DRLKVPMKRVG..KRGEGN.FEEISWEEAYDTI
Ferrimonas_lipolytica_strain_S7_HER31_RS15965          79  NHQVRACLRGRSLRQRNYAP.....DRLKTPLRRVAGTKRGAGQ.WEQISWETAFSEI
Ferrimonas_lipolytica_strain_S7_HER31_RS04980          82  QHR..PCAKGLAAFQKIYSP.....DRIQNPLKRVG..PRGYKDSYVEISWDQAFDEI
S_sp.ISTPL2_CCLCJOKE_1_HUB64_RS13995                   82  NHQIRACARGRSLRSRTYAP.....DRLRTPMKRVG..KRGEAK.FVPITWDEAASTI
S_japonica_strain_KCTC_22435_SJ2017_RS00815            81  MHQVRACARGRSLRQRTYAL.....DRLKSPMKRVG..ERGEGK.FVPITWDEAAETI
S_sp.8A_M2897_RS06500                                  82  NHQIRACARGRSLRSRTYAP.....DRLRTPMKRVG..KRGEAK.FVPISWDEAASTI
S_woodyi_ATCC_51908_Swoo_0233                          82  IYQVRACARGRSLRQRTYAV.....DRLKTPMKRVG..KRGEGK.FVPISWDEAAKTI
Ferrimonas_balerica_DSM_9799_Fbal_2476                 79  SRQIRACPRGRSLRQRVYSD.....ERILYPMKRVG..PRGSGE.FVRISWDDALTEI
Ferrimonas_balerica_DSM_9799_Fbal_3621                 79  DHQVRACLRGRSIKQRTYAA.....DRLKAPMKRKAGTKRGAGM.YEEITWEQAFAEI
S_frigidimarina_NCIMB_400_Sfri_3128                    82  DHDVRACPRGRSLRKRVYAP.....DRLKYPMKRVG..PRGSGQ.FERISWDEALDLV
S_frigidimarina_NCIMB_400_Sfri_3684                    83  NHQIRACARGRSLRQRTYAA.....DRLKTPMKRVG..KRGEGK.FVPISWDEAASTI
S_sediminis_HAW-EB3_Ssed_0229                          82  VYQVRACARGRSLRQRTYAV.....DRLKTPMKRVG..KRGEGK.FVPISWDEAAKTI
S_sediminis_HAW-EB3_Ssed_0350                          82  DHDVRACLRGRSLRKRVYAP.....DRLKYPMKRVG..PRGSGQ.FERISWDEALDLV
S_sediminis_HAW-EB3_Ssed_0359                          87  DHEIRPCPRGRSMRQKVYNH.....DRLKYPMKRVG..RRGSDE.FVRISWEEAVSSV
S_sediminis_HAW-EB3_Ssed_1307                          81  HIR..PCLRGHANRARVYNP.....DRLMYPMKRIANAKRGEGK.FERISWEQATSEI
S_sediminis_HAW-EB3_Ssed_1404                          79  SHQSRACAKGRSTKKKVYAA.....DRLKVPMKRVG..KRGEGN.FREISWEEAFDTI
S_sediminis_HAW-EB3_Ssed_2923                          83  NHDVRACLRGRSLRKRVYAP.....ERLKYPMKRVG..PRGSGQ.FERISWDEALNTV
S_sp.KX20019_JK628_RS15600                             82  IYQVRACARGRSLRQRTYAV.....DRLKSPMKRVG..KRGEGK.FVPITWDEAAKTI
S_sp.KX20019_JK628_RS20550                             82  DHDVRACPRGRSLRKRVYAP.....DRLKYPMKRVG..PRGSGQ.FERISWDEALDLV
Ferrimonas_sp.SCSIO_43195_J8Z22_RS00580                87  FIHNKACAKGLMLKELVYSP.....QRLKTPLKRTG..PRGSGQ.FKEISWDQAAREV
Ferrimonas_sp.SCSIO_43195_J8Z22_RS11605                80  NHQVRACLRGRSLRKRVYAP.....DRLKYPMKRVG..PRGSGQ.FERISWDEALNTV
Pseudomonas_sp.SCT_BHD08_RS22425(idrA)                100  .PGGNHSIRGGTLAEKCYNPSNRTRERLQHPMIRVN......GK.LTPVSWDLATEVM
Denitromonas_sp.IR12_I8J34_RS03780(idrA)              108  .PGGNHSIRGGTLAQKCYNPSNRTSERLLYPMIRVR......GT.LMPVSWDLATEVM
```

```
                                                                         .                       .               .                         .
S_oneidensis_MR-1_dmsA                                    132  ASEIQRIAS...SYGNKSILKHYGTGAYYGFASS.ACFDR.....VLN.........
S_oneidensis_MR-1_SO_4358                                 140  AQELSTKIT...TYGAQSIYLPVATGDYDYQVDGMYAAMM.....FLHEITKKKIPL
S_sp._LZH-2_JM642_14190                                   132  ASEIQRIAS...SYGNKSILKHYGTGAYYGFASS.ACFDR.....VLN.........
S_xiamenensis_NUITM-VS1_NUITMVS1_29540                    132  ASEIQRIAS...SYGNKSILKHYGTGAYYGFASS.ACFDR.....VLN.........
S_putrefaciens_strain_FDAARGOS_681_FOB89_20615            132  ASEIQRIAS...SYGNKSILKHYGTGAYYGFASS.ACFDR.....VLN.........
S_sp._MR-4_Shewmr4_3676                                   134  ASELQRITA...EYTNRSILKLYGSGAYYGFSSS.NCFNR.....LLN.........
S_fidelis_ATCC-BAA-318__L884__L884_RS0114160              132  AEKLSAVYAQ..ENGAKSVFRSYGSGAYYGFASN.ACFNR.....LFN.........
S_piezotolerans_WP3_SWP_RS15450                           132  GNKLTSVIA...EHGNKAIFRSYGSGAYYGFASN.ACFNR.....LLN.........
S_schlegeliana_strain_JCM_JMA39_11561_RS09490             132  AEKLSAVYAQ..ENGAKSVFRSYGSGAYYGFASN.ACFNR.....LFN.........
S_marisflavi_strain_EP1_CFF01_RS01175                     132  ASELARITE...QYGNRSILKLYGSGAYYGFASS.NCFNR.....LLN.........
S_sp.SUN_WT4_FJQ87_RS02760                                132  AENLQRVIN...QYGNQAVFRSYGSGAYYGFASN.ANFNR.....LFN.........
S_sp.MBTL60-112-B2_K5Q73_RS09520                          132  AEKLAAVYAQ..ENGAKSVFRSYGSGAYYGFASN.ACFNR.....LFN.........
S_sp.MBTL60_112_B1_K5Q83_RS07010                          132  AEKLAAVYAQ..ENGAKSVFRSYGSGAYYGFASN.ACFNR.....LFN.........
S_enrypsychrophilus_strain_YLB-08_FM038_RS01260           132  GTKLTSVIA...EHGNKAIFRSYGSGAYYGFASN.ACFNR.....LFN.........
S_enrypsychrophilus_strain_YLB-08_FM038_RS01535           137  AQSLRSTID...SCGNRAVMLGNGSGAAHSSYPIMGPHVR.....LMN.........
S_enrypsychrophilus_strain_YLB-08_FM038_RS23490           137  ARMIQETTS...NYGKNSIYVPLGGGSHDLSAGAMIMGLR.....LLS.........
S_enrypsychrophilus_strain_YLB-08_FM038_RS23525           137  AAAMQKTYA...EYGKNAIFMPLGGGSHDLNTGVTQSSVR.....LLN.........
S_sp.YLB_09_FS418_RS01245                                 132  GTKLTSVIA...EHGNKAIFRSYGSGAYYGFASN.ACFNR.....LFN.........
S_sp.YLB_09_FS418_RS01520                                 137  AQSLRSTID...SCGNRAVMLGNGSGAAHSSYPIMGPHVR.....LMN.........
S_sp.YLB_09_FS418_RS02865                                 132  GTKLTSVIA...EHGNKAIFRSYGSGAYYGFASN.ACFNR.....LFN.........
S_sp.YLB_09_FS418_RS03140                                 137  AQSLRSTID...SCGNRAVMLGNGSGAAHSSYPIMGPHVR.....LMN.........
S_sp.YLB_09_FS418_RS25020                                 137  ARMIQETTS...NYGKNSIYVPLGGGSHDLSAGAMIMGLR.....LLS.........
S_sp.YLB_09_FS418_RS25055                                 137  AAAMQKTYA...EYGKNAIFMPLGGGSHDLNTGVTQSSVR.....LLN.........
S_sp._WPAGA9_IGB07_RS19235                                131  GSKLNSVIA...EHGNKAIFRSYGSGAYYGFASN.GCFNR.....LFN.........
S_sp._ARC9_LZ_GUY17_RS19100                               134  GTKLQAIKD...NYGPESIYMHYGTGAYYGFSSNTATWGR.....LLN.........
S_psychromarinicola_strain_M2_EGC80_RS00660               132  AEKLQDTIY...QYGNESIHFTYNSGARYHFSGK.QCLYR.....LMN.........
S_psychromarinicola_strain_M2_EGC80_RS06745               134  GSKLQAIKD...NYGPESIYMHYGTGAYYGFSSNTATWGR.....LLN.........
S_psychromarinicola_strain_M2_EGC80_RS11440               133  GVKLKNIYD...TYGSRSVFNLAGS.SLGAIHSPT.PINN.....LLN.........
S_sp.Actino-trap-3_CXF80_RS10115                          132  AEKLQDTIY...QYGNESIHFTYNSGARYHFSGK.QCLYR.....LMN.........
S_sp.Actino-trap-3_CXF80_RS15635                          134  GSKLQAIKD...NYGPESIYMHYGTGAYYGFSSNTATWGR.....LLN.........
S_livingstonensis_strain_LMG_19866_EGC82_RS20515          134  GTKLQAIKD...NYGPESIYMHYGTGAYYGFSSNTATWGR.....LLN.........
Ferrimonas_lipolytica_strain_S7_HER31_RS14690             131  ADNLKRIID...TYGNESVYCQYATGRLYSFFSGGHWINAGQWGGRLLN.........
Ferrimonas_lipolytica_strain_S7_HER31_RS15965             131  GTKLAQLKA...DHGPRSIYHHYVSGAYYSFASG.NCIRR.....ALD.........
Ferrimonas_lipolytica_strain_S7_HER31_RS04980             131  AEKLTAIYD...NYGPRAVLNGGGS.NYGGSHSGF.WVTN.....LLS.........
S_sp.ISTPL2_CCLCJOKE_1_HUB64_RS13995                      132  ASEIQRIAS...SYGNKSILKHYGTGAYYGFASS.ACFDR.....VLN.........
S_japonica_strain_KCTC_22435_SJ2017_RS00815               131  GSKLNSVIA...EHGNKAIFRSYGSGAYYGFASN.GCFNR.....LFN.........
S_sp.8A_M2897_RS06500                                     132  ASEIQRIAS...SYGNKSILKHYGTGAYYGFASS.ACFDR.....VLN.........
S_woodyi_ATCC_51908_Swoo_0233                             132  GTKLSSVIN...EHGNKAIFRSYGSGAYYGFASN.ACFNR.....LFN.........
Ferrimonas_balerica_DSM_9799_Fbal_2476                    129  HQRLEAIYQDSSQGGPESVFFPVSTGTSGMKTSGTGIWKR.....LFH.........
Ferrimonas_balerica_DSM_9799_Fbal_3621                    131  GQKVMDLQG...TYGPRSIYHHYGSGAYYGFASS.SCVQR.....ALR.........
S_frigidimarina_NCIMB_400_Sfri_3128                       132  AEKLQGTID...QYGNESIHFTYNSGARYHFSGK.QCLYR.....LMN.........
S_frigidimarina_NCIMB_400_Sfri_3684                       133  ASELQRIAN...EYSNKSILKIYGTGAYYGFSSS.SCFNR.....VLN.........
S_sediminis_HAW-EB3_Ssed_0229                             132  GTKLTSVIA...EHGNKAIFRSYGSGAYYGFASN.ACFNR.....LFN.........
S_sediminis_HAW-EB3_Ssed_0350                             132  AEKLQGTID...QYGNESIHFTYNSGARYHFSGK.QCLYR.....LMN.........
S_sediminis_HAW-EB3_Ssed_0359                             137  ASAMEQTYS...QYGKNAIFMPLGGGSHDLNTGVTQSSVR.....LLN.........
S_sediminis_HAW-EB3_Ssed_1307                             131  GMKLKETYD...QYGPRAVYQNRGSGAFYDINGKY.AWGR.....LLN.........
S_sediminis_HAW-EB3_Ssed_1404                             129  AAQLRRVID...TYGNESVYCQYASGRLYSFFSGGHWAYGGLWGAKLLN.........
S_sediminis_HAW-EB3_Ssed_2923                             133  AERLQGIID...QYGNESIFDMTGTGTFGHFKSL.AMVHR.....LLN.........
S_sp.KX20019_JK628_RS15600                                132  ATKLTSVIA...EHGNKAIFRSYGSGAYYGFASN.GCFNR.....LLN.........
S_sp.KX20019_JK628_RS20550                                132  AEKLQGTID...QYGNESIHFTYNSGARYHFSGK.QCLYR.....LMN.........
Ferrimonas_sp.SCSIO_43195_J8Z22_RS00580                   137  AQSIQQTIE...LYGNRALMLGNGSGSAHSSYPLMGPHVR.....LLN.........
Ferrimonas_sp.SCSIO_43195_J8Z22_RS11605                   130  ASELGRLRG...QYGPECTHFTYHSGAYYSFAGF.NCLAR.....LMN.........
Pseudomonas_sp.SCT_BHD08_RS22425(idrA)                    150  ADISQYIIA...KYGEHAWAVKSHSYQYFENVYAITKLAMT................
Denitromonas_sp.IR12_I8J34_RS03780(idrA)                  158  ADISQYILA...KYGEHAWAMKTYSYQYFENTYAITKLGMT................
```

```
                       .          .                    .
S_oneidensis_MR-1_dmsA 171 ...........LNGG...FLKIYGNYSWAQQNEAAAATGFA...........STSGSS
S_oneidensis_MR-1_SO_4358 189 YIPEMDFTMELDASG...YLMYHDSYSAAQMKDAVKSTFGWDD........LWGTGNT
S_sp._LZH-2_JM642_14190 171 ...........LNGG...FLKIYGNYSWAQQNEAAAATGFG...........STSGSS
S_xiamenensis_NUITM-VS1_NUITMVS1_29540 171 ...........LNGG...FLKIYGNYSWAQQNEAAAATGFG...........STSGSS
S_putrefaciens_strain_FDAARGOS_681_FOB89_20615 171 ...........LNGG...FLKIYGNYSWAQQNEAAAATGFG...........STSGSS
S_sp._MR-4_Shewmr4_3676 173 ...........LNGG...FLNIYGNYSWAQQIEAAAATWGGS..........STNGSS
S_fidelis_ATCC-BAA-318__L884__L884_RS0114160 172 ...........LNGG...CLNAYGNYSWAQQMEAAKHTFGTG..........STAGSS
S_piezotolerans_WP3_SWP_RS15450 171 ...........LNGG...CLSYYGNYSWAQQQEAAAHTFGTG..........STKGSS
S_schlegeliana_strain_JCM_JMA39_11561_RS09490 172 ...........LNGG...CLNAYGNYSWAQQMEAAKHTFGTG..........STAGSS
S_marisflavi_strain_EP1_CFF01_RS01175 171 ...........LNGG...FLNIYGNYSWAQQIDAAYATWAGS..........STNGSS
S_sp.SUN_WT4_FJQ87_RS02760 171 ...........LVGG...CLNAYGNYSWAQQMEAAQHTFGTG..........STMGSS
S_sp.MBTL60-112-B2_K5Q73_RS09520 172 ...........LNGG...CLNAYGNYSWAQQMEAAKHTFGTG..........STSGSS
S_sp.MBTL60_112_B1_K5Q83_RS07010 172 ...........LNGG...CLNAYGNYSWAQQMEAAKHTFGTG..........STSGSS
S_enrypsychrophilus_strain_YLB-08_FM038_RS01260 171 ...........LNGG...CLNAYGNYSWAQQMEAAKHTFGTG..........STSGSS
S_enrypsychrophilus_strain_YLB-08_FM038_RS01535 177 ...........LLGG...FQKTYGYYSACMKEQAIPYTFGPSTKNL..AYGQGTGSSS
S_enrypsychrophilus_strain_YLB-08_FM038_RS23490 177 ...........TWEG...CLFFHNDYSASQYREGSASMYGWLHYKVNDTVGFWNTGNR
S_enrypsychrophilus_strain_YLB-08_FM038_RS23525 177 ...........MAGG...CLFFHNDYSASQYREGSAGMYGYLASNDGDTVGFWNTGSS
S_sp.YLB_09_FS418_RS01245 171 ...........LNGG...CLNAYGNYSWAQQMEAAKHTFGTG..........STSGSS
S_sp.YLB_09_FS418_RS01520 177 ...........LLGG...FQKTYGYYSACMKEQAIPYTFGPSTKNL..AYGQGTGSSS
S_sp.YLB_09_FS418_RS02865 171 ...........LNGG...CLNAYGNYSWAQQMEAAKHTFGTG..........STSGSS
S_sp.YLB_09_FS418_RS03140 177 ...........LLGG...FQKTYGYYSACMKEQAIPYTFGPSTKNL..AYGQGTGSSS
S_sp.YLB_09_FS418_RS25020 177 ...........TWEG...CLFFHNDYSASQYREGSASMYGWLHYKVNDTVGFWNTGNR
S_sp.YLB_09_FS418_RS25055 177 ...........MAGG...CLFFHNDYSASQYREGSAGMYGYLASNDGDTVGFWNTGSS
S_sp._WPAGA9_IGB07_RS19235 170 ...........LVGG...CLQYFGNYSWAQQTEAAKHTFGTG..........STSGSA
S_sp._ARC9_LZ_GUY17_RS19100 174 ...........LNGG...FLDHHWDYSWAGVYPACMATYGDQW........DSIGGSS
S_psychromarinicola_strain_M2_EGC80_RS00660 171 ...........LKGG...YLNAYGDYSWSQIYEAAGQTYGSAG........PGWQGSS
S_psychromarinicola_strain_M2_EGC80_RS06745 174 ...........LNGG...FLDHHWDYSWAGVYPACMATYGDQW........DSIGGSS
S_psychromarinicola_strain_M2_EGC80_RS11440 171 ...........VMGG...YTGLFSSMSFSQVEKIMPYMYG............TRPYSS
S_sp.Actino-trap-3_CXF80_RS10115 171 ...........LKGG...YLNAYGDYSWSQIYEAAGQTYGSAG........PGWQGSS
S_sp.Actino-trap-3_CXF80_RS15635 174 ...........LNGG...FLDHHWDYSWAGVYPACMATYGDQW........DSIGGSS
S_livingstonensis_strain_LMG_19866_EGC82_RS20515 174 ...........LNGG...FLDHHWDYSWAGVYPACIATYGDQW........DSIGGSS
Ferrimonas_lipolytica_strain_S7_HER31_RS14690 177 ...........LLGG...YTRQHNTYSSGQLANAGSLMFGTT............SSSS
Ferrimonas_lipolytica_strain_S7_HER31_RS15965 170 ...........CAGGERGFLAYYSNYSWAALTETAGATFGHG..........ATSGTR
Ferrimonas_lipolytica_strain_S7_HER31_RS04980 169 ...........AAGG...CITTFGAMSFAQVARIMGHVFGG........FDAERDFSS
S_sp.ISTPL2_CCLCJOKE_1_HUB64_RS13995 171 ...........LNGG...FLKIYGNYSWAQQNEAAAATGFG...........STSGSS
S_japonica_strain_KCTC_22435_SJ2017_RS00815 170 ...........LVGG...CLQYFGNYSWAQQTEAAKHTFGTG..........STSGSA
S_sp.8A_M2897_RS06500 171 ...........LNGG...FLKIYGNYSWAQQNEAAAATGFG...........STSGSS
S_woodyi_ATCC_51908_Swoo_0233 171 ...........LNGG...CLNAYGNYSWAQQMEAAKHTFGTG..........STTGSS
Ferrimonas_balerica_DSM_9799_Fbal_2476 172 ...........QLGG...YLGTRGSYSTHQVDTATQYTYGIGSVI......LNGLGSS
Ferrimonas_balerica_DSM_9799_Fbal_3621 170 ...........LSGG...HLNYYGNYSWAQLNVASPATFGTG..........GTSGTR
S_frigidimarina_NCIMB_400_Sfri_3128 171 ...........LKGG...YLNAYGDYSWSQIYEAAGQTYGSAG........PGWQGSS
S_frigidimarina_NCIMB_400_Sfri_3684 172 ...........LNGG...FLNIYGNYSWAQQTEAAAATGFS...........STSGSN
S_sediminis_HAW-EB3_Ssed_0229 171 ...........LNGG...CLNAYGNYSWAQQMEAAKHTFGTG..........STSGSS
S_sediminis_HAW-EB3_Ssed_0350 171 ...........LKGG...YLNAYGDYSWSQIYEAAGQTYGSAG........PGWQGSS
S_sediminis_HAW-EB3_Ssed_0359 177 ...........MAGG...CLFFHNDYSAAQYREGSAGMYGYLASNAGDTVGFWNTGSS
S_sediminis_HAW-EB3_Ssed_1307 170 ...........CMGG...GLKHYGSYSSSMVRTIMPYIFG............SRKSST
S_sediminis_HAW-EB3_Ssed_1404 175 ...........ILGG...YLGQYNTYSSGQASNAGQYTFGGR............PASG
S_sediminis_HAW-EB3_Ssed_2923 172 ...........AMGG...SLKRYGVYSYAQLFEASRYTYGTPF........VGLQGST
S_sp.KX20019_JK628_RS15600 171 ...........LNGG...CLSYYGNYSWAQQQEAAAHTFGTG..........STKGSS
S_sp.KX20019_JK628_RS20550 171 ...........LKGG...YLNAYGDYSWSQIYEAAGQTYGSAG........PGWQGSS
Ferrimonas_sp.SCSIO_43195_J8Z22_RS00580 177 ...........MLGG...FQKTYGYYSASMKEQAIPYTFGDGTKNL..VYGQGTGSSS
Ferrimonas_sp.SCSIO_43195_J8Z22_RS11605 169 ...........ITGG...ALGAYGDYSWAQLYQAANYTYGDAN........HGWLGSS
Pseudomonas_sp.SCT_BHD08_RS22425(idrA) 188 ...........SIG........TPAFAWHDKCSATNDATGLDDAG......IDSFASS
Denitromonas_sp.IR12_I8J34_RS03780(idrA) 196 ...........SIG........TPAFAWHDKASATNDATGLDDAG......VNSFASS
```

**S_oneidensis_MR-1_dmsA**	**204**	LTALYD.SDLFLGIGYNPSEMRQSGSGEGYDFLKALE...ANA.......NLEVIMV
**S_oneidensis_MR-1_SO_4358**	**236**	LLDVKNGTDLMICFGYNPMESRMGGAGSAYDYTNLKRQF...........GFKTIYI
**S_sp._LZH-2_JM642_14190**	**204**	LTALYD.SDLFLGIGYNPSEMRQSGSGEGYDFLKALE...ANA.......NLEVIMV
**S_xiamenensis_NUITM-VS1_NUITMVS1_29540**	**204**	LTALYD.SDLFLGIGYNPSEMRQSGSGEGYDFLKALE...ANA.......NLEVIMV
**S_putrefaciens_strain_FDAARGOS_681_FOB89_20615**	**204**	LTALYD.SDLFLGIGYNPSEMRQSGSGEGYDFLKALE...ANS.......NLEVIMV
**S_sp._MR-4_Shewmr4_3676**	**207**	MVCVAD.SDLFLGIGYNPSEMRQSGSGEGYDYLQAIQ...NNP.......SIEVIMV
**S_fidelis_ATCC-BAA-318__L884__L884_RS0114160**	**206**	TITIED.SDFFLGVAYNPSEIRQSGSGEGYDYLQALQ...RNN.......GLEVVMI
**S_piezotolerans_WP3_SWP_RS15450**	**205**	TVTLSN.SDFLLGIAYNPSEIRQSGSGEGYDYLKALQ...KSN.......GLKVTMI
**S_schlegeliana_strain_JCM_JMA39_11561_RS09490**	**206**	TITIED.SDFFLGVAYNPSEIRQSGSGEGYDYLQALQ...RNN.......GLEVVMI
**S_marisflavi_strain_EP1_CFF01_RS01175**	**205**	VVCLED.SDLFIGIGYNPSEMRQSGSGEGYDFLQALK...NNP.......SLEVIMI
**S_sp.SUN_WT4_FJQ87_RS02760**	**205**	TSTIAD.SDFFLGVAYNPSEIRQSGSGEGYDFLQALQ...KNN.......DLKVVMI
**S_sp.MBTL60-112-B2_K5Q73_RS09520**	**206**	TITIED.SDFFLGVAYNPSEIRQSGSGEGYDYLQALQ...RNN.......GLEVVMI
**S_sp.MBTL60_112_B1_K5Q83_RS07010**	**206**	TITIED.SDFFLGVAYNPSEIRQSGSGEGYDYLQALQ...RNN.......GLEVVMI
**S_enrypsychrophilus_strain_YLB-08_FM038_RS01260**	**205**	TVTLAN.SDFLLGVAYNPSEIRQSGSGEGYDYLKALQ...KSN.......GLKVVMI
**S_enrypsychrophilus_strain_YLB-08_FM038_RS01535**	**219**	VTEMKH.SDYILSFGYNPLEMFMTGGGEGHHFAQTLESN...........DIKFVYV
**S_enrypsychrophilus_strain_YLB-08_FM038_RS23490**	**221**	LRDLEH.SDLMLCFGYNPMEARMGGAGSGYENAKLKQKN...........SYKTYYI
**S_enrypsychrophilus_strain_YLB-08_FM038_RS23525**	**221**	LRSLEN.SDLMVCFGYNPMEARLGGAGSGYEYLNVKKKN...........NFKTYYI
**S_sp.YLB_09_FS418_RS01245**	**205**	TVTLAN.SDFLLGVAYNPSEIRQSGSGEGYDYLKALQ...KSN.......GLKVVMI
**S_sp.YLB_09_FS418_RS01520**	**219**	VTEMKH.SDYILSFGYNPLEMFMTGGGEGHHFAQTLESN...........DIKFVYV
**S_sp.YLB_09_FS418_RS02865**	**205**	TVTLAN.SDFLLGVAYNPSEIRQSGSGEGYDYLKALQ...KSN.......GLKVVMI
**S_sp.YLB_09_FS418_RS03140**	**219**	VTEMKH.SDYILSFGYNPLEMFMTGGGEGHHFAQTLESN...........DIKFVYV
**S_sp.YLB_09_FS418_RS25020**	**221**	LRDLEH.SDLMLCFGYNPMEARMGGAGSGYENAKLKQKN...........SYKTYYI
**S_sp.YLB_09_FS418_RS25055**	**221**	LRSLEN.SDLMVCFGYNPMEARLGGAGSGYEYLNVKKKN...........NFKTYYI
**S_sp._WPAGA9_IGB07_RS19235**	**204**	TVTLES.SDFLLGFAYNPSEIRQSGSGEGYDYLQALQ...RAN.......GLKVTMI
**S_sp._ARC9_LZ_GUY17_RS19100**	**210**	LSEIVN.SDLFIGFGFNPNETRMSGSGEGYDFVKSLE...HNNK......SIKVWMI
**S_psychromarinicola_strain_M2_EGC80_RS00660**	**207**	VSEMQN.SDLVLMVGYNPSEIRMSGSGEAYDFLVQKQ...KHN........FKTILI
**S_psychromarinicola_strain_M2_EGC80_RS06745**	**210**	LSEIVN.SDLFVGFGFNPNETRMSGSGEGYDFVKSLE...NNKK......NIKVWMV
**S_psychromarinicola_strain_M2_EGC80_RS11440**	**203**	IVESKF.SDLYLFFGFNPLETGASGAGYGFEWQHVKG.............DTPVICF
**S_sp.Actino-trap-3_CXF80_RS10115**	**207**	VSEMQN.SDLVLMVGYNPSEIRMSGSGEAYDFLVQKQ...KHN........FKTILI
**S_sp.Actino-trap-3_CXF80_RS15635**	**210**	LSEIVN.SDLFVGFGFNPNETRMSGSGEGYDFVKSLE...NNKK......NIKVWMV
**S_livingstonensis_strain_LMG_19866_EGC82_RS20515**	**210**	LSEIVN.SDLFVGFGFNPNETRMSGSGEGYDFVKSLE...HNNK......SIKVWMI
**Ferrimonas_lipolytica_strain_S7_HER31_RS14690**	**209**	YAEMGK.SDFILTFGFNPAEMRASGAGGTYDYSVFTQ.............NVETVVV
**Ferrimonas_lipolytica_strain_S7_HER31_RS15965**	**207**	HSHIRD.ADFFLGIGFNPFEMRMSGSGEQIDFMKAVEERRANGQ......KFKAIMI
**Ferrimonas_lipolytica_strain_S7_HER31_RS04980**	**205**	VVEMQN.SDFILSFGFNPLETAANGSGMGFEYNHVVD.............GKPLIIF
**S_sp.ISTPL2_CCLCJOKE_1_HUB64_RS13995**	**204**	LTALYD.SDLFLGIGYNPSEMRQSGSGEGYDFLKALE...ANS.......NLEVIMV
**S_japonica_strain_KCTC_22435_SJ2017_RS00815**	**204**	TVTLES.SDFLLGFAYNPSEIRQSGSGEGYDYLQALQ...RAN.......GLKVTMI
**S_sp.8A_M2897_RS06500**	**204**	LTALYD.SDLFLGIGYNPSEMRQSGSGEGYDFLKALE...ANA.......NLEVIMV
**S_woodyi_ATCC_51908_Swoo_0233**	**205**	TVTLSN.SDFLLGVAYNPSEIRQSGSGEGYDYLKALQ...KSN.......GLKVVMI
**Ferrimonas_balerica_DSM_9799_Fbal_2476**	**210**	TREIER.SDLVLCFGYNPQETFMSGGGVSYEWSEALH...HSN........AEVIMI
**Ferrimonas_balerica_DSM_9799_Fbal_3621**	**204**	LSEIRN.SDLVVGFGFNPFEIRMSGSGEQIDFLHAIETRRQTG.......DLEVIIV
**S_frigidimarina_NCIMB_400_Sfri_3128**	**207**	VSEMQN.SDLVLMVGYNPSEIRMSGSGEAYDFLVQKQ...KHN........FKTILI
**S_frigidimarina_NCIMB_400_Sfri_3684**	**205**	LLALND.SDLFIGLGYNPSEMRQSGSGEGYDFLNALQ...KNN.......GLEVIMI
**S_sediminis_HAW-EB3_Ssed_0229**	**205**	TVTLAN.SDFLLGVAYNPSEIRQSGSGEGYDYLKALQ...KSN.......GLKVVMI
**S_sediminis_HAW-EB3_Ssed_0350**	**207**	VSEMQN.SDLVLMVGYNPSEIRMSGSGEAYDFLMQKQ...KNK........FKTILI
**S_sediminis_HAW-EB3_Ssed_0359**	**221**	LRSLED.SDLMVCFGYNPMEARLGGAGSGYEYLNVKKKN...........NFKTYYI
**S_sediminis_HAW-EB3_Ssed_1307**	**202**	PREMKY.SDLLVFFGYNPLETRMSGSGEGFEVQTVTE.............GKKVIHI
**S_sediminis_HAW-EB3_Ssed_1404**	**207**	YAELRN.SDLYLSFGHNPAETEMSGAGGSYALSVVAK.............GVETYVV
**S_sediminis_HAW-EB3_Ssed_2923**	**208**	YSEMQH.SDLVLFVGHNPSELRMSGSGQGYDFLNQKQ...TNN........FKTIII
**S_sp.KX20019_JK628_RS15600**	**205**	TVTLAN.SDFLLGFAYNPSEIRQSGSGEGYDYLKALQ...KAN.......GLKVTMI
**S_sp.KX20019_JK628_RS20550**	**207**	VSEMQN.SDLVLMVGYNPSEIRMSGSGEAYDFLVQKQ...QHK........FKTILI
**Ferrimonas_sp.SCSIO_43195_J8Z22_RS00580**	**219**	VTEMMH.SDYILSFGYNPLEIFMTGGGEGHHFSQTLERN...........DARFVYV
**Ferrimonas_sp.SCSIO_43195_J8Z22_RS11605**	**205**	NAEMRK.SDLVLLIGHNPAEMRMSGSGESYDYLNIIQ...DKQKQRAQDGTQFKTIII
**Pseudomonas_sp.SCT_BHD08_RS22425(idrA)**	**221**	YEDWAD.CEVAFLSGVDPYETKTT....LFTSHMMPG.............DKKFVFV
**Denitromonas_sp.IR12_I8J34_RS03780(idrA)**	**229**	DQDWAD.CEVAFLSGVDPYETKTT....LFTQWMMPG.............DKKFIFV

```
S_oneidensis_MR-1_dmsA                                  250  DPRYTDSMAGKESQ....WLPIRPGTDAAFAEAIAYEMIS......SGWVDTHSKAF
S_oneidensis_MR-1_SO_4358                               282  EPRYTDTMVVCDDQ....WVPIRPGTDAALAEAIAYVWIAEMD.......EAPLDMAF
S_sp._LZH-2_JM642_14190                                 250  DPRYTDSMAGKESQ....WLPIRPGTDAAFAEAIAYEMIS......SGWVDTHSKAF
S_xiamenensis_NUITM-VS1_NUITMVS1_29540                  250  DPRYTDSMAGKESQ....WLPIRPGTDAAFAEAIAYEMIS......SGWVDTHSKAF
S_putrefaciens_strain_FDAARGOS_681_FOB89_20615          250  DPRYTDSMAGKESQ....WLPIRPGTDAAFAEAIAYEMIS......SGWVETHSKAF
S_sp._MR-4_Shewmr4_3676                                 253  DPRYTDSMAGKEDH....WYPIRPGTDAAFAEAIAYEMIS......SGWVDANSKAF
S_fidelis_ATCC-BAA-318__L884__L884_RS0114160            252  DPRYTDSMLGKESS....WLPIRPGTDAAFAEAVAHEMIN......HANFDSQSLPF
S_piezotolerans_WP3_SWP_RS15450                         251  DPRYTDSMLGKEST....WLPIRPGTDAAMAEAIAYQMIS......SGWVDTNSLAF
S_schlegeliana_strain_JCM_JMA39_11561_RS09490           252  DPRYTDSMLGKESS....WLPIRPGTDAAFAEAVAYEMIN......HANFAAQSLPF
S_marisflavi_strain_EP1_CFF01_RS01175                   251  DPRYTDSMAGKEDK....WLPIRPGTDAVLAEAIAYEMIN......SGWVEQNSKAF
S_sp.SUN_WT4_FJQ87_RS02760                              251  DPRYTDSMLGKESM....WLPIRPGTDAAFAEAVAYQMIS......TDWVAKNSLDF
S_sp.MBTL60-112-B2_K5Q73_RS09520                        252  DPRYTDSMLGKESS....WLPIRPGTDAAFAEAVAYVMIN......HANFAGQSLPF
S_sp.MBTL60_112_B1_K5Q83_RS07010                        252  DPRYTDSMLGKESS....WLPIRPGTDAAFAEAVAYVMIN......HANFAGQSLPF
S_enrypsychrophilus_strain_YLB-08_FM038_RS01260         251  DPRYTDSMLGKESK....WLPIRPGTDAAFAEAIAYQMIS......SGWVDNNSLNF
S_enrypsychrophilus_strain_YLB-08_FM038_RS01535         264  DPRYTDTCLGREDD....WLGIRPGTDGALIEAIFHQVLV........AYGYDSETN
S_enrypsychrophilus_strain_YLB-08_FM038_RS23490         266  DPRFTDTAVAAHDE....WVPIRPGTDAALCEGIAHHLISND.........LHEKEF
S_enrypsychrophilus_strain_YLB-08_FM038_RS23525         266  DPRFTDTAVTATDE....WVPIRPGTDAALCEAIAYVLIEDG.........YANIPF
S_sp.YLB_09_FS418_RS01245                               251  DPRYTDSMLGKESK....WLPIRPGTDAAFAEAIAYQMIS......SGWVDNNSLNF
S_sp.YLB_09_FS418_RS01520                               264  DPRYTDTCLGREDD....WLGIRPGTDGALIEAIFHQVLV........AYGYDSETN
S_sp.YLB_09_FS418_RS02865                               251  DPRYTDSMLGKESK....WLPIRPGTDAAFAEAIAYQMIS......SGWVDNNSLNF
S_sp.YLB_09_FS418_RS03140                               264  DPRYTDTCLGREDD....WLGIRPGTDGALIEAIFHQVLV........AYGYDSETN
S_sp.YLB_09_FS418_RS25020                               266  DPRFTDTAVAAHDE....WVPIRPGTDAALCEGIAHHLISND.........LHEKEF
S_sp.YLB_09_FS418_RS25055                               266  DPRFTDTAVTATDE....WVPIRPGTDAALCEAIAYVLIEDG.........YANIPF
S_sp._WPAGA9_IGB07_RS19235                              250  DPRYTDSMTGKESS....WLPIRPGTDAALAEAIAYLMIS......SGWVDANSIDF
S_sp._ARC9_LZ_GUY17_RS19100                             257  EPRYTDSMRGNEDE....WLAIRPGTDAALIEAVIYQMIS......SGWVDQHAKPF
S_psychromarinicola_strain_M2_EGC80_RS00660             252  DPRYTDSAVGKEDQ....WLAIRPGTDAALFEALAYEWIT......TNTVN...QAF
S_psychromarinicola_strain_M2_EGC80_RS06745             257  EPRYTDSMRGNEDE....WLAIRPGTDAALIEAVIYQMIN......SGWVDQNAKPF
S_psychromarinicola_strain_M2_EGC80_RS11440             246  DPRYTDSMLGREES....HYFVRPGTDAALCEAMAHHLIS.........TDQVD.TSF
S_sp.Actino-trap-3_CXF80_RS10115                        252  DPRYTDSAVGKEDQ....WLAIRPGTDAALFEALAYEWIT......TNTVN...QAF
S_sp.Actino-trap-3_CXF80_RS15635                        257  EPRYTDSMRGNEDE....WLAIRPGTDAALIEAVIYQMIN......SGWVDQNAKPF
S_livingstonensis_strain_LMG_19866_EGC82_RS20515        257  EPRYTDSMRGNEDE....WLAIRPGTDAALIEAVIYQMIS......SGWVDQHAKPF
Ferrimonas_lipolytica_strain_S7_HER31_RS14690           252  DPRCSDSALGDQHS....WMAIRPGTDAALCEALAHEIIS..........RGAADDAF
Ferrimonas_lipolytica_strain_S7_HER31_RS15965           257  DPRYTDSNLGKEDE....WLPIRPGTDGAMAAAIAYEMMKGSDEDHSDSWVELNSRDF
Ferrimonas_lipolytica_strain_S7_HER31_RS04980           248  DPRYTDSCLGREQE....FHFVRPGTDAALIEGMAYHLIE.........NNLIN.ETY
S_sp.ISTPL2_CCLCJOKE_1_HUB64_RS13995                    250  DPRYTDSMAGKENQ....WLPIRPGTDAAFAEAIAYEMIS......SGWVDTHSKAF
S_japonica_strain_KCTC_22435_SJ2017_RS00815             250  DPRYTDSMTGKESS....WLPIRPGTDAALAEAIAYLMIS......EGWVDRNSIDF
S_sp.8A_M2897_RS06500                                   250  DPRYTDSMAGKESQ....WLPIRPGTDAAFAEAIAYEMIS......SGWVDTHSKAF
S_woodyi_ATCC_51908_Swoo_0233                           251  DPRYTDSMLGKESK....WLPIRPGTDAAFAEAIAYQMIS......SGWVDANSLDF
Ferrimonas_balerica_DSM_9799_Fbal_2476                  255  DPRYTESAGGKEQQ....WLPIRPGTDAALVAGIVYALWE.........IGAVDDAH
Ferrimonas_balerica_DSM_9799_Fbal_3621                  253  DPRYTDTTLGKEDQ....WLPIRPGTDGALAEAIAYQMIS......TGWVDQNSKAF
S_frigidimarina_NCIMB_400_Sfri_3128                     252  DPRYTDSAVGKEDQ....WLAIRPGTDAALFEALAYEWIT......TNTVN...QAF
S_frigidimarina_NCIMB_400_Sfri_3684                     251  DPRYTDSMAGKEDN....WFPIRPGTDAALCEGIAYEMIS......SGWVDNHSKNF
S_sediminis_HAW-EB3_Ssed_0229                           251  DPRYTDSMLGKESK....WLPIRPGTDAAFAEAIAYQMIS......SGWVDQNSLSF
S_sediminis_HAW-EB3_Ssed_0350                           252  DPRYTDSAVGKEDQ....WLAIRPGTDAALFEALAYEWIT......TNTVD...QAF
S_sediminis_HAW-EB3_Ssed_0359                           266  DPRFTDTAVTANDE....WIPIRPGTDAALCEAIAYVLIEDG.........YADIPF
S_sediminis_HAW-EB3_Ssed_1307                           245  DCRYSDSAMGRESE....YYPCRPGTDAALCEAMAYVLIT.........EELLD.EQF
S_sediminis_HAW-EB3_Ssed_1404                           250  DPRYSDTAVSSQCH....WLAIRPGTDGALCEALAYEIIT.........QGAADEAF
S_sediminis_HAW-EB3_Ssed_2923                           253  DPRYTDTAVGKEDQ....WIAIRPGTDAALFEALAYEWIT......QGKVD...QAF
S_sp.KX20019_JK628_RS15600                              251  DPRYTDSMLGKEST....WLPIRPGTDAALAEAVAYQMIN......SGWVDANSLDF
S_sp.KX20019_JK628_RS20550                              252  DPRYTDSAVGKEDQ....WLAIRPGTDAALFEALAYEWIT......TNTVD...QAF
Ferrimonas_sp.SCSIO_43195_J8Z22_RS00580                 264  DPRYTDSCLGREDD....WLGIRPGTDGAFIEALFHEILI........AYQYDEQAN
Ferrimonas_sp.SCSIO_43195_J8Z22_RS11605                 259  DPRYTDSALGKEDQ....WIPVRPGTDAALMEALAFELIT......NNWID...QSF
Pseudomonas_sp.SCT_BHD08_RS22425(idrA)                  260  TPHMTMGVAWSVKAGRGLWLPIIPGTDTVLHMALARIIIEN.........DWQDQPF
Denitromonas_sp.IR12_I8J34_RS03780(idrA)                268  TPHRTMGVAWAESTGRGMWLPIIPGTDTVLHLALARIIVEN.........GWQDQAF
```

```
                                                                      .         .         .                        .
S_oneidensis_MR-1_dmsA                                      297  LDKYCVGFDKASLEKLVAEFTASGDPK.............KTQYLPYIDVED....N
S_oneidensis_MR-1_SO_4358                                   329  LQSKVYGYGAEAAFDHYPEL..............................PLA...KS
S_sp._LZH-2_JM642_14190                                     297  LDKYCVGFDKASLEKLVAEFTASGDPK.............KTQYLPYIDVED....N
S_xiamenensis_NUITM-VS1_NUITMVS1_29540                      297  LDKYCVGFDKASLEKLVAEFTASGDPK.............KTQYLPYIDVED....N
S_putrefaciens_strain_FDAARGOS_681_FOB89_20615              297  LDKYCVGFDKASLEKLVEEFTASGDPK.............KTQYLPYIDVED....N
S_sp._MR-4_Shewmr4_3676                                     300  LDKYCVGYDKASLEKTKAEFEASGDAS.............KVEYAKFIDVEE....N
S_fidelis_ATCC-BAA-318__L884__L884_RS0114160                299  VLEHSVGFNKQSIEDQKAIFAASDDAT.............KQEYAKVMNSDE....N
S_piezotolerans_WP3_SWP_RS15450                             298  INKYAVGFDAASIQAQKDIFAASDDAT.............KQEYAAVMNPEE....N
S_schlegeliana_strain_JCM_JMA39_11561_RS09490               299  VLENSVGFTKQSIEDQIAIFAASGDAT.............KQEYAKVMNSDD....N
S_marisflavi_strain_EP1_CFF01_RS01175                       298  LDKYCVGYDKASLEQAKADLEISNDSD.............KRSYAQFIDIEE....N
S_sp.SUN_WT4_FJQ87_RS02760                                  298  INTHVVGYDAASIAVQQAKFEASNDAT.............KQEYAKIMDSQD....N
S_sp.MBTL60-112-B2_K5Q73_RS09520                            299  VLEHSVGFNKKSIEDQKAIFAASSDAT.............DQEYAKVMNSDE....N
S_sp.MBTL60_112_B1_K5Q83_RS07010                            299  VLEHSVGFNKKSIEDQKAIFAASSDAT.............DQEYAKVMNSDE....N
S_enrypsychrophilus_strain_YLB-08_FM038_RS01260             298  INKYAVGFDAASIAQQKEIFAASGDAT.............KQEYAKVMKPEE....N
S_enrypsychrophilus_strain_YLB-08_FM038_RS01535             309  TFADKRGNWANTAILGLTNKSLEEYKTKLELELESGEITDGNAQYAEHIEPED....N
S_enrypsychrophilus_strain_YLB-08_FM038_RS23490             310  LEKFCMGFTADTLPEGA.................................PAN...GD
S_enrypsychrophilus_strain_YLB-08_FM038_RS23525             310  LEQYTYGY..............................................DK
S_sp.YLB_09_FS418_RS01245                                   298  INKYAVGFDAASIAQQKEIFAASGDAT.............KQEYAKVMKPEE....N
S_sp.YLB_09_FS418_RS01520                                   309  TFADKRGNWANTAILGLTNKSLEEYKTKLELELESGEITDGNAQYAEHIEPED....N
S_sp.YLB_09_FS418_RS02865                                   298  INKYAVGFDAASIAQQKEIFAASGDAT.............KQEYAKVMKPEE....N
S_sp.YLB_09_FS418_RS03140                                   309  TFADKRGNWANTAILGLTNKSLEEYKTKLELELESGEITDGNAQYAEHIEPED....N
S_sp.YLB_09_FS418_RS25020                                   310  LEKFCMGFTADTLPEGA.................................PAN...GD
S_sp.YLB_09_FS418_RS25055                                   310  LEQYTYGY..............................................DK
S_sp._WPAGA9_IGB07_RS19235                                  297  INKNSVGYDAASIEAQKQIFLNSGDAK.............KIEYAAAMDSEE....N
S_sp._ARC9_LZ_GUY17_RS19100                                 304  LDKYAVGYDEASLVNTKASVMLNADPF.............IASHAEFIDPAQ....N
S_psychromarinicola_strain_M2_EGC80_RS00660                 296  LDKYCVGYDEKTMPAG..............................IGYEE....S
S_psychromarinicola_strain_M2_EGC80_RS06745                 304  LDKYAVGYDKASLVNTKASVTLNADPF.............IASHAEFIDPDQ....N
S_psychromarinicola_strain_M2_EGC80_RS11440                 290  LKEKCYGFWAEPEMPDPHNP..................SLTLPAVPYEE....C
S_sp.Actino-trap-3_CXF80_RS10115                            296  LDKYCVGYDEKTMPAG..............................IGYEE....S
S_sp.Actino-trap-3_CXF80_RS15635                            304  LDKYAVGYDKASLVNTKASVTLNADPF.............IASHAEFIDPDQ....N
S_livingstonensis_strain_LMG_19866_EGC82_RS20515            304  LDKYAVGYDEASLVNTKASVTLNADPF.............IASHAEFIDPAQ....N
Ferrimonas_lipolytica_strain_S7_HER31_RS14690               296  LAKYCVGYDQSTLPDSAAEFS.....................................D
Ferrimonas_lipolytica_strain_S7_HER31_RS15965               311  LNSYTVGYDAASIRAAIAADPTL.QPK............HDDGTIAITVDEMAANN
Ferrimonas_lipolytica_strain_S7_HER31_RS04980               292  LREKCYGFFAEDEMVSPHDS..................DRVLPAVSEED....S
S_sp.ISTPL2_CCLCJOKE_1_HUB64_RS13995                        297  LDKYCVGFDKASLEKLVAEFTASGDPK.............KTQYLPYIDVED....N
S_japonica_strain_KCTC_22435_SJ2017_RS00815                 297  INKNSVGYDAASIEAQKQIFLNSGDAK.............KIEYAAAMDSEE....N
S_sp.8A_M2897_RS06500                                       297  LDKYCVGFDKASLEKLVAEFTASGDPK.............KTQYLPYIDVED....N
S_woodyi_ATCC_51908_Swoo_0233                               298  INKYAVGFDADSIAAQQALFAASGDAT.............KQEYAKVMKPEE....N
Ferrimonas_balerica_DSM_9799_Fbal_2476                      299  VDRYAVGWTEASLPESAK............................GKNA....S
Ferrimonas_balerica_DSM_9799_Fbal_3621                      300  LDKHAIGYDKASLEQAKVDNP.................DYADLINSDE....N
S_frigidimarina_NCIMB_400_Sfri_3128                         296  LDKYCVGYDEKTMPEG..............................VGYEE....S
S_frigidimarina_NCIMB_400_Sfri_3684                         298  LDKFCVGYDKESLINLKAEFEISEDPE.............KKAYIPYINPND....N
S_sediminis_HAW-EB3_Ssed_0229                               298  INKYAVGFDAASITAQKELFANSGDAT.............KQEYAKVMKPEE....N
S_sediminis_HAW-EB3_Ssed_0350                               296  LDKYCVGYDEKTMPAG..............................VGYEE....S
S_sediminis_HAW-EB3_Ssed_0359                               310  LEQYTHGY..............................................DK
S_sediminis_HAW-EB3_Ssed_1307                               289  LSDKCYGFRAEPSIQDARDS..................SKMLPEVVYED....S
S_sediminis_HAW-EB3_Ssed_1404                               294  LAKYCVGYDQSTLPASAAANS.....................................D
S_sediminis_HAW-EB3_Ssed_2923                               297  LNKYCVGYDEKTLPEG..............................VDYGE....S
S_sp.KX20019_JK628_RS15600                                  298  INKYAVGFDAASIQAQKEIFAASGDET.............KQEYAAVMNPEE....N
S_sp.KX20019_JK628_RS20550                                  296  LDKYCVGYDEKTMPAG..............................IGYEE....S
Ferrimonas_sp.SCSIO_43195_J8Z22_RS00580                     309  HFADPRGNWANAVILGLTKASLEAYAD....QLKDG...DGNYEFKDYIQPED....N
Ferrimonas_sp.SCSIO_43195_J8Z22_RS11605                     303  LNSHCLGFDEHTLPAG..............................QDYDA....T
Pseudomonas_sp.SCT_BHD08_RS22425(idrA)                      308  IDKWIANSWEVDSGYGRGTRNTGWQWR..................TTWGTWQSDWQD
Denitromonas_sp.IR12_I8J34_RS03780(idrA)                    316  IDKWVANKWEVDSGYGRGTRNTGWQWR..................TTWGKWQSDWKD
```

```
S_oneidensis_MR-1_dmsA                              337  YRNYILGEGKFADNG.........PRTPAWAAKICGISEANIKSVASKIMNAKAPFIV
S_oneidensis_MR-1_SO_4358                           354  YKGHILGLD.DGQAK...........TPEWAAAITGIPVDTIRALASEIAAAKTPFFI
S_sp._LZH-2_JM642_14190                             337  YRNYILGEGKFVDNG.........PRTPAWAAKICGISEANIKSVASKIMNAKAPFIV
S_xiamenensis_NUITM-VS1_NUITMVS1_29540              337  YRNYILGEGKFVDNG.........PRTPAWAAKICGISEANIKSVASKIMNAKAPFIV
S_putrefaciens_strain_FDAARGOS_681_FOB89_20615      337  YRDYILGEGKFVDKG.........PRTPAWAAKICGISEANIKSVASKIMNAKAPFIV
S_sp._MR-4_Shewmr4_3676                             340  YRDYILGVGKFTEA...........KTPAWAATVCGISEANIKAIAQKIMNAKAPYII
S_fidelis_ATCC-BAA-318__L884__L884_RS0114160        339  YQDYILGKGIYTDA...........KTPEWAEKICGISAEKIRYIAGKLMTAKAPYIV
S_piezotolerans_WP3_SWP_RS15450                     338  YRDYILGLGIYADAP.........KKTAEWAEKITGIPAAQIKQIATDLQNAKAPYIV
S_schlegeliana_strain_JCM_JMA39_11561_RS09490       339  YQDYILGQGIYTDA...........KTPEWAESICGISADKIRYIADKLMTANAPYIV
S_marisflavi_strain_EP1_CFF01_RS01175               338  YHDYILGVGKFTEA...........KTPEWAAKICGISASNIKQIADKIMNASAPFIT
S_sp.SUN_WT4_FJQ87_RS02760                          338  YHDYILGLNKFAGP..........ARTPEWAESITGISADKIREVADDLMAAKAPYIV
S_sp.MBTL60-112-B2_K5Q73_RS09520                    339  YQDYILGQGIYTDA...........KTPEWAEKICGISAEKIRYIAGKLMTAKAPYIV
S_sp.MBTL60_112_B1_K5Q83_RS07010                    339  YQDYILGQGIYTDA...........KTPEWAEKICGISAEKIRYIAGKLMTAKAPYIV
S_enrypsychrophilus_strain_YLB-08_FM038_RS01260     338  YRDYILSQGIYADQP.........LKTPEWAEKITGIPAAQLEQIATDLQNAKAPYIV
S_enrypsychrophilus_strain_YLB-08_FM038_RS01535     363  LYDYIMGTFTGKA............KTPEWAAPICGVPAGKIREVAQDLLSADAPFIC
S_enrypsychrophilus_strain_YLB-08_FM038_RS23490     332  YYSHIMGLSSDGIEK...........TPEWAESITGVSAEKIREIADALANANAPFVT
S_enrypsychrophilus_strain_YLB-08_FM038_RS23525     320  YEEYITGVT.DGIAR...........TPERAESICGIPAEKIREIAQAIGTASAPFVT
S_sp.YLB_09_FS418_RS01245                           338  YRDYILSQGIYADQP.........LKTPEWAEKITGIPAAQLEQIATDLQNAKAPYIV
S_sp.YLB_09_FS418_RS01520                           363  LYDYIMGTFTGKA............KTPEWAAPICGVPAGKIREVAQDLLSADAPFIC
S_sp.YLB_09_FS418_RS02865                           338  YRDYILSQGIYADQP.........LKTPEWAEKITGIPAAQLEQIATDLQNAKAPYIV
S_sp.YLB_09_FS418_RS03140                           363  LYDYIMGTFTGKA............KTPEWAAPICGVPAGKIREVAQDLLSADAPFIC
S_sp.YLB_09_FS418_RS25020                           332  YYSHIMGLSSDGIEK...........TPEWAESITGVSAEKIREIADALANANAPFVT
S_sp.YLB_09_FS418_RS25055                           320  YEEYITGVT.DGIAR...........TPERAESICGIPAEKIREIAQAIGTASAPFVT
S_sp._WPAGA9_IGB07_RS19235                          337  YHDYIMGQGKFADRE.........ARTPEWAAKITGISEANIKKVADDLMASEKPYII
S_sp._ARC9_LZ_GUY17_RS19100                         344  YHDYIMGQGIFELEG.........AKTPAWAAVVCGVPANKIEQLATAIMAAEAPYIS
S_psychromarinicola_strain_M2_EGC80_RS00660         318  YKAYILDNATVGDSIPGV.....NAKTPEWAAAITGIEAHLIVELARELAAARAPFIQ
S_psychromarinicola_strain_M2_EGC80_RS06745         344  YHDYIMGQGIFEQDG.........AKTPDWAAVVCGVPAYKIEQLATAMMAAEAPYIS
S_psychromarinicola_strain_M2_EGC80_RS11440         322  YEAHILGIKDGIA............KTPEWGSKITGVPAETIREVANRLAQAKAPFVI
S_sp.Actino-trap-3_CXF80_RS10115                    318  YKAYILDNTTVGDSIPGV.....NAKTPEWAAAITGIEAHLIVELARELAAARAPFIQ
S_sp.Actino-trap-3_CXF80_RS15635                    344  YHDYIMGQGIFEQDG.........AKTPDWAAVVCGVPAYKIEQLATAMMAAEAPYIS
S_livingstonensis_strain_LMG_19866_EGC82_RS20515    344  YHDYIMGQGIFELEG.........AKTPAWAAVVCGVPANKIEQLATAIMAAEAPYIS
Ferrimonas_lipolytica_strain_S7_HER31_RS14690       318  FKSHILGLAPDDVP...........KDVAYASAITGISEAKITELADKLIAADKPFVT
Ferrimonas_lipolytica_strain_S7_HER31_RS15965       354  YKDYILGSGKFDAITGTAPDYEKGAKDAEWAEAICGIPADKLREIASELMASSNPYIQ
Ferrimonas_lipolytica_strain_S7_HER31_RS04980       324  YRGHILGLKDGIA............KTPAWAAKISGVPESTIKSTAEKLAAAKSPFVV
S_sp.ISTPL2_CCLCJOKE_1_HUB64_RS13995                337  YRNYILGEGKFVDNG.........PRTPAWAAKICGISEANIKSIASKIMNARAPFIV
S_japonica_strain_KCTC_22435_SJ2017_RS00815         337  YHDYIMGQGKFADRE.........ARTPEWAAKITGISEANIKKVADDLMASEKPYII
S_sp.8A_M2897_RS06500                               337  YRNYILGEGKFVDNG.........PRTPAWAAKICGISEANIKSVASKIMNAKAPFIV
S_woodyi_ATCC_51908_Swoo_0233                       338  YRDYILSQGIYSDQP.........LKTPEWAEKITGIPAAQLEQIATDLQNAKAPYIV
Ferrimonas_balerica_DSM_9799_Fbal_2476              322  YKAYILGDRD............GVAKTPEWAASITGVPAATIRELATKLHRSKAPFIQ
Ferrimonas_balerica_DSM_9799_Fbal_3621              332  YHDYIMGVGIYSMP...........HTPEWAAAKCGVPVRQIEALADKIMNAKTPYIT
S_frigidimarina_NCIMB_400_Sfri_3128                 318  YKAYILDNAKVGDLTPGV.....NAKTPEWAAAITGIEAHLIVELARELAAARAPFIQ
S_frigidimarina_NCIMB_400_Sfri_3684                 338  YKDYILGEGTFISTG.........PKTPSWAAKICGISETHIKSIATKLMNARAPFIV
S_sediminis_HAW-EB3_Ssed_0229                       338  YRDYILSQGVYADQP.........LKTSEWAEKITGIPAAQLEQIATDLQNAKAPYIV
S_sediminis_HAW-EB3_Ssed_0350                       318  YKAYILDNTTVGSSIPGV.....NAKTPEWAAAITGIEAHIIVELARELAAARAPFIQ
S_sediminis_HAW-EB3_Ssed_0359                       320  YQDYITGVT.DGTTK...........TPERAEGICGIPAEKIREIAQAIGAASAPFVT
S_sediminis_HAW-EB3_Ssed_1307                       321  YEAHILGVKDGQP............KTPQWAAEICGIPADSIRNIARQIGNAKAPYLA
S_sediminis_HAW-EB3_Ssed_1404                       316  YKSHILGQGPDGVV...........KTPEYAAAITGICAEEIRLLATKLIAAKAPFIS
S_sediminis_HAW-EB3_Ssed_2923                       319  YKSYILDNATIGELTPGD.....NAKTPAWAAGITGIPEHTIIELARELAAAKAPFIK
S_sp.KX20019_JK628_RS15600                          338  YRDYILGLGIYADAP.........KKTAQWAEEITGIPAGKIQQMATDLQEAQAPYIV
S_sp.KX20019_JK628_RS20550                          318  YKAYILDNATVGDSTPGV.....NAKTPEWAAAITGIEAHLIVELARELAAARAPFIQ
Ferrimonas_sp.SCSIO_43195_J8Z22_RS00580             356  LFDHIMGTHDGQA............KTPEWAASICGIPADRIRAVAEDLLAADAPFIC
Ferrimonas_sp.SCSIO_43195_J8Z22_RS11605             325  YKSYILDNTQPGSLTAGS.....NAKTPEWASAICGVPADDIRQLAKDLADATTPFVH
Pseudomonas_sp.SCT_BHD08_RS22425(idrA)              347  YRKFILAQEES...............KLDVAAQITGLSADDIRTAAEWIAKPKADGSH
Denitromonas_sp.IR12_I8J34_RS03780(idrA)            355  YSAWILKQKEG...............ELETAAKITGLRAEDIRKAAEWIAKPKADGTR
```

S_oneidensis_MR-1_dmsA	386	TGAGVNRH.....ANGEQAMRACYMLSFLTGKVGQPGVSN..GALPYQGSLNR.....
S_oneidensis_MR-1_SO_4358	400	QGWGIQRA.....QNGENAARSLFMLPILLGKVGDKGTNF..CSVPGAKTYAKPI...
S_sp._LZH-2_JM642_14190	386	TGAGVNRH.....ANGEQAMRACYMLSFLTGKVGQPGVSN..GALPYQGSLNR.....
S_xiamenensis_NUITM-VS1_NUITMVS1_29540	386	TGAGVNRH.....ANGEQAMRACYMLSFLTGKVGQPGVSN..GALPYQGSLNR.....
S_putrefaciens_strain_FDAARGOS_681_FOB89_20615	386	TGAGVNRH.....ANGEQAMRACYMLSFLTGKVGQPGVSN..GALPYQGSLNR.....
S_sp._MR-4_Shewmr4_3676	387	IGAGVNRH.....ANGEQAMRACYMLPFLTGKVGQPGVNN..GALPVQGSMSK.....
S_fidelis_ATCC-BAA-318__L884__L884_RS0114160	386	VGAGVNRQ.....ANGEQSMRALYMLSVLTGKLGTRGASN..GELPSMSSMSR.....
S_piezotolerans_WP3_SWP_RS15450	387	VGAGVNRQ.....ANGEQSMRALYMLSVLTGKLGTLGASN..GELPSMSGMYR.....
S_schlegeliana_strain_JCM_JMA39_11561_RS09490	386	IGAGVNRQ.....ANGEQSMRALYMLSVLTGKLGTRGASN..GELPYMSSMSR.....
S_marisflavi_strain_EP1_CFF01_RS01175	385	IGAGVNRH.....ANGEQAMRSCYMLPLLTGKLGKAGVSN..GALPVQGGMSK.....
S_sp.SUN_WT4_FJQ87_RS02760	386	IGAGVNRQ.....ANGEQSMRALYMLSVLTGKLGTRGASN..GELPYMSGMGR.....
S_sp.MBTL60-112-B2_K5Q73_RS09520	386	VGAGVNRQ.....ANGEQSMRSLYMLSVLTGKLGTRGASN..GELPYMSSMSR.....
S_sp.MBTL60_112_B1_K5Q83_RS07010	386	VGAGVNRQ.....ANGEQSMRSLYMLSVLTGKLGTRGASN..GELPYMSSMSR.....
S_enrypsychrophilus_strain_YLB-08_FM038_RS01260	387	VGAGVNRQ.....ANGEQSMRALYMLSVLTGKLGTLGASN..GELPFMSSMSR.....
S_enrypsychrophilus_strain_YLB-08_FM038_RS01535	409	SGQGIQRN.....ACGEQVMRAIYTLPVLLNAIGRRGTNN..GDLPMVAAAGP.....
S_enrypsychrophilus_strain_YLB-08_FM038_RS23490	379	QGFGMQRQ.....GNGENNAKAVLMLPILIGKIVGKGINH..GGTPGAKDLAH.....
S_enrypsychrophilus_strain_YLB-08_FM038_RS23525	366	QGFGPQRQ.....GNGENNARAVMMLPILVGKISGAGTNH..GGTPGNDGLSH.....
S_sp.YLB_09_FS418_RS01245	387	VGAGVNRQ.....ANGEQSMRALYMLSVLTGKLGTLGASN..GELPFMSSMSR.....
S_sp.YLB_09_FS418_RS01520	409	SGQGIQRN.....ACGEQVMRAIYTLPVLLNAIGRRGTNN..GDLPMVAAAGP.....
S_sp.YLB_09_FS418_RS02865	387	VGAGVNRQ.....ANGEQSMRALYMLSVLTGKLGTLGASN..GELPFMSSMSR.....
S_sp.YLB_09_FS418_RS03140	409	SGQGIQRN.....ACGEQVMRAIYTLPVLLNAIGRRGTNN..GDLPMVAAAGP.....
S_sp.YLB_09_FS418_RS25020	379	QGFGMQRQ.....GNGENNAKAVLMLPILIGKIVGKGINH..GGTPGAKDLAH.....
S_sp.YLB_09_FS418_RS25055	366	QGFGPQRQ.....GNGENNARAVMMLPILVGKISGAGTNH..GGTPGNDGLSH.....
S_sp._WPAGA9_IGB07_RS19235	386	AGAGVNRQ.....ANGEQTVRAIYMLGVLAGKLGREGSSN..GELPNMTNMFR.....
S_sp._ARC9_LZ_GUY17_RS19100	393	AGAGISRH.....ANGDQATRAVYTLAIMTGKIGRAGVNS..GAMPTSYSFGV.....
S_psychromarinicola_strain_M2_EGC80_RS00660	371	IAASLNRQ.....AAGENNTRAGYMLPILLGQLGLPGTNC..GGLCKGSSLHA.....
S_psychromarinicola_strain_M2_EGC80_RS06745	393	AGAGISRH.....ANGDQATRAVYTLAIMTGKIGRAGVNS..GAMPSSYSFGV.....
S_psychromarinicola_strain_M2_EGC80_RS11440	368	AGWSIQRQ.....LNGEDNIRAISTLSLMVGAVGKRGTTN..GDMAFS..EIA.....
S_sp.Actino-trap-3_CXF80_RS10115	371	IAASLNRQ.....AAGENNTRAGYMLPILLGQLGLPGTNC..GGLCKGSSLHA.....
S_sp.Actino-trap-3_CXF80_RS15635	393	AGAGISRH.....ANGDQATRAVYTLAIMTGKIGRAGVNS..GAMPSSYSFGV.....
S_livingstonensis_strain_LMG_19866_EGC82_RS20515	393	AGAGISRH.....ANGDQATRAVYTLAIMTGKIGRAGVNS..GAMPTSYSFGV.....
Ferrimonas_lipolytica_strain_S7_HER31_RS14690	365	QGLGPQRH.....ACGEQTVRAITMLPLLLGKVGKEGTNT..GFMPSTNTGAF.....
Ferrimonas_lipolytica_strain_S7_HER31_RS15965	412	IGAGPNRQ.....AAGEQTMRSLYMLSILAGKLGQAGTNT..GELPSNFRHNT.....
Ferrimonas_lipolytica_strain_S7_HER31_RS04980	370	AGWGMQRQ.....FTGEDNVRSVTTLALMVGAVGNRGNST..GELPD..........
S_sp.ISTPL2_CCLCJOKE_1_HUB64_RS13995	386	TGAGVNRH.....ANGEQAMRACYMLSFLTGKVGQPGVSN..GALPYQGSLNR.....
S_japonica_strain_KCTC_22435_SJ2017_RS00815	386	AGAGVNRQ.....ANGEQTVRAIYMLGVLAGKLGREGSSN..GELPNMTNMFR.....
S_sp.8A_M2897_RS06500	386	TGAGVNRH.....ANGEQAMRACYMLSFLTGKVGQPGVSN..GALPYQGSLNR.....
S_woodyi_ATCC_51908_Swoo_0233	387	VGAGVNRQ.....ANGEQSMRALYMLSVLTGKLGTLGSSN..GELPYMSSMSR.....
Ferrimonas_balerica_DSM_9799_Fbal_2476	368	MGWGLQRQ.....ANGENSARAIYTLVAIVGKFGLPGTSNAVGAHNGTHAYIT.....
Ferrimonas_balerica_DSM_9799_Fbal_3621	379	IGAGCNRH.....ACGEQTMRALYMLPILTGKLGESGVNN..GELPRNFGLGR.....
S_frigidimarina_NCIMB_400_Sfri_3128	371	IAASLNRQ.....AAGENNTRAGYMLPILLGQLGLPGTNC..GGLCKGSSLHA.....
S_frigidimarina_NCIMB_400_Sfri_3684	387	AGAGINRH.....ANGEQAMRSCYMLSFLTGKVGQPGVSN..GALPSQGSLSR.....
S_sediminis_HAW-EB3_Ssed_0229	387	VGAGVNRQ.....ANGEQSMRALYMLSVLTGKLGTLGASN..GELPSMSGMYR.....
S_sediminis_HAW-EB3_Ssed_0350	371	IAASLNRQ.....AAGENNTRAGYMLPILLGQLGLPGTNC..GGLCKGSFLHA.....
S_sediminis_HAW-EB3_Ssed_0359	366	QGFGPQRQ.....GNGENNARAVMMLPLLVGKISGDGTNH..GGTPGNDGLSH.....
S_sediminis_HAW-EB3_Ssed_1307	367	PGLGMQRH.....INGEDNIRSVMTLGMLVGAIGKRGTNT..GDYPKSPRKIY.....
S_sediminis_HAW-EB3_Ssed_1404	363	QGLGVQRH.....ACGEQAVRSILMLPLLLGKVGQSGTNT..GFQPARNVYPL.....
S_sediminis_HAW-EB3_Ssed_2923	372	LGLTLNRH.....AAGENNMRAGYMLPILLGQLGLPGTNN..GGDSSGGHCVV.....
S_sp.KX20019_JK628_RS15600	387	AGAGVNRQ.....ANGEQTVRALYMLSVLTGKLGTLGSSN..GELPYMSGMYR.....
S_sp.KX20019_JK628_RS20550	371	IAASLNRQ.....AAGENNTRAGYMLPILLGQLGLPGTNC..GGLCKGSSLHA.....
Ferrimonas_sp.SCSIO_43195_J8Z22_RS00580	402	SGQGVQRS.....ACGEQVMRQIYTLPVLLNAIGRRGTNN..GDLPMVAAAGP.....
Ferrimonas_sp.SCSIO_43195_J8Z22_RS11605	378	IAASLNRQ.....AAGENNIRACYMLPILLGQVGLPGTNN..GAMCSGASLPI.....
Pseudomonas_sp.SCT_BHD08_RS22425(idrA)	390	PKTSFMCEKGNYWSNNYMNSASFASLGLICGSGNRKGRMISRGGGHQRGGLSAGGNSE
Denitromonas_sp.IR12_I8J34_RS03780(idrA)	398	VKASFMLEKGNYWTNNYMNSASLASLGLICGSGNRPGQMISRGGGHQRGGMSAGGGSG

**S_oneidensis_MR-1_dmsA**	**432**	.AGMS..................GGSNPVK...ESISFFTWAEAIE...RGHEMTARK
**S_oneidensis_MR-1_SO_4358**	**448**	VAGMEADS...........VGNSVGSAVINGTEFTIPGYKWLEGVQ...RPLNSRDDG
**S_sp._LZH-2_JM642_14190**	**432**	.AGMS..................GGNNPVK...ESISFFTWAEAIE...RGHEMTARK
**S_xiamenensis_NUITM-VS1_NUITMVS1_29540**	**432**	.AGMS..................GGNNPVK...ESISFFTWAEAIE...RGHEMTARK
**S_putrefaciens_strain_FDAARGOS_681_FOB89_20615**	**432**	.AGMS..................GGSNPVK...ESISFFTWAEAIE...RGHEMTARK
**S_sp._MR-4_Shewmr4_3676**	**433**	.SSLS..................GLSNPIK...EAISFFTFSEAIE...RGAEMTARK
**S_fidelis_ATCC-BAA-318__L884__L884_RS0114160**	**432**	.AGIP..................TGKNEVK...ESISFFTWSEAIN...HGETMTARS
**S_piezotolerans_WP3_SWP_RS15450**	**433**	.AGIP..................AGSNPVK...EKISFFTWSEAIH...HGETMTARS
**S_schlegeliana_strain_JCM_JMA39_11561_RS09490**	**432**	.AGIP..................TGSNPVK...ESISFFTWSEAIL...NGETMTARS
**S_marisflavi_strain_EP1_CFF01_RS01175**	**431**	.AGLG..................GLSNPIK...ESISFYTFSDAII...RGHEMTGRK
**S_sp.SUN_WT4_FJQ87_RS02760**	**432**	.AGIP..................TGSNPVE...ESISFFTWSEAIV...NGKDFTART
**S_sp.MBTL60-112-B2_K5Q73_RS09520**	**432**	.AGIP..................TGTNKVT...ESISFFTWSEAIN...HGETMTARS
**S_sp.MBTL60_112_B1_K5Q83_RS07010**	**432**	.AGIP..................TGTNKVT...ESISFFTWSEAIN...HGETMTARS
**S_enrypsychrophilus_strain_YLB-08_FM038_RS01260**	**433**	.SGIP..................TGSNPVK...ESISFFTWSEAIH...NGETMTARS
**S_enrypsychrophilus_strain_YLB-08_FM038_RS01535**	**455**	.GAAMIPMAFWSDPTVPGFIGGTAPASVVK...AKIPTYKTFEAMV...RGHEMTERS
**S_enrypsychrophilus_strain_YLB-08_FM038_RS23490**	**425**	YKMMPADS...........LKDLMNLGFGD...VSIPAASHLDAIE...FGTEMKPSS
**S_enrypsychrophilus_strain_YLB-08_FM038_RS23525**	**412**	YAMMPADS...........LKDLMNMKIMD...VTIPAASHLDAIE...FGTQMKPST
**S_sp.YLB_09_FS418_RS01245**	**433**	.SGIP..................TGSNPVK...ESISFFTWSEAIH...NGETMTARS
**S_sp.YLB_09_FS418_RS01520**	**455**	.GAAMIPMAFWSDPTVPGFIGGTAPASVVK...AKIPTYKTFEAMV...RGHEMTERS
**S_sp.YLB_09_FS418_RS02865**	**433**	.SGIP..................TGSNPVK...ESISFFTWSEAIH...NGETMTARS
**S_sp.YLB_09_FS418_RS03140**	**455**	.GAAMIPMAFWSDPTVPGFIGGTAPASVVK...AKIPTYKTFEAMV...RGHEMTERS
**S_sp.YLB_09_FS418_RS25020**	**425**	YKMMPADS...........LKDLMNLGFGD...VSIPAASHLDAIE...FGTEMKPSS
**S_sp.YLB_09_FS418_RS25055**	**412**	YAMMPADS...........LKDLMNMKIMD...VTIPAASHLDAIE...FGTQMKPST
**S_sp._WPAGA9_IGB07_RS19235**	**432**	.GSIP..................TGTNPVK...EKISFFTWSEAIH...NGETMTART
**S_sp._ARC9_LZ_GUY17_RS19100**	**439**	.AGMP..................TGSNPVD...VTIPVYTWSDAVV...RGEDFSGTS
**S_psychromarinicola_strain_M2_EGC80_RS00660**	**417**	.PSMPTG..................SNPVK...KAISFFTFTQAIE...DGKNMTVLS
**S_psychromarinicola_strain_M2_EGC80_RS06745**	**439**	.AGMQ..................AGSNPVD...VTIPVFTWSDAVV...RGEDFSGTS
**S_psychromarinicola_strain_M2_EGC80_RS11440**	**412**	.TTNAF............FVLPSVPANEKN...AKVRMAAWPRAIL...DGENMTVIK
**S_sp.Actino-trap-3_CXF80_RS10115**	**417**	.PSMPTG..................SNPVK...KAISFFTFTQAIE...DGKNMTVLS
**S_sp.Actino-trap-3_CXF80_RS15635**	**439**	.AGMQ..................AGSNPVD...VTIPVFTWSDAVV...RGEDFSGTS
**S_livingstonensis_strain_LMG_19866_EGC82_RS20515**	**439**	.AGMP..................TGSNPVD...VTIPVYTWSDAVV...RGEDFSGTS
**Ferrimonas_lipolytica_strain_S7_HER31_RS14690**	**411**	.WLSP..................TGTNPVE...TSIPCFLWTEAII...NGENMTAEE
**Ferrimonas_lipolytica_strain_S7_HER31_RS15965**	**458**	.AGMGYS.Y..............ADKDGSK...ASLCFFDWVEAVK...DGKDMDYRS
**Ferrimonas_lipolytica_strain_S7_HER31_RS04980**	**410**	.SKGYL............YPIPPLVANPIA...CAVPVHGWPRLIR...EGETMTVLK
**S_sp.ISTPL2_CCLCJOKE_1_HUB64_RS13995**	**432**	.AGMS..................GGSNPVK...ESISFFTWAEAIE...RGHEMTARK
**S_japonica_strain_KCTC_22435_SJ2017_RS00815**	**432**	.GSIP..................TGTNPVK...EKISFFTWSEAIH...NGETMTART
**S_sp.8A_M2897_RS06500**	**432**	.AGMS..................GGNNPVK...ESISFFTWAEAIE...RGHEMTARK
**S_woodyi_ATCC_51908_Swoo_0233**	**433**	.AGIP..................TGSNPVK...ESISFFTWSEAIH...HGETMTARS
**Ferrimonas_balerica_DSM_9799_Fbal_2476**	**416**	.PGIP..................AGANPVT...KSIPCFLWTDAIERDLQQEPMTALT
**Ferrimonas_balerica_DSM_9799_Fbal_3621**	**425**	.SGMS..................AGSNPES...ASICFHTWAEAIE...RGEEMDALS
**S_frigidimarina_NCIMB_400_Sfri_3128**	**417**	.PSMPTG..................SNPVK...KAISFFTFTQAIE...DGKNMTVLS
**S_frigidimarina_NCIMB_400_Sfri_3684**	**433**	.SGMS..................GLSNPIK...ESISCFTFPDAIE...RGHEMTTRK
**S_sediminis_HAW-EB3_Ssed_0229**	**433**	.AGIP..................TGSNPVK...ESISFFTWSEAIH...NGETMTARS
**S_sediminis_HAW-EB3_Ssed_0350**	**417**	.PFMPTG..................SNPVK...KAISFFTFTQAIE...DGKNMTVLS
**S_sediminis_HAW-EB3_Ssed_0359**	**412**	YAMMPADT...........LQDLMNMKIMD...VTIPAASHLDAIE...FGMQMKPST
**S_sediminis_HAW-EB3_Ssed_1307**	**413**	.SKGLP............TVSEQQEVSIDD...AKISMFSWTQAVE...NGENLTVLR
**S_sediminis_HAW-EB3_Ssed_1404**	**409**	.AFSP..................TGANPVT...AKIPCFLWTDAVV...RGEEMTATT
**S_sediminis_HAW-EB3_Ssed_2923**	**418**	.PRIAEL..................ENPVK...KEISFFTYLQAVE...DGKNMTVLA
**S_sp.KX20019_JK628_RS15600**	**433**	.AGIP..................AGANPVK...EKISFFTWSEAIH...HGETMTARS
**S_sp.KX20019_JK628_RS20550**	**417**	.PSMPTG..................SNPVK...KAISFFTFTQAIE...DGKNMTVLS
**Ferrimonas_sp.SCSIO_43195_J8Z22_RS00580**	**448**	.GAAMIPMAFWSDPTIPGFIGGTAPPAGVK...ASIPTYKTYEAME...RGHEMTYRS
**Ferrimonas_sp.SCSIO_43195_J8Z22_RS11605**	**424**	.ATMPTRG................DLNTVD...KSISFFTFTQAIE...DGENMTVLR
**Pseudomonas_sp.SCT_BHD08_RS22425(idrA)**	**448**	........................WLSPEKYPGRRKKSFNLDLWLM...EGNIRFAWV
**Denitromonas_sp.IR12_I8J34_RS03780(idrA)**	**456**	........................WLSPEKYPGRRKKSFNLDRWMM...NGNVRFAWV

```
                                                                  .         .         .         .         .
S_oneidensis_MR-1_dmsA                                      465  DGIRG........TADLDTPLGAD..IKAVFAINSNALINQHSDCNGTAKILEDDTK.
S_oneidensis_MR-1_SO_4358                                   492  LRHMLGE....EQKKDHDNILELKNPIKFIFGNACGTTLNQTADINHTLDILNNAPE.
S_sp._LZH-2_JM642_14190                                     465  DGIRG........TADLDTPLGAD..IKAVFAINSNALINQHSDCNGTAKILEDDSK.
S_xiamenensis_NUITM-VS1_NUITMVS1_29540                      465  DGIRG........TADLDTPLGAD..IKAVFAINSNALINQHSDCNGTAKILEDDTK.
S_putrefaciens_strain_FDAARGOS_681_FOB89_20615              465  DGIRG........TADLDTPLGAD..IKAVFAINSNALINQHSDCNGTAKILEDDTK.
S_sp._MR-4_Shewmr4_3676                                     466  DGVRG........MPDLDTPLGQN..IKAIFAINSNSLVNQHADSNGTSKILEDDTK.
S_fidelis_ATCC-BAA-318__L884__L884_RS0114160                465  HGVRG........TDGLDTPLGAN..IKFILSASDSSLLNQHAEINKTADIFRN.AE.
S_piezotolerans_WP3_SWP_RS15450                             466  HGVKG........TDDLDTPLGTN..IKVIISASDSTLLNQHAQINNTAEIYQDESG.
S_schlegeliana_strain_JCM_JMA39_11561_RS09490               465  HGVRG........TDGLDTPLGAN..IKFILSASDSSLLNQHAEINNTAEIFRT.AE.
S_marisflavi_strain_EP1_CFF01_RS01175                       464  DGVRG........TEDLDTPLGHD..VKAIFAANSNALINQHSDSNGTHEILQLEESE
S_sp.SUN_WT4_FJQ87_RS02760                                  465  HGLRG........VKGLDTKLGTN..IHFILSASDSSLLNQHAEINNTAQILRD.AE.
S_sp.MBTL60-112-B2_K5Q73_RS09520                            465  HGVRG........TDGLDTPLGAN..IKFILSASDSSLLNQHAEINNTADIFRN.AE.
S_sp.MBTL60_112_B1_K5Q83_RS07010                            465  HGVRG........TDGLDTPLGAN..IKFILSASDSSLLNQHAEINNTADIFRN.AE.
S_enrypsychrophilus_strain_YLB-08_FM038_RS01260             466  HGVRG........TDDLDTPLGTN..IKVIISASDSSLLNQHAEINNTAKILEDETG.
S_enrypsychrophilus_strain_YLB-08_FM038_RS01535             506  DGVLGMT..VDSDTPENDEKYGTN..IKFLMTIAGN.TVNQHGDIEEVKKVLSLEPTP
S_enrypsychrophilus_strain_YLB-08_FM038_RS23490             466  HDVRYSD....ATKMANDDALETN..VRMLWLTSSNMIAGQNADHNRAHRLLENPGE.
S_enrypsychrophilus_strain_YLB-08_FM038_RS23525             453  HDVRYSD....KSLMDADTPLETN..VRFLWLASSNMLANQNGDINRADRLLNDES..
S_sp.YLB_09_FS418_RS01245                                   466  HGVRG........TDDLDTPLGTN..IKVIISASDSSLLNQHAEINNTAKILEDETG.
S_sp.YLB_09_FS418_RS01520                                   506  DGVLGMT..VDSDTPENDEKYGTN..IKFLMTIAGN.TVNQHGDIEEVKKVLSLEPTP
S_sp.YLB_09_FS418_RS02865                                   466  HGVRG........TDDLDTPLGTN..IKVIISASDSSLLNQHAEINNTAKILEDETG.
S_sp.YLB_09_FS418_RS03140                                   506  DGVLGMT..VDSDTPENDEKYGTN..IKFLMTIAGN.TVNQHGDIEEVKKVLSLEPTP
S_sp.YLB_09_FS418_RS25020                                   466  HDVRYSD....ATKMANDDALETN..VRMLWLTSSNMIAGQNADHNRAHRLLENPGE.
S_sp.YLB_09_FS418_RS25055                                   453  HDVRYSD....KSLMDADTPLETN..VRFLWLASSNMLANQNGDINRADRLLNDES..
S_sp._WPAGA9_IGB07_RS19235                                  465  HGITG........TDDLDTPLGTN..IKVIISASDSSLLNQHADINGTAEILKDESG.
S_sp._ARC9_LZ_GUY17_RS19100                                 472  DGVRFTASGSQELFDNNPAKLGTN..IKAIINCAGNALINQHSDSNGTAQILEDDSM.
S_psychromarinicola_strain_M2_EGC80_RS00660                 450  DGVQG.....VDLDDEGDGKLGTD..IKAIINYGGNALINQHSDVRKTEKLLQDEAK.
S_psychromarinicola_strain_M2_EGC80_RS06745                 472  DGVRFKSSGSQERVDNNPAKLGTN..IKAIINCAGNALLNQHSDSNGTAQILQDDSK.
S_psychromarinicola_strain_M2_EGC80_RS11440                 451  DDIILPP..DEIDS.RGDGKLGAN..IKCAW.VRGGNHLGQQGDTFKIAKELKD....
S_sp.Actino-trap-3_CXF80_RS10115                            450  DGVQG.....VDLDDEGDGKLGTD..IKAIINYGGNALINQHSDVRKTEKLLQDEAK.
S_sp.Actino-trap-3_CXF80_RS15635                            472  DGVRFKSSGSQERVDNNPAKLGTN..IKAIINCAGNALLNQHSDSNGTAQILQDDSK.
S_livingstonensis_strain_LMG_19866_EGC82_RS20515            472  DGVRFTASGSQELFDNNPAKLGTN..IKAIINCAGNALINQHSDSNGTAQILEDDSM.
Ferrimonas_lipolytica_strain_S7_HER31_RS14690               444  HGIRG............SDKLSAN..FKFIWNYAGNAMINQHSDTVETDKILQDDTL.
Ferrimonas_lipolytica_strain_S7_HER31_RS15965               494  HGVKIY...NKDGELQRDEKLGTD..IKAVFCSAGNGLINQHCDINYTRPILEDESK.
Ferrimonas_lipolytica_strain_S7_HER31_RS04980               449  DNIRIAD..DDLDA.NGDGVIGQN..TKAIF.VRGGNPFNQQCDSMKIRELLTD....
S_sp.ISTPL2_CCLCJOKE_1_HUB64_RS13995                        465  DGIRG........TTDLDTPLGAD..IKAVFAINSNALINQHSDCNGTAKILEDDTK.
S_japonica_strain_KCTC_22435_SJ2017_RS00815                 465  HGITG........TDDLDTPLGTN..IKVIISASDSSLLNQHADINGTAEILKDESG.
S_sp.8A_M2897_RS06500                                       465  DGIRG........TADLDTPLGAD..IKAVFAINSNALINQHSDCNGTAKILEDDSK.
S_woodyi_ATCC_51908_Swoo_0233                               466  HGVRG........TDGLDTPLGTN..IKVIISASDSSLLNQHAEINNTARILEDETG.
Ferrimonas_balerica_DSM_9799_Fbal_2476                      452  HDVLDS.....QRPGDPTVQLERN..IRAIIVAGSNMLGNQHSNINRTHRLLAAEDS.
Ferrimonas_balerica_DSM_9799_Fbal_3621                      458  DGVKG.........LADGEKLGVN..IKAVFSSSGNALINQHSEINHTRQILEDESK.
S_frigidimarina_NCIMB_400_Sfri_3128                         450  DGVQG.....VDLDEAGDGKLDTD..IKAIINYGGNALINQHSDVRKTEKLLQDETK.
S_frigidimarina_NCIMB_400_Sfri_3684                         466  DGIRG........TADLDTPLGAD..TKAIFAINSNALINQHSDSNGIAKILEDETK.
S_sediminis_HAW-EB3_Ssed_0229                               466  HGVRG........TDGLDTPLGTN..IKVIISASDSSLLNQHAEINNTAKILEDETG.
S_sediminis_HAW-EB3_Ssed_0350                               450  DGVQG.....VDTDEEGDGKLGTD..IKAIINYGGNALINQHSDVRKTEKLLQDESK.
S_sediminis_HAW-EB3_Ssed_0359                               453  HDVRYSN....PDLMAADTPLETN..VRFLWLASSNMLANQNGDINRADRLLNDES..
S_sediminis_HAW-EB3_Ssed_1307                               452  DGVKLPA..EELDE.NGDGKLGTN..IKAIINFASGCLVNQHGDAFGTTRILQD....
S_sediminis_HAW-EB3_Ssed_1404                               442  DGIRG............AEKLSAN..IKFIWNYAGNALINQHSDVNKTHEILQDESL.
S_sediminis_HAW-EB3_Ssed_2923                               451  DGVRG.....VELDENGDGKLGTD..IKAIVNWFSNSMINQHSDVRKSEEVIKDESL.
S_sp.KX20019_JK628_RS15600                                  466  HGVKG........TDGLDTPLGTN..IKVIISASDSTLLNQHAQINNTAEIYQDESG.
S_sp.KX20019_JK628_RS20550                                  450  DGVQG.....VDLDEEGDGKLDTD..IKAIINYGGNALINQHSDVRKTETLLKDDTK.
Ferrimonas_sp.SCSIO_43195_J8Z22_RS00580                     499  DGVKGLD..YDLKDRSKDETYGTN..IKFLMTIAGN.TVNQHGDINEVKRILSIDPSR
Ferrimonas_sp.SCSIO_43195_J8Z22_RS11605                     459  DGVKLP...ADQLDANGDGKLGVN..IKAIVNYAGNALINQHADTKKTEALLKDESK.
Pseudomonas_sp.SCT_BHD08_RS22425(idrA)                      479  IGTTWVA......AMMGSNALEAK..MRSLTAESPHQIKSLDRAAIFETLKARVDSGG
Denitromonas_sp.IR12_I8J34_RS03780(idrA)                    487  IGTTWTA......AMMASQALQDK..MFSLTRGNPHQISSLDRKAIFETLKQRVDSGG
```

```
S_oneidensis_MR-1_dmsA                                  512  ........CELIVVCDCWMTA.SARYADILLPDTTWLES.NDLVNDS..YASGIMGYL
S_oneidensis_MR-1_SO_4358                               545  .......AQDLFILAIDHMFSPSAKMADIILPCITNLEN.SDIVTNKLDHDSGMMVYS
S_sp._LZH-2_JM642_14190                                 512  ........CELIVVCDCWMTP.SARYADILLPDTTWLES.NDLVNDS..YASGIMGYL
S_xiamenensis_NUITM-VS1_NUITMVS1_29540                  512  ........CELIVVCDCWMTP.SARYADILLPDTTWLES.NDLVNDS..YASGIMGYL
S_putrefaciens_strain_FDAARGOS_681_FOB89_20615          512  ........CELIVVCDCWMTP.SARYADILLPDTTWLES.NDLVNDS..YASGIMGYL
S_sp._MR-4_Shewmr4_3676                                 513  ........CELIVVADCWMTP.SARYADILLPDTSWLES.NDLVDGS..YATGLMGYL
S_fidelis_ATCC-BAA-318__L884__L884_RS0114160            511  ........DLFIVSCDCWMTP.GAKFADIILPDTSWLES.NDLVNDS..YASGSLGYL
S_piezotolerans_WP3_SWP_RS15450                         513  ........VELIVSCDCWMTP.GAKFADIILPDTSWLES.NDLVNDS..YASGALGYI
S_schlegeliana_strain_JCM_JMA39_11561_RS09490           511  ........DLFIVSCDCWMTP.GAKFADIILPDTSWLES.NDLVNDS..YASGSLGYL
S_marisflavi_strain_EP1_CFF01_RS01175                   512  ......GGCGLIVVSDCWMTP.SARYADILLPDTTWLES.NDLIDNS..YATGLMGYL
S_sp.SUN_WT4_FJQ87_RS02760                              511  ........DLFVVSCDCWMTP.GAKFADIILPDTSWLES.NDLVDNS..YAAGALGYL
S_sp.MBTL60-112-B2_K5Q73_RS09520                        511  ........DLFIVSCDCWMTP.GAKFADIILPDTSWLES.NDLVNDS..YASGSLGYL
S_sp.MBTL60_112_B1_K5Q83_RS07010                        511  ........DLFIVSCDCWMTP.GAKFADIILPDTSWLES.NDLVNDS..YASGSLGYL
S_enrypsychrophilus_strain_YLB-08_FM038_RS01260         513  ........VELIVSCDCWMTP.GAKFADIILPDTSWLES.NDLVNDS..YASGALGYI
S_enrypsychrophilus_strain_YLB-08_FM038_RS01535         559  .GQKEEPGLDMHVVIDTQMTP.TAQWADIVLPDTTWTER.YDYIASD..N......TT
S_enrypsychrophilus_strain_YLB-08_FM038_RS23490         517  .......YIDHVVVFEQHMTG.SAKYADILLPEISWLEM.YDVIQSVSKYDDGSLRYI
S_enrypsychrophilus_strain_YLB-08_FM038_RS23525         503  .......LVDFVVVVEQHMTA.SAKYADIILPEVSWLEM.YDVIQSYNKMDCGSLMYA
S_sp.YLB_09_FS418_RS01245                               513  ........VELIVSCDCWMTP.GAKFADIILPDTSWLES.NDLVNDS..YASGALGYI
S_sp.YLB_09_FS418_RS01520                               559  .GQKEEPGLDMHVVIDTQMTP.TAQWADIVLPDTTWTER.YDYIASD..N......TT
S_sp.YLB_09_FS418_RS02865                               513  ........VELIVSCDCWMTP.GAKFADIILPDTSWLES.NDLVNDS..YASGALGYI
S_sp.YLB_09_FS418_RS03140                               559  .GQKEEPGLDMHVVIDTQMTP.TAQWADIVLPDTTWTER.YDYIASD..N......TT
S_sp.YLB_09_FS418_RS25020                               517  .......YIDHVVVFEQHMTG.SAKYADILLPEISWLEM.YDVIQSVSKYDDGSLRYI
S_sp.YLB_09_FS418_RS25055                               503  .......LVDFVVVVEQHMTA.SAKYADIILPEVSWLEM.YDVIQSYNKMDCGSLMYA
S_sp._WPAGA9_IGB07_RS19235                              512  ........VELIVSCDCWMTP.GAKFADIIIPDTSWLES.NDLVDDS..YASGAMGYI
S_sp._ARC9_LZ_GUY17_RS19100                             527  ........CELIIVSDCWMTS.SAKFADYLLPDSTWLES.VDYANDS..YASGQMAYA
S_psychromarinicola_strain_M2_EGC80_RS00660             500  ........CEFILVVDNWMTP.SAKFADILLPDVSWLES.EDLVNQS..YAAGDTASL
S_psychromarinicola_strain_M2_EGC80_RS06745             527  ........CELIIVSDCWMTS.SAKFADYLLPDSTWLES.VDYANDS..YASGQMAYA
S_psychromarinicola_strain_M2_EGC80_RS11440             499  .....QTNVELIINIENHMSS.TAMYSDYVLPEMTWYEQ.TDLPWSN..LQSGSMPFV
S_sp.Actino-trap-3_CXF80_RS10115                        500  ........CEFILVVDNWMTP.SAKFADILLPDVSWLES.EDLVNQS..YAAGDTASL
S_sp.Actino-trap-3_CXF80_RS15635                        527  ........CELIIVSDCWMTS.SAKFADYLLPDSTWLES.VDYANDS..YASGQMAYA
S_livingstonensis_strain_LMG_19866_EGC82_RS20515        527  ........CELIIVSDCWMTS.SAKFADYLLPDSTWLES.VDYANDS..YASGQMAYA
Ferrimonas_lipolytica_strain_S7_HER31_RS14690           487  ........CEFILVHDVQMTP.SAKYADILLPDFTDAEV.NDISAN....SGTDSGVL
Ferrimonas_lipolytica_strain_S7_HER31_RS15965           546  ........AELIVVTDCWLTP.SAEIADYVLPDSTWMES.NDIADDS..YSSGETGYQ
Ferrimonas_lipolytica_strain_S7_HER31_RS04980           497  .....PAEVELIVTIDTHLTP.SMYVNDYILPDSTMYER.EDFPATN..MDNGAMGFL
S_sp.ISTPL2_CCLCJOKE_1_HUB64_RS13995                    512  ........CELIVVCDCWMTA.SARYADILLPDTTWLES.NDLVNDS..YASGIMGYL
S_japonica_strain_KCTC_22435_SJ2017_RS00815             512  ........VELIVSCDCWMTP.GAKFADIIIPDTSWLES.NDLVDDS..YASGAMGYI
S_sp.8A_M2897_RS06500                                   512  ........CELIVVCDCWMTP.SARYADILLPDTTWLES.NDLVNDS..YASGIMGYL
S_woodyi_ATCC_51908_Swoo_0233                           513  ........VELIVSCDCWMTP.GAKFADIILPDTSWLES.NDLVNDS..YASGALGYI
Ferrimonas_balerica_DSM_9799_Fbal_2476                  502  .......KVDFVLVSEIYMTP.TARFADILLPEITQFET.EDLVCDG..WHSGDMANL
Ferrimonas_balerica_DSM_9799_Fbal_3621                  504  ........CELIVVTDCWMTA.SARFADYILPDTTWVES.NDLANDS..YASGETGYL
S_frigidimarina_NCIMB_400_Sfri_3128                     500  ........CEFILVVDNWMTP.SAKFADVLLPDVSWLES.EDLVNQS..YAAGDTASL
S_frigidimarina_NCIMB_400_Sfri_3684                     513  ........CELIVVCDCWMTS.SAKFADILLPDTSWLES.NDLVNDS..YASGIMGYL
S_sediminis_HAW-EB3_Ssed_0229                           513  ........VELIVSCDCWMTP.GAKFADIILPDTSWLES.NDLVNDS..YASGALGYI
S_sediminis_HAW-EB3_Ssed_0350                           500  ........CEFILVVDNWMTP.SAKFADVLLPDVTWLES.EDLIYQS..YAAGDTATL
S_sediminis_HAW-EB3_Ssed_0359                           503  .......LVDFVVVVEQHMTA.SAKYADIILPEVSWLEM.YDVIQSYNKMDCGSLMYA
S_sediminis_HAW-EB3_Ssed_1307                           501  .....ESKCEMIVVIDNHMTS.SAKLADYILPDNTWLES.TDFANKT..YYAGAVSYM
S_sediminis_HAW-EB3_Ssed_1404                           485  ........CEFILVHDVQMTP.SAKYADILLPDLTDAEG.TDISAN....SATEAGVL
S_sediminis_HAW-EB3_Ssed_2923                           501  ........CEFILTVDNIMTP.SAKFSDIILPDTTWLED.EELVNQN..HVPGDTGTV
S_sp.KX20019_JK628_RS15600                              513  ........VELIVSCDCWMTP.GAKFADIILPDTSWLES.NDLVNDS..YASGALGYI
S_sp.KX20019_JK628_RS20550                              500  ........CEFILVVDNWMTP.SAKFADILLPDVSWLES.EDLVNQS..YAAGDTASL
Ferrimonas_sp.SCSIO_43195_J8Z22_RS00580                 552  SGDPTEPGLDMHVVIDTQMTP.TAQWADIIIPDTTWTER.FDFICSD..N......AT
Ferrimonas_sp.SCSIO_43195_J8Z22_RS11605                 511  ........CEFIVVIDNWMTP.SAEFADILLPDINWLETKDDLINQS..YAAGDTATL
Pseudomonas_sp.SCT_BHD08_RS22425(idrA)                  529  ........MVMANSDLYPVVPVGTDFADIVYPVASWGED..NFTRCN.......SERR
Denitromonas_sp.IR12_I8J34_RS03780(idrA)                537  ........TVIANSDIYPCVPVGTEYADIVLPAATWGED..NFTRCN.......SERR
```

```
S_oneidensis_MR-1_dmsA                                558  VAMKAAVKPMWE.SISMFEMGRLIAAKMG.KEAEYTEGKDEA.AWLEELYQKTRTNSA
S_oneidensis_MR-1_SO_4358                             595  YVFERAIEPMYE.AKSTWEICRLLAARMG.VEHSYA.RLKTQDEWVKWSFEHYAKKVP
S_sp._LZH-2_JM642_14190                               558  VAMKAAVKPMWE.SISMFEMGRLIAAKMG.KEAEYTEGKDEA.AWLEELYQKTRTNSA
S_xiamenensis_NUITM-VS1_NUITMVS1_29540                558  VAMKAAVKPMWE.SISMFEMGRLIAAKMG.KEAEYTEGKDEA.AWLEELYQKTRTNSA
S_putrefaciens_strain_FDAARGOS_681_FOB89_20615        558  VAMKAAVKPMWE.SISMFEMGRLIAAKMG.KEAEYTEGKDEA.AWLEALYQKTRTDSS
S_sp._MR-4_Shewmr4_3676                               559  VAMKAAVKPMWE.CKSMYDMCGLIAQKMG.VYQQYSEGKDET.AWLNQLYAGTLTAPS
S_fidelis_ATCC-BAA-318__L884__L884_RS0114160          557  TAMSSSVESMWD.CKSMYEAAAMIADKMG.VGAAFRDGKNEE.EWLEELYQSTKANSN
S_piezotolerans_WP3_SWP_RS15450                       559  TAMKSAIEPMWD.CKSMYEASAMIAKYMG.CEAEFTEGKTEE.QWLEELYQKTKDSST
S_schlegeliana_strain_JCM_JMA39_11561_RS09490         557  TAMSSSVETMWD.CKSMYEAAAMIADKMG.VGAGFRDGKNED.EWLEQLYQSTKANSN
S_marisflavi_strain_EP1_CFF01_RS01175                 560  VAMKAAVDPLWD.CKSMYDMCGLIAQKMG.VYAQYSEGKDEA.AWLNQLYAETLTKPT
S_sp.SUN_WT4_FJQ87_RS02760                            557  TAMSAAVEPMWD.CKSMHDAAAMIADKMG.VGPQFTEGKTEA.QWLEELYQKTRVMSQ
S_sp.MBTL60-112-B2_K5Q73_RS09520                      557  TAMSSSVETMWD.CKSMYEAAAMIADKMG.VGAGFRDGKNED.EWLEELYQSTKANSN
S_sp.MBTL60_112_B1_K5Q83_RS07010                      557  TAMSSSVETMWD.CKSMYEAAAMIADKMG.VGAGFRDGKNED.EWLEELYQSTKANSN
S_enrypsychrophilus_strain_YLB-08_FM038_RS01260       559  TAMKSAIEPMWD.CKSMYEAAALIAKYMG.CEAEFTEGKTEE.QWLEELYQKTKDNSN
S_enrypsychrophilus_strain_YLB-08_FM038_RS01535       606  VYTVPSIEPIGD.SKPVFEFLVMLAEEMG.VTGFATKGLTQEAMTKTFIDEAVNGPMG
S_enrypsychrophilus_strain_YLB-08_FM038_RS23490       566  YGIEPAMEPMFE.CLHSFDICRGIARHLG.TEGKFE.GGKTYKQWVEDIFKNVKKNNP
S_enrypsychrophilus_strain_YLB-08_FM038_RS23525       552  YGITPAIDPMFE.CKPAYDICHMIANELG.IGSKFTNGGMTYLDQVRKVFAEFKKSNS
S_sp.YLB_09_FS418_RS01245                             559  TAMKSAIEPMWD.CKSMYEAAALIAKYMG.CEAEFTEGKTEE.QWLEELYQKTKDNSN
S_sp.YLB_09_FS418_RS01520                             606  VYTVPSIEPIGD.SKPVFEFLVMLAEEMG.VTGFATKGLTQEAMTKTFIDEAVNGPMG
S_sp.YLB_09_FS418_RS02865                             559  TAMKSAIEPMWD.CKSMYEAAALIAKYMG.CEAEFTEGKTEE.QWLEELYQKTKDNSN
S_sp.YLB_09_FS418_RS03140                             606  VYTVPSIEPIGD.SKPVFEFLVMLAEEMG.VTGFATKGLTQEAMTKTFIDEAVNGPMG
S_sp.YLB_09_FS418_RS25020                             566  YGIEPAMEPMFE.CLHSFDICRGIARHLG.TEGKFE.GGKTYKQWVEDIFKNVKKNNP
S_sp.YLB_09_FS418_RS25055                             552  YGITPAIDPMFE.CKPAYDICHMIANELG.IGSKFTNGGMTYLDQVRKVFAEFKKSNS
S_sp._WPAGA9_IGB07_RS19235                            558  TAMKAAVDPIWD.CKSMYQVAALIAEKMG.VGAEFTEGKTED.EWLEELYQQTRENSK
S_sp._ARC9_LZ_GUY17_RS19100                           573  TFMSTSLKPLGE.SQSMYEICAGIANAMG.NGAEYTEGLNGQ.QWCEKLYQQTKAMNT
S_psychromarinicola_strain_M2_EGC80_RS00660           546  VQMSTGVDPMFE.SRPIYEVCVDLARRMG.VESEFTEGKSRK.EWLDQFYAESKAATP
S_psychromarinicola_strain_M2_EGC80_RS06745           573  TFMSTSLKPLGE.SQSMYEICAGIANAMG.NGAAYTEGLTGE.QWCEKLYQETKSMNA
S_psychromarinicola_strain_M2_EGC80_RS11440           548  IAT.TGIPRIGD.TKSTWEICEGIAKVLG.IESEFSMGMTP.TQVLEHGYQVLRMF..
S_sp.Actino-trap-3_CXF80_RS10115                      546  VQMSTGVDPMFE.SRPIYEVCVDLARRMG.VESEFTEGKSRK.EWLDQFYAESKAATP
S_sp.Actino-trap-3_CXF80_RS15635                      573  TFMSTSLKPLGE.SQSMYEICAGIANAMG.NGAAYTEGLTGE.QWCEKLYQETKSMNA
S_livingstonensis_strain_LMG_19866_EGC82_RS20515      573  TFMSTSLKPLGE.SQSMYEICAGIANAMG.NGAEYTEGLNGQ.QWCEKLYQQTKAMNT
Ferrimonas_lipolytica_strain_S7_HER31_RS14690         531  TAMTTSIDTPFD.PKGCFEVANEVAKRLG.VEAAFNEGRTYQ.EWLEYLYAASTAGGD
Ferrimonas_lipolytica_strain_S7_HER31_RS15965         592  TYMSSSLKPFFESTKSMYDIGLGIVKASGGDVSTYTDGKADASEWLDELYEQTKAKSQ
Ferrimonas_lipolytica_strain_S7_HER31_RS04980         546  IAT.QGIPPIGN.VKSTFEICLEIAKKMG.IDELYSSGCSNDEEALVATYNFTRMIRP
S_sp.ISTPL2_CCLCJOKE_1_HUB64_RS13995                  558  VAMKAAVKPMWE.SISMFEMGRLIAAKIG.KEAEYTEGKDEA.AWLEALYQKTRTDSS
S_japonica_strain_KCTC_22435_SJ2017_RS00815           558  TAMKAAVDPIWD.CKSMYQVAALIAEKMG.VGAEFTEGKTED.EWLEELYQQTRENSK
S_sp.8A_M2897_RS06500                                 558  VAMKAAVKPMWE.SISMFEMGRLIAAKMG.KEAEYTEGKDEA.AWLEELYQKTRTNSA
S_woodyi_ATCC_51908_Swoo_0233                         559  TAMKSAIEPMWD.CKSMYEAAALIAKYMG.CEAEFTEGKTEE.QWLEELYQKTKDNSN
Ferrimonas_balerica_DSM_9799_Fbal_2476                549  LATTAAIAPRGE.SRSAYDICADIAERFG.IRQTFTEGRTKA.EWVRHLWQTPQASSS
Ferrimonas_balerica_DSM_9799_Fbal_3621                550  TFMSSSLDPLYN.CRNLYQIGLGLAKVFG.KEAEYTEGRSEQ.EWLDHLYQGTVS..S
S_frigidimarina_NCIMB_400_Sfri_3128                   546  VQMSSSVDPMFE.SRPIYEVCVDLARRMG.VEAEFTEGKSRK.DWLDQFYAESKEATP
S_frigidimarina_NCIMB_400_Sfri_3684                   559  VAMKAAIKPLWE.CKSMYDMCALIAEKMG.KLGEYTEGKDEA.GWLTELYEKTRTNSN
S_sediminis_HAW-EB3_Ssed_0229                         559  TAMKSAIEPMWD.CKSMYEASALIAKYMG.CEAEFTEGKTEE.QWLEELYQKTKDSST
S_sediminis_HAW-EB3_Ssed_0350                         546  VQMSSGVDPMFE.SRPIYEVCVDLAKRMG.VEAEFTEGKSRK.DWLDQFYAESKAATP
S_sediminis_HAW-EB3_Ssed_0359                         552  YGITPAVDPMFE.CKPAYDICHMIANQLG.IGSKFTNGGMTYLDQVRKVFAEFKKSNP
S_sediminis_HAW-EB3_Ssed_1307                         550  TALKTGIKPLGE.CRGSFDVCTDLAEKMG.VKEIFTEGRTTDEWIEHYYQTKVRTLDP
S_sediminis_HAW-EB3_Ssed_1404                         529  TAMTSSVNTPFS.AKGCFEVCLEIAKRLG.VEQEYAEGKNYQ.QWLESLYNEVAVKKG
S_sediminis_HAW-EB3_Ssed_2923                         547  IQMSSGIEPLFE.SRHSYDICTEIAKRMG.CEQAFTEGKTMQ.QWLNEMYAMSQAANP
S_sp.KX20019_JK628_RS15600                            559  TAMKSAIEPMWD.CKSMYEASALIAKYMG.CEAEFTEGKTES.EWLEELYQKTRDSSK
S_sp.KX20019_JK628_RS20550                            546  VQMSSGVEPMFE.SRPIYEVCVDLAKRMG.VEAEFTEGKSRK.DWLDQFYAESKAATP
Ferrimonas_sp.SCSIO_43195_J8Z22_RS00580               600  VYTVPSIEPEGD.CKPAFEFMVMLADQFG.LSGFASKGRTQEAMTKTFIDEAINGPMG
Ferrimonas_sp.SCSIO_43195_J8Z22_RS11605               558  VQMSSSLEPMFE.CRDAYDIALGIAEIWG.VAGQFTEGKTKQ.QWRDELYAVAKNATP
Pseudomonas_sp.SCT_BHD08_RS22425(idrA)                570  LRLYSKFYDAPGEAKPDWWIVQKFAQKMG.LDKDGGYSWKDSNDVFEEVARFSRDGVL
Denitromonas_sp.IR12_I8J34_RS03780(idrA)              578  LRLYSKFYDAPGEAKPDWWIIAKFAQKMG.YDKDGSYQWKNSNDVFEEAARFGRNGVL
```

**S_oneidensis_MR-1_dmsA**	**613**	NTDAQPAFPNSYAEAQKIGVFRAFSKTP..VLALDSYING...........GKAVSTP
**S_oneidensis_MR-1_SO_4358**	**650**	NL...YNPEDTYESVLGRSPVRRIASENPLFKGLREFK...........YGGVTLNTP
**S_sp._LZH-2_JM642_14190**	**613**	NTDAQPAFPNSYAEAQKIGVFRAFSKTP..VLALDSYING...........GKAVSTP
**S_xiamenensis_NUITM-VS1_NUITMVS1_29540**	**613**	NTDAQPAFPNSYAEAQKIGVFRAFSKTP..VLALDSYING...........GKAVSTP
**S_putrefaciens_strain_FDAARGOS_681_FOB89_20615**	**613**	NVNAQPSFPSTYTEAQQIGVFRAFSKTP..VLALDSYING...........GKAVSTP
**S_sp._MR-4_Shewmr4_3676**	**614**	SANAIPALPSSYEEAQKIGVFRSFNGTK..RIALDSYING...........GKALGTP
**S_fidelis_ATCC-BAA-318__L884__L884_RS0114160**	**612**	NINVSPAFPATYKEAQKIGFFRKNMNDN..HVSLQSFVKD...........GKPLSTP
**S_piezotolerans_WP3_SWP_RS15450**	**614**	NLGVVPAFPATYKEAQEIGFFRKHMQDD..HVALSSYING...........GEPLATP
**S_schlegeliana_strain_JCM_JMA39_11561_RS09490**	**612**	NIGVSPAFPATYKEAQKIGFFRKNMNDN..HVALQSFVKE...........GKALSTP
**S_marisflavi_strain_EP1_CFF01_RS01175**	**615**	SAAAIPALPLSYEEAQKIGVFRSFNGTK..RLALDSYING...........GQPLRTP
**S_sp.SUN_WT4_FJQ87_RS02760**	**612**	NQGVVPPFPATYKEAQAQGLFRKDMQLN..HVALADFVHE...........GKALSTP
**S_sp.MBTL60-112-B2_K5Q73_RS09520**	**612**	NIGVSPAFPATYKEAQEIGFFRKNMNDN..HVALQSFVKE...........GKALSTP
**S_sp.MBTL60_112_B1_K5Q83_RS07010**	**612**	NIGVSPAFPATYKEAQEIGFFRKNMNDN..HVALQSFVKE...........GKALSTP
**S_enrypsychrophilus_strain_YLB-08_FM038_RS01260**	**614**	NLGVVPAFPATYKEAQEVGFFRKNMNDN..HVALESYIKG...........GKALSTP
**S_enrypsychrophilus_strain_YLB-08_FM038_RS01535**	**662**	DPT..LPHWSDHEEHGYVFTHKFEEPSIG.MEKEFNQ...........RGGGVKLSTP
**S_enrypsychrophilus_strain_YLB-08_FM038_RS23490**	**621**	.....WLKGDSFDEFVKVGPQREEAPEHPNLQCLRDFINTNPRHPEFDNTTEGALSTP
**S_enrypsychrophilus_strain_YLB-08_FM038_RS23525**	**608**	.....WIQGDTFEEFVSFGPQRRPAGEHPNVGKLRDFV.TDP........VNNPLGTP
**S_sp.YLB_09_FS418_RS01245**	**614**	NLGVVPAFPATYKEAQEVGFFRKNMNDN..HVALESYIKG...........GKALSTP
**S_sp.YLB_09_FS418_RS01520**	**662**	DPT..LPHWSDHEEHGYVFTHKFEEPSIG.MEKEFNQ...........RGGGVKLSTP
**S_sp.YLB_09_FS418_RS02865**	**614**	NLGVVPAFPATYKEAQEVGFFRKNMNDN..HVALESYIKG...........GKALSTP
**S_sp.YLB_09_FS418_RS03140**	**662**	DPT..LPHWSDHEEHGYVFTHKFEEPSIG.MEKEFNQ...........RGGGVKLSTP
**S_sp.YLB_09_FS418_RS25020**	**621**	.....WLKGDSFDEFVKVGPQREEAPEHPNLQCLRDFINTNPRHPEFDNTTEGALSTP
**S_sp.YLB_09_FS418_RS25055**	**608**	.....WIQGDTFEEFVSFGPQRRPAGEHPNVGKLRDFV.TDP........VNNPLGTP
**S_sp._WPAGA9_IGB07_RS19235**	**613**	NLGVSPAFPSTYKEAQEIGFFRKNMENR..NVALQGFVNG...........GEALGTP
**S_sp._ARC9_LZ_GUY17_RS19100**	**628**	DV....DMPDTYEEAQKVGVFRKYMSAS..VVAFESYIQDP.........VANPRPTI
**S_psychromarinicola_strain_M2_EGC80_RS00660**	**601**	ELP.......AKEQMLTQGIYRKYLPNGG.YIVLEDFRTDP.........DANPLGTP
**S_psychromarinicola_strain_M2_EGC80_RS06745**	**628**	DV....DMPDTYQEAQKVGVFRKYMSSP..VIAFQSYIQDP.........IANPRPTI
**S_psychromarinicola_strain_M2_EGC80_RS11440**	**600**	..H..PELPSTLSEMQEKQIFKFANPCDD.SESSAIRHCAMY...EYVTNG.GLLNTA
**S_sp.Actino-trap-3_CXF80_RS10115**	**601**	ELP.......EKEQMLTQGIYRKYLPNGG.YIVLEDFRTDP.........DANPLGTP
**S_sp.Actino-trap-3_CXF80_RS15635**	**628**	DV....DMPDTYQEAQKVGVFRKYMSSP..VIAFQSYIQDP.........IANPRPTI
**S_livingstonensis_strain_LMG_19866_EGC82_RS20515**	**628**	DV....DMPDTYEEAQKVGVFRKYMSAS..VVAFESYIQDP.........VANPRPTI
**Ferrimonas_lipolytica_strain_S7_HER31_RS14690**	**586**	YP........TYEELVEQGIYRLKNPVQ..VIGLKSFIDDP.........DINPLSTP
**Ferrimonas_lipolytica_strain_S7_HER31_RS15965**	**650**	NATLN..LPATYAEAQAKGFFRGHAPDRG.PLTLESFVNE...........GAALSTT
**Ferrimonas_lipolytica_strain_S7_HER31_RS04980**	**601**	DLH..PYMPETWAEMKEKQMVKWARPVED.TDESARWHVGYW...DYVQYGTEPLNTP
**S_sp.ISTPL2_CCLCJOKE_1_HUB64_RS13995**	**613**	NVNAQPAFPSTYTEAQQIGVFRAFSKTP..VLALDSYINN...........GKAVSTP
**S_japonica_strain_KCTC_22435_SJ2017_RS00815**	**613**	NLGVSPAFPSTYKEAQEIGFFRKNMENR..NVALQGFVNG...........GEALGTP
**S_sp.8A_M2897_RS06500**	**613**	NTDAQPAFPNSYAEAQKIGVFRAFSKTP..VLALDSYING...........GKAVSTP
**S_woodyi_ATCC_51908_Swoo_0233**	**614**	NLGVYPAFPATYKEAQEIGFFRKNMNDK..HVALESYIKG...........GKPLSTP
**Ferrimonas_balerica_DSM_9799_Fbal_2476**	**604**	LSLP......DFDEAKAHGVYREYLPAS..KPGFADFRADP.........QANPLKTP
**Ferrimonas_balerica_DSM_9799_Fbal_3621**	**603**	NPELN..LPATYSEAQKVGFYRRHAPDT..HVALKDFVKD...........GKALSTP
**S_frigidimarina_NCIMB_400_Sfri_3128**	**601**	ELP.......AKEVMLTQGIYRKYLPNGG.YIVLEDFRTDP.........DANPLGTP
**S_frigidimarina_NCIMB_400_Sfri_3684**	**614**	NANAIPPLPSTYEEAQAQGVFRAFDGKT..VVALEDYING...........TDTLNTP
**S_sediminis_HAW-EB3_Ssed_0229**	**614**	NLGVVPAFPATYKEAQEIGFFRKNMNDN..HVALKSYING...........GKALSTP
**S_sediminis_HAW-EB3_Ssed_0350**	**601**	GLP.......DKEVMLTQGIYRKYLPDGG.YIVLEDFRNDP.........EANPLGTP
**S_sediminis_HAW-EB3_Ssed_0359**	**608**	.....WIQGDTFEEFVSFGPQRRPAGEHPNVGKIRDFI.TDP........IASPLGTP
**S_sediminis_HAW-EB3_Ssed_1307**	**606**	.....ELFPATAKEMQDIGVVKTFSPCDD.SEESAKVNCAMW...DYVQNG.GTLKTA
**S_sediminis_HAW-EB3_Ssed_1404**	**584**	YP........NYDELVKMGIYRVKDTKP..VIGLKSYIDDP.........VASPLSTP
**S_sediminis_HAW-EB3_Ssed_2923**	**602**	GLP.......DKEVMLKQGIYRKYLPHGS.QIAMKGFRDDP.........EANRLYTP
**S_sp.KX20019_JK628_RS15600**	**614**	NAGVVPAFPATYKEAQEVGFFRKNMNDN..YVALESYIKG...........GEALSTP
**S_sp.KX20019_JK628_RS20550**	**601**	ELP.......EKEVMLTQGIYRKYLPNGG.YIVLEDFRTDP.........DANPLGTP
**Ferrimonas_sp.SCSIO_43195_J8Z22_RS00580**	**656**	DPT..LPHWSDHEQHGYVFTHAFDEPTIG.MEKEFNP...........RKGMVACSTP
**Ferrimonas_sp.SCSIO_43195_J8Z22_RS11605**	**613**	ELP.......SKEEMKTQGIFRKYLPNGS.YVVLEDFRKDP.........VANPLKTP
**Pseudomonas_sp.SCT_BHD08_RS22425(idrA)**	**627**	NYHP.LAEKAKASGIKAQELLRGYGTTGIQTPIRERGGELVG.........TVRLHDP
**Denitromonas_sp.IR12_I8J34_RS03780(idrA)**	**635**	NYHP.LVVKAKEKGVKGHELLRTYGTDGIQTPIRMKDGELVG.........TQRLHDP

```
S_oneidensis_MR-1_dmsA                                  658  SGKVEIYSLSMAQKGKDWIFNPN.....VKGDYITAIP.MYQPTWEGYD......DE
S_oneidensis_MR-1_SO_4358                               694  SKKIEAYSLTLLNRTRFEKFEET..........ETSFIGKYIPATEGAE........
S_sp._LZH-2_JM642_14190                                 658  SGKVEIYSLSMAQKGKDWIFNPN.....VKGDYITAIP.MYQPTWEGYD......DE
S_xiamenensis_NUITM-VS1_NUITMVS1_29540                  658  SGKVEIYSLSMAQKGKDWIFNPN.....VKGDYITAIP.MYQPTWEGYD......DE
S_putrefaciens_strain_FDAARGOS_681_FOB89_20615          658  SGKIEIYSLSMAQKGKDWIFNPN.....VKGDYISAIP.MYQATWEGYD......DE
S_sp._MR-4_Shewmr4_3676                                 659  TGKVEFYSLDFAQKAAEWEFDAS.....VKGNYITALP.KYQPTWEGYE......DA
S_fidelis_ATCC-BAA-318__L884__L884_RS0114160            657  SGKIEIYSAELAWRAANWDQENSAQNPTGVKGDTITAIP.QYTVTWDGYE......DE
S_piezotolerans_WP3_SWP_RS15450                         659  SGKIEIYSAELAWRAANWDQENSPLNPTGIKGDTITAIP.QYTVTWDGFE......DE
S_schlegeliana_strain_JCM_JMA39_11561_RS09490           657  SGKIEIYSAELAWRAANWDQENSAQNPTGVKGDTITAIP.QYTVTWHGYE......DE
S_marisflavi_strain_EP1_CFF01_RS01175                   660  SGKLEIYSLDFAQKAAEWEFNSE.....VKGDSITALP.KYQATWEGYE......DE
S_sp.SUN_WT4_FJQ87_RS02760                              657  SGKIEIYSAELAWRAANWNTEDK...TGVKGDTITAIP.QYTVTWDGYE......DA
S_sp.MBTL60-112-B2_K5Q73_RS09520                        657  SGKIEIYSAELAWRAANWDQES....ETKVKGDTITAIP.QYTVTWDGYE......DE
S_sp.MBTL60_112_B1_K5Q83_RS07010                        657  SGKIEIYSAELAWRAANWDQES....ETKVKGDTITAIP.QYTVTWDGYE......DE
S_enrypsychrophilus_strain_YLB-08_FM038_RS01260         659  SGKIEIYSAELAWRAANWDQEST...TDVKGDTITAIP.QYTVTWDGYE......DE
S_enrypsychrophilus_strain_YLB-08_FM038_RS01535         706  STLVEAYSPRLAWIASD.KGWDKDEE...YVDGGVTALP.KYLRTWEDFE......DQ
S_enrypsychrophilus_strain_YLB-08_FM038_RS23490         674  SGKIEIYSQTFAAKIKHAAEGDT..........ITAIP.VYDACEESYE........
S_enrypsychrophilus_strain_YLB-08_FM038_RS23525         652  SGKVEIYSQYFESKQSQAEGTDE..........INPLP.VYFACDESYE........
S_sp.YLB_09_FS418_RS01245                               659  SGKIEIYSAELAWRAANWDQEST...TDVKGDTITAIP.QYTVTWDGYE......DE
S_sp.YLB_09_FS418_RS01520                               706  STLVEAYSPRLAWIASD.KGWDKDEE...YVDGGVTALP.KYLRTWEDFE......DQ
S_sp.YLB_09_FS418_RS02865                               659  SGKIEIYSAELAWRAANWDQEST...TDVKGDTITAIP.QYTVTWDGYE......DE
S_sp.YLB_09_FS418_RS03140                               706  STLVEAYSPRLAWIASD.KGWDKDEE...YVDGGVTALP.KYLRTWEDFE......DQ
S_sp.YLB_09_FS418_RS25020                               674  SGKIEIYSQTFAAKIKHAAEGDT..........ITAIP.VYDACEESYE........
S_sp.YLB_09_FS418_RS25055                               652  SGKVEIYSQYFESKQSQAEGTDE..........INPLP.VYFACDESYE........
S_sp._WPAGA9_IGB07_RS19235                              658  SGKIEIHSAELAWRAANWEQNK......VKGDTITAIP.QYTVTWDGYE......DE
S_sp._ARC9_LZ_GUY17_RS19100                             671  SGKFEIFSISLARKAATWSLPTG........DQITPLP.QYVVTWDGYQ......DQ
S_psychromarinicola_strain_M2_EGC80_RS00660             642  SGKIEIYSSRLAEKARTWKLKEG........DVISALP.KYVPTWEGYE......DT
S_psychromarinicola_strain_M2_EGC80_RS06745             671  SGKFEIFSLSLARKAATWSLPTG........DEITPLP.QYVVTWDGYQ......DQ
S_psychromarinicola_strain_M2_EGC80_RS11440             649  SGKIDIYSHKLRELAEKNQIPDYWR....STDYINPIA.KYMVVPGGYE......DS
S_sp.Actino-trap-3_CXF80_RS10115                        642  SGKIEIYSSRLAEKARTWKLKEG........DVISALP.KYVPTWEGYE......DT
S_sp.Actino-trap-3_CXF80_RS15635                        671  SGKFEIFSLSLARKAATWSLPTG........DEITPLP.QYVVTWDGYQ......DQ
S_livingstonensis_strain_LMG_19866_EGC82_RS20515        671  SGKFEIFSISLARKAATWSLPTG........DQITPLP.QYVVTWDGYQ......DQ
Ferrimonas_lipolytica_strain_S7_HER31_RS14690           625  SGKVEVYSETMQTMADTWILPDG........DEIPAIP.KYISTWEGYE......DK
Ferrimonas_lipolytica_strain_S7_HER31_RS15965           694  TGKIEIFSFKWAIDNPRRDTFVADDRDASTHVDMIDPTP.KYVPHWQGFE......DD
Ferrimonas_lipolytica_strain_S7_HER31_RS04980           653  TGKIDIYSHDLQEYSEKSEIPEYWPK...NSGGYINATP.KFMIGPESYL......DS
S_sp.ISTPL2_CCLCJOKE_1_HUB64_RS13995                    658  SGKIEIYSLSMAQKGKDWVFNPN.....VKGDYISAIP.MYQATWEGYD......DE
S_japonica_strain_KCTC_22435_SJ2017_RS00815             658  SGKIEIHSAELAWRAANWEQNK......VKGDTITAIP.QYTVTWDGYE......DE
S_sp.8A_M2897_RS06500                                   658  SGKVEIYSLSMAQKGKDWIFNPN.....VKGDYITAIP.MYQPTWEGYD......DE
S_woodyi_ATCC_51908_Swoo_0233                           659  SGKIEIYSAELAWRAANWDQESK...TQVKGDTITAIP.QYTVTWDGYE......DT
Ferrimonas_balerica_DSM_9799_Fbal_2476                  645  SGKIEIYSEAIANLTRDWDLPEG........DEVTALP.EFRAPWEGGV........
Ferrimonas_balerica_DSM_9799_Fbal_3621                  646  SGKIEIYSLDWAKK..RAEWIPASDKD....YDQITPLP.HYTEAWQGFE......DT
S_frigidimarina_NCIMB_400_Sfri_3128                     642  SGKIEIYSSRLAEKARTWKLKEG........DVISALP.KYVPTWEGYE......DT
S_frigidimarina_NCIMB_400_Sfri_3684                     659  SGKIEIFSLELAQKGTDWEFDST.....VKGNYITSIP.MYQATWEGYE......DS
S_sediminis_HAW-EB3_Ssed_0229                           659  SGKIEIYSAELAWRAANWDQELT...TDVKGDTITAIP.QYTVTWDGYE......DE
S_sediminis_HAW-EB3_Ssed_0350                           642  SGKIEIYSSRLADKARTWKLKEG........DVISALP.KYVPTWEGYE......DT
S_sediminis_HAW-EB3_Ssed_0359                           652  SGKVEIYSQYFEGKQTQAEGTDE..........INPLP.VYFACDESYE........
S_sediminis_HAW-EB3_Ssed_1307                           654  SGKLEIFSHALQELSDLWEVPDYWLD....KDNYLNAIP.KYWVGLESYQ......DP
S_sediminis_HAW-EB3_Ssed_1404                           623  SGKIEVYSETLDTMSKTWTLNEG........DEIPAIP.KYIKTWEGFE......DL
S_sediminis_HAW-EB3_Ssed_2923                           643  SGKIEIYSSRLAHKAKTWELREG........DVISALP.KYTPTWDGYE......DS
S_sp.KX20019_JK628_RS15600                              659  SGKIEIYSAELAWRAANWDQENSPLNPTGVKGDTITAIP.QYTVTWDGFE......DE
S_sp.KX20019_JK628_RS20550                              642  SGKIEIYSSRLAEKARTWKLKEG........DVISALP.KYVPTREGYE......DT
Ferrimonas_sp.SCSIO_43195_J8Z22_RS00580                 700  SGLVETYSPKLAWIASE.DGWDRDPS...YPDGGITALP.KYFTTWEGFE......QT
Ferrimonas_sp.SCSIO_43195_J8Z22_RS11605                 654  SGKIEIYSDRLATLGSKWELKEG........DVISALP.KYVPTWDGYESLDTPEER
Pseudomonas_sp.SCT_BHD08_RS22425(idrA)                  675  DNDWGEIEGSTVHTKALVAFNTHSG......KAILLKSPWQYAGWIQFYE......AI
Denitromonas_sp.IR12_I8J34_RS03780(idrA)                683  ANDWDEVEGQEVKRKWLYAFGTHSG......KAILLKTPWDYPGWSQFYK......AA
```

```
S_oneidensis_MR-1_dmsA                                 703  DTKDD......YPLQLMGYHTKGRTHSSYHN..VPWLREAAE.DAVWMNPADALGRGL
S_oneidensis_MR-1_SO_4358                              733  DNEFAA....EYPFQLISWHHKGRVHSTHAN..YPWLKEAND.QVAWINPLDAQRYGV
S_sp._LZH-2_JM642_14190                                703  DTKDD......YPLQLMGYHTKGRTHSSYHN..VPWLREAAE.DAVWMNPTDALGRGL
S_xiamenensis_NUITM-VS1_NUITMVS1_29540                 703  DTKDD......YPLQLMGYHTKGRTHSSYHN..VPWLREAAE.DAVWINPTDALGRGL
S_putrefaciens_strain_FDAARGOS_681_FOB89_20615         703  DTKDD......YPLQLMGYHTKGRTHSSYHN..VPWLREAAE.DAVWMNPTDAYGRGL
S_sp._MR-4_Shewmr4_3676                                704  DTIDT......YPLQLMGYHTKGRTHSSYHN..VAWLREAVE.DAVWMNPIDANARGL
S_fidelis_ATCC-BAA-318__L884__L884_RS0114160           708  DAKQD......YPIQLAGYHTKGRTHSSYHN..VPWLREAVE.DAVWVNPMDGAKHGL
S_piezotolerans_WP3_SWP_RS15450                        710  DTSTD......YPIQLAGYHTKGRTHSSYHN..VPWLREAVE.DAVWVNPMDGAKHGL
S_schlegeliana_strain_JCM_JMA39_11561_RS09490          708  DAKAD......YPIQLAGYHTKGRTHSSYHN..VPWLREAVE.DAVWVNPMDGAKHGL
S_marisflavi_strain_EP1_CFF01_RS01175                  705  ETLED......YPLQLIGYHTKGRTHSSYHN..VPWLREAVE.DAVWMNPQDAHARGL
S_sp.SUN_WT4_FJQ87_RS02760                             704  ETSVA......YPLQLASYHTKGRTHSSYHN..VPWLREAVE.DALWINQFDANAYGL
S_sp.MBTL60-112-B2_K5Q73_RS09520                       704  GTKED......YPIQLAGYHTKGRTHSSYHN..VPWLREAVE.DAVWVNPMDGAKHGL
S_sp.MBTL60_112_B1_K5Q83_RS07010                       704  GTKED......YPIQLAGYHTKGRTHSSYHN..VPWLREAVE.DAVWVNPMDGAKHGL
S_enrypsychrophilus_strain_YLB-08_FM038_RS01260        706  DTNID......YPIQLAGYHTKGRTHSSYHN..VPWLREAVE.DAVWVNPMDAAENGL
S_enrypsychrophilus_strain_YLB-08_FM038_RS01535        753  DAIDEG.....FEFQLVNYHTKGRAHSTFHN..CPTLRKVYE.DAIWINPYDANNLGI
S_enrypsychrophilus_strain_YLB-08_FM038_RS23490        712  DESFKD....DFPYQCINYHGKHSAHSIHAS..TPWINEVVE.HHLWVNPNDATKIGV
S_enrypsychrophilus_strain_YLB-08_FM038_RS23525        690  DATAS.....EYPFQCINYHGKHSAHSIHAS..TPWLNEVLE.HHLWLNPVDAGAIGV
S_sp.YLB_09_FS418_RS01245                              706  DTNID......YPIQLAGYHTKGRTHSSYHN..VPWLREAVE.DAVWVNPMDAAENGL
S_sp.YLB_09_FS418_RS01520                              753  DAIDEG.....FEFQLVNYHTKGRAHSTFHN..CPTLRKVYE.DAIWINPYDANNLGI
S_sp.YLB_09_FS418_RS02865                              706  DTNID......YPIQLAGYHTKGRTHSSYHN..VPWLREAVE.DAVWVNPMDAAENGL
S_sp.YLB_09_FS418_RS03140                              753  DAIDEG.....FEFQLVNYHTKGRAHSTFHN..CPTLRKVYE.DAIWINPYDANNLGI
S_sp.YLB_09_FS418_RS25020                              712  DESFKD....DFPYQCINYHGKHSAHSIHAS..TPWINEVVE.HHLWVNPNDATKIGV
S_sp.YLB_09_FS418_RS25055                              690  DATAS.....EYPFQCINYHGKHSAHSIHAS..TPWLNEVLE.HHLWLNPVDAGAIGV
S_sp._WPAGA9_IGB07_RS19235                             702  DTKED......YPLQLAGYHTKGRTHSSYHN..VPWLREAVE.DAVWMNAIDAQQYGV
S_sp._ARC9_LZ_GUY17_RS19100                            713  DTIDAG.....YPLQLCGYHTKGRTHSSYHN..VPWLREAVE.DAVWMNPKDASSRGL
S_psychromarinicola_strain_M2_EGC80_RS00660            684  ETKKK......YPLQLTGYHTKGRAHSSFHN..VPWLREAVQ.DAVWMNPLDANKRGL
S_psychromarinicola_strain_M2_EGC80_RS06745            713  QAIDAG.....YPLQLCGYHTKGRTHSSYHN..VPWLREAVE.DAVWMNPKDASARGL
S_psychromarinicola_strain_M2_EGC80_RS11440            695  .DLVSN.....YPLQLVNFHAKSNVHSGYTN..SQWLKDAMR.RAAWVNPSDASG..F
S_sp.Actino-trap-3_CXF80_RS10115                       684  ETKKK......YPLQLTGYHTKGRAHSSFHN..VPWLREAVQ.DAVWMNPLDASKRGL
S_sp.Actino-trap-3_CXF80_RS15635                       713  QAIDAG.....YPLQLCGYHTKGRTHSSYHN..VPWLREAVE.DAVWMNPKDASARGL
S_livingstonensis_strain_LMG_19866_EGC82_RS20515       713  ETIDAG.....YPLQLCGYHTKGRTHSSYHN..VPWLREAVE.DAVWMNPKDASSRGL
Ferrimonas_lipolytica_strain_S7_HER31_RS14690          667  ETKEKG.....YPIQLIGHHTKGRVHSSFHS..NDWMREAVE.DAVWMNPKDAQERGI
Ferrimonas_lipolytica_strain_S7_HER31_RS15965          745  DTPQGKEEVENYPLQVCGYHTKGRAHSSYHN..VKWLRDAVE.DCVWVNPADAGEFNS
Ferrimonas_lipolytica_strain_S7_HER31_RS04980          701  SETLDE.....FPLQLCGYHTKAQVHTQYQ...STWVKEAMN.PAAWVNPIDAAARSI
S_sp.ISTPL2_CCLCJOKE_1_HUB64_RS13995                   703  DTKDD......YPLQLMGYHTKGRTHSSYHN..VPWLREAAE.DAVWMNPADAYSRGL
S_japonica_strain_KCTC_22435_SJ2017_RS00815            702  DTKED......YPLQLAGYHTKGRTHSSYHN..VPWLREAVE.DAVWMNAIDAQQYGV
S_sp.8A_M2897_RS06500                                  703  DTKDD......YPLQLMGYHTKGRTHSSYHN..VPWLREAAE.DAVWMNPTDALGRGL
S_woodyi_ATCC_51908_Swoo_0233                          706  DTSTD......FPFQLAGYHTKGRTHSSYHN..VPWLREAVE.DAVWVNPMDASEQGL
Ferrimonas_balerica_DSM_9799_Fbal_2476                 685  .TDDPD.....YPFQVAVYHTKGRAHSSFHS..HKVLREAIQ.DALWINPGDAAALGI
Ferrimonas_balerica_DSM_9799_Fbal_3621                 691  ETR......DEYPLQVVGYHTKGRTHSSYHN..VEWLREAVE.DAAWFNPADAAKFGI
S_frigidimarina_NCIMB_400_Sfri_3128                    684  ETKKK......YPLQLTGYHTKGRAHSSFHN..VPWLREAVQ.DAVWMNPIDANQRGL
S_frigidimarina_NCIMB_400_Sfri_3684                    704  ETSED......YPFQLVGYHTKGRTHSSYHN..VPWLREAVE.DAVWMNPADAYKQGL
S_sediminis_HAW-EB3_Ssed_0229                          706  DTSTD......YPIQLAGYHTKGRTHSSYHN..VPWLREAVE.DAVWVNPMDAAENGL
S_sediminis_HAW-EB3_Ssed_0350                          684  ETKKK......YPLQLTGYHTKGRAHSSYHN..VPWLREVVQ.DAVWMNPLDANKRGL
S_sediminis_HAW-EB3_Ssed_0359                          690  DATAS.....EFPLQCINYHGKHSAHSIHAS..TPWLNEVLE.HHLWLNPIDAGAAGV
S_sediminis_HAW-EB3_Ssed_1307                          701  SPELKD.....KPLQLIGHHTKARTHSTFGS..VKWLKEAVNGDAVWINPDDAIKYGI
S_sediminis_HAW-EB3_Ssed_1404                          665  ETK.KQ.....YPLQLIGHHAKGRTHSSFHS..NPWMREAVE.DAVWMNPKDAAARGI
S_sediminis_HAW-EB3_Ssed_2923                          685  ETREK......YPLQLTCYHTKGRVHSSYHN..IPWLREVVQ.DAVWMNPIDAQARNL
S_sp.KX20019_JK628_RS15600                             710  DTSTD......YPIQLAGYHTKGRTHSSYHN..VPWLREAVE.DAVWVNPMDAAKHSL
S_sp.KX20019_JK628_RS20550                             684  ETKKK......YPLQLTGYHTKGRAHSSFHN..VPWLREAVQ.DAVWMNPLDANKRGL
Ferrimonas_sp.SCSIO_43195_J8Z22_RS00580                747  ...DAG.....YPLQLVNYHTKGRAHSTFHN..CPTLRKVYE.DALWINPVDASQQGV
Ferrimonas_sp.SCSIO_43195_J8Z22_RS11605                702  EKAAK......YPLQMHGYHTKGRAHSSYHN..VPWLREAVE.DAVWMNPIDAQERGL
Pseudomonas_sp.SCT_BHD08_RS22425(idrA)                 721  KPRAAK.....GEVWVTNGRVNETWQSGFDDRRKPYLSQRWPEGFIFINPEDARKKGI
Denitromonas_sp.IR12_I8J34_RS03780(idrA)               729  LPRKEK.....GEVWVTNGRVNETWQSGFDDLRKPYLSQRWPYPMLIMHPDEAKPRGI
```

```
                                                                          .                       .           .          .
S_oneidensis_MR-1_dmsA                                   752  RGGDKVQMWNDR.........GTVELPVRITPRVAPGVVALGQGAWYQPD.IARIGRS
S_oneidensis_MR-1_SO_4358                                784  VNGKKMTLTNPRD........AKMVITAKVTTRVMPGVIAMGHGAWHQP........D
S_sp._LZH-2_JM642_14190                                  752  RSGDKVQMWNDR.........GTVELPVRITPRVAPGVVALGQGAWYQPD.IARIGRS
S_xiamenensis_NUITM-VS1_NUITMVS1_29540                   752  RSGDKVQMWNDR.........GTVELPVRITPRVAPGVVALGQGAWYQPD.IARIGRS
S_putrefaciens_strain_FDAARGOS_681_FOB89_20615           752  RNGDKVQMWNDR.........GTVELPIRITPRVAPGVVALGQGAWYQPD.IARIGRS
S_sp._MR-4_Shewmr4_3676                                  753  SQGDTVHVWSQR.........GTIEIPVRITPRVAPGVAALGQGAWYTPDPSGRIGPS
S_fidelis_ATCC-BAA-318__L884__L884_RS0114160             757  SSGDKIEMYNER.........GSVEVTVRITPRVAPGVFALGQGAWYNPGNK..RGST
S_piezotolerans_WP3_SWP_RS15450                          759  SSGDNIEMYNER.........GSIAVKVRITPRVAPGVFALGQGAWFNPGKT..VGST
S_schlegeliana_strain_JCM_JMA39_11561_RS09490            757  SSGDKIEMYNER.........GSVEVTVRITPRVAPGVFALGQGAWYNPGNK..RGST
S_marisflavi_strain_EP1_CFF01_RS01175                    754  EQGVTVHVWSQR.........GTIELPVRITPRIAPGVAALGQGAWYQPDPSGRVGPS
S_sp.SUN_WT4_FJQ87_RS02760                               753  KSGDKVEVYNAR.........GSIEVPVKITPRVAPNVVALGQGAWYLADAKGRVGAS
S_sp.MBTL60-112-B2_K5Q73_RS09520                         753  SSGDKVEMYNER.........GSIEVTVRITPRVAPGVFALGQGAWYNPGNK..RGST
S_sp.MBTL60_112_B1_K5Q83_RS07010                         753  SSGDKVEMYNER.........GSIEVTVRITPRVAPGVFALGQGAWYNPGNK..RGST
S_enrypsychrophilus_strain_YLB-08_FM038_RS01260          755  SSGDKIEMYNER.........GSIAVKVRITPRVAPGVFALGQGAWFSPGNT..VGST
S_enrypsychrophilus_strain_YLB-08_FM038_RS01535          803  ISGEMVDVASPR.........GTIEVPAKVTTRIAMGVVAIGQGAWHKKL........
S_enrypsychrophilus_strain_YLB-08_FM038_RS23490          763  INGQKARVTSKR.........GAIEITVRVTPRIVPGVVALPQGAWRKK........R
S_enrypsychrophilus_strain_YLB-08_FM038_RS23525          740  INGQKVIVESRR.........GKLEITVRVTPRIVPGVVAMPQGQWRRM........K
S_sp.YLB_09_FS418_RS01245                                755  SSGDKIEMYNER.........GSIAVKVRITPRVAPGVFALGQGAWFSPGNT..VGST
S_sp.YLB_09_FS418_RS01520                                803  ISGEMVDVASPR.........GTIEVPAKVTTRIAMGVVAIGQGAWHKKL........
S_sp.YLB_09_FS418_RS02865                                755  SSGDKIEMYNER.........GSIAVKVRITPRVAPGVFALGQGAWFSPGNT..VGST
S_sp.YLB_09_FS418_RS03140                                803  ISGEMVDVASPR.........GTIEVPAKVTTRIAMGVVAIGQGAWHKKL........
S_sp.YLB_09_FS418_RS25020                                763  INGQKARVTSKR.........GAIEITVRVTPRIVPGVVALPQGAWRKK........R
S_sp.YLB_09_FS418_RS25055                                740  INGQKVIVESRR.........GKLEITVRVTPRIVPGVVAMPQGQWRRM........K
S_sp._WPAGA9_IGB07_RS19235                               751  ASGDKVEMYNDR.........GSIEVVVRVTPRVVPGVVALGQGAWFLPDPNGRTGST
S_sp._ARC9_LZ_GUY17_RS19100                              763  ADGDMVHVYNGR.........GTIELPVKLTPRVTPGVCSLGQGAWYKPDASGKVGPS
S_psychromarinicola_strain_M2_EGC80_RS00660              733  STGDKVHIFNDR.........GTIEVEVKVTPRIMVGVTALGQGAWFL........P
S_psychromarinicola_strain_M2_EGC80_RS06745              763  SDGDMVHVYNGR.........GTIEMPIKLTPRVTPGVCALGQGAWYKPDASGKVGPS
S_psychromarinicola_strain_M2_EGC80_RS11440              742  KDGDQIVIESSF.........GKIQVEARVTQRVMPGVISYGQGFKFTPV........
S_sp.Actino-trap-3_CXF80_RS10115                         733  KTGDKVHIFNDR.........GTIEVEVKVTPRIMVGVTALGQGAWFL........P
S_sp.Actino-trap-3_CXF80_RS15635                         763  SDGDMVHVYNGR.........GTIEMPIKLTPRVTPGVCALGQGAWYKPDASGKVGPS
S_livingstonensis_strain_LMG_19866_EGC82_RS20515         763  ADGDMVHVYNGR.........GTIELPVKLTPRVTPGVCSLGQGAWYKPDASGKVGPS
Ferrimonas_lipolytica_strain_S7_HER31_RS14690            717  SQGDTVLIESLR.........GTVKVRARVTPRIMPGVASLPQGAWLDMK........
Ferrimonas_lipolytica_strain_S7_HER31_RS15965            800  ..GDEVIMESPR.........GKLQIRMRITPRVAPGVVALPEGAWYKASAAG.....
Ferrimonas_lipolytica_strain_S7_HER31_RS04980            750  VSGDMIEIESPR.........GKTQVEAKVTPRVMQGVVALGQGLKFNPE........
S_sp.ISTPL2_CCLCJOKE_1_HUB64_RS13995                     752  RNGDKVQMWNDR.........GTVELPIRVTPRVAPGVVALGQGAWYQPD.IARIGRS
S_japonica_strain_KCTC_22435_SJ2017_RS00815              751  ASGDKVEMYNDR.........GSIEVVVRVTPRVVPGVVALGQGAWFLPDPNGRTGST
S_sp.8A_M2897_RS06500                                    752  RSGDKVQMWNDR.........GTVELPVRITPRVAPGVVALGQGAWYQPD.IARIGRS
S_woodyi_ATCC_51908_Swoo_0233                            755  NSGDEIEMYNER.........GSIAVKVRITPRVAPGVLALGQGAWFKPGNT..LGST
Ferrimonas_balerica_DSM_9799_Fbal_2476                   734  QNNDTVRVTSRR.........GVIVVPARVTPRVMPGVAGLGQGAWRNAR.......D
Ferrimonas_balerica_DSM_9799_Fbal_3621                   740  SKGQMITLTSPQ.........GAIRVRAKITPRVMMGVVAMAQGAWYQGTRAG.....
S_frigidimarina_NCIMB_400_Sfri_3128                      733  STGDKVHIFNDR.........GTIEVEVKVTPRIMVGVTALGQGAWFL........P
S_frigidimarina_NCIMB_400_Sfri_3684                      753  TQGSKALIWSQR.........GTIELPVRVTPRVAPGVLALGQGAWYTPDPSGRVGPS
S_sediminis_HAW-EB3_Ssed_0229                            755  SAGDKIEMYNER.........GSIAVKVRITPRVAPGVFALGQGAWFKPGNT..VGST
S_sediminis_HAW-EB3_Ssed_0350                            733  KTGDKVHIFNDR.........GTIEVEVKVTPRIMVGVTALGQGAWFQ........P
S_sediminis_HAW-EB3_Ssed_0359                            740  INGQKVIVESRR.........GKLEITARVTPRIVPGVVAMPQGQWRRM........K
S_sediminis_HAW-EB3_Ssed_1307                            752  VDDQYAIIESLR.........GKVKVKARVTNRIMPGVMSLSQGAWFDPK........
S_sediminis_HAW-EB3_Ssed_1404                            714  SQGDEVLVTSLR.........GKLKVRARVTPRIMPGVTSLPQGAWTKKQ........
S_sediminis_HAW-EB3_Ssed_2923                            734  KTGDLVHVFNDR.........GTLEVEVKVTPRIMIGVTALGQGAWSK.........F
S_sp.KX20019_JK628_RS15600                               759  SSGDNVEMYNER.........GSIAVKVRITPRVTPGVFALGQGAWFSPGKT..VGST
S_sp.KX20019_JK628_RS20550                               733  KTGDKVHIFNDR.........GTIEVEVKVTPRIMVGVTALGQGAWFQ........P
Ferrimonas_sp.SCSIO_43195_J8Z22_RS00580                  794  RDGDRIDVSSRR.........GTIQVTAKLTPRIAMGVVAIGQGAWHQMV........
Ferrimonas_sp.SCSIO_43195_J8Z22_RS11605                  751  SSGDTVQVFNDR.........GTIELPVKVTPRIMPGVTALGQGAWFKG........A
Pseudomonas_sp.SCT_BHD08_RS22425(idrA)                   774  ESGDYVEVVNDTVYIQTGQPQGVLDADLTFNQLMADGHIKITTGRFKTIAVVS.....
Denitromonas_sp.IR12_I8J34_RS03780(idrA)                 782  ESGDFVQVYNDTVYIQMGEPQGVKEDDLYFDTLMKNGHIKTTDGQFVAVAIVS.....
```

```
                                                       .         .                   .         .
S_oneidensis_MR-1_dmsA                           800 GHPIDVGGCINTLTRY.........QPSPFAKGNPQHTNRVQIVKA............
S_oneidensis_MR-1_SO_4358                        826 ANGVDHGGSVNVLVSR.........KASPTARGIPANTARIRIVQA............
S_sp._LZH-2_JM642_14190                          800 GHPIDVGGCINTLTRY.........QPSPFAKGNPQHTNRVQIVKA............
S_xiamenensis_NUITM-VS1_NUITMVS1_29540           800 GHPIDVGGCINTLTRY.........QPSPFAKGNPQHTNRVQIVKA............
S_putrefaciens_strain_FDAARGOS_681_FOB89_20615   800 GHPIDVGGCINTLTRY.........QPSPFAKGNPQHTNRVQIVKA............
S_sp._MR-4_Shewmr4_3676                          802 GHVIDIGGCINTLTRY.........QPSPAAKGNPQHTNRVQIAKA............
S_fidelis_ATCC-BAA-318__L884__L884_RS0114160     804 GHIIDEGGAINSLTRY.........QPSPVAKGNPQHTIRVAIKKI............
S_piezotolerans_WP3_SWP_RS15450                  806 GHIIDEGGAINSLTRY.........QPSPVAKGNPQHTIRVAIKKI............
S_schlegeliana_strain_JCM_JMA39_11561_RS09490    804 GHIVDEGGAINSLTRY.........QPSPVAKGNPQHTIRVAIKKI............
S_marisflavi_strain_EP1_CFF01_RS01175            803 GHVIDIGGCINTLTRY.........QPSPSAKGNPQHTNRVQIARA............
S_sp.SUN_WT4_FJQ87_RS02760                       802 GKVIDVGGAVNSLTRY.........QPSPVAKGNPQLTIRVQIKKA............
S_sp.MBTL60-112-B2_K5Q73_RS09520                 800 GHIVDEGGAINSLTRY.........QPSPVAKGNPQHTIRVAIKKI............
S_sp.MBTL60_112_B1_K5Q83_RS07010                 800 GHIVDEGGAINSLTRY.........QPSPVAKGNPQHTIRVAIKKI............
S_enrypsychrophilus_strain_YLB-08_FM038_RS01260  802 GHIIDEGGAINTLTRY.........QPSPVAKGNPQHTNRVAIKKI............
S_enrypsychrophilus_strain_YLB-08_FM038_RS01535  844 .GNKDVGGCANTLTRQ.........KPTPLAKGNPMNSVRVRITKA............
S_enrypsychrophilus_strain_YLB-08_FM038_RS23490  804 SDGVDTGGAVNTLTSS.........KPSPVAKSIRSNTNRVRIQPA............
S_enrypsychrophilus_strain_YLB-08_FM038_RS23525  781 GD.IDVGGCANTLTDS.........KPSPVAKSFRSNTNRVRVYSA............
S_sp.YLB_09_FS418_RS01245                        802 GHIIDEGGAINTLTRY.........QPSPVAKGNPQHTNRVAIKKI............
S_sp.YLB_09_FS418_RS01520                        844 .GNKDVGGCANTLTRQ.........KPTPLAKGNPMNSVRVRITKA............
S_sp.YLB_09_FS418_RS02865                        802 GHIIDEGGAINTLTRY.........QPSPVAKGNPQHTNRVAIKKI............
S_sp.YLB_09_FS418_RS03140                        844 .GNKDVGGCANTLTRQ.........KPTPLAKGNPMNSVRVRITKA............
S_sp.YLB_09_FS418_RS25020                        804 SDGVDTGGAVNTLTSS.........KPSPVAKSIRSNTNRVRIQPA............
S_sp.YLB_09_FS418_RS25055                        781 GD.IDVGGCANTLTDS.........KPSPVAKSFRSNTNRVRVYSA............
S_sp._WPAGA9_IGB07_RS19235                       800 GKVVDIGGAINSLTKY.........MPSPVAKGNPQHTNRVQIRKVL...........
S_sp._ARC9_LZ_GUY17_RS19100                      812 GHVVDIGGCINTLTKY.........HPSPVSKGNPQHTNRVQVVKA............
S_psychromarinicola_strain_M2_EGC80_RS00660      773 GGAVDKGGCLNVLTTQ.........RTTPITKGNPQHTNLVEIRKV............
S_psychromarinicola_strain_M2_EGC80_RS06745      812 GHVVDIGGCINTLTKY.........HPSPISKGNPQHTNRVQVVKA............
S_psychromarinicola_strain_M2_EGC80_RS11440      783 .GDLDIGGNFNSLTSHT........MTSTVAKGTGVNSNRVRIYKA............
S_sp.Actino-trap-3_CXF80_RS10115                 773 GGAVDKGGCLNVLTTQ.........RTTPVTKGNPQHTNLVEIRKV............
S_sp.Actino-trap-3_CXF80_RS15635                 812 GHVVDIGGCINTLTKY.........HPSPISKGNPQHTNRVQVVKA............
S_livingstonensis_strain_LMG_19866_EGC82_RS20515 812 GHVVDIGGCINTLTKY.........HPSPVSKGNPQHTNRVQVVKV............
Ferrimonas_lipolytica_strain_S7_HER31_RS14690    758 .DGIDEGGCVNTLTSL.........RPTAIGKCNPQHTNLVEISLS............
Ferrimonas_lipolytica_strain_S7_HER31_RS15965    842 ..EVDSGGCVNTLTKY.........HPCPVSRGNTQHTIRVKLYKANA..........
Ferrimonas_lipolytica_strain_S7_HER31_RS04980    791 .GGVCKGGSSNTLTSHD........YSSPVDKACGVATCRINIRKL............
S_sp.ISTPL2_CCLCJOKE_1_HUB64_RS13995             800 GHPIDVGGCINTLTRY.........QPSPFAKGNPQHTNRVQIVKA............
S_japonica_strain_KCTC_22435_SJ2017_RS00815      800 GKVVDIGGAINSLTKY.........MPSPVAKGNPQHTNRVQIRKVL...........
S_sp.8A_M2897_RS06500                            800 GHSIDVGGCINTLTRY.........QPSPFAKGNPQHTNRVQIVKA............
S_woodyi_ATCC_51908_Swoo_0233                    802 GHIIDEGGAINSLTRY.........QPSPVAKGNPQHTNRVAIKKI............
Ferrimonas_balerica_DSM_9799_Fbal_2476           776 AGQEDMGGCINTLTDH.........RPSPLAKGCTGGTLRVKIERA............
Ferrimonas_balerica_DSM_9799_Fbal_3621           784 ..EVDQGGCINALTKY.........HPTPVSKGNPQHTIRVKVTA.............
S_frigidimarina_NCIMB_400_Sfri_3128              773 GGNVDKGGCLNVLTTQ.........RTTPITKGNPQHTNLVEIRKV............
S_frigidimarina_NCIMB_400_Sfri_3684              802 GHIIDIGGCINTLTRY.........QPSPLAKGNPQHTNRVQIASA............
S_sediminis_HAW-EB3_Ssed_0229                    802 GHIIDEGGAINTLTRY.........QPSPVAKGNPQHTNRVAIKKI............
S_sediminis_HAW-EB3_Ssed_0350                    773 GGDVDKGGCLNVLTTQ.........RTTPVTKGNPQHTNLVEIRKV............
S_sediminis_HAW-EB3_Ssed_0359                    781 GD.IDVGGCANTLTDS.........KPSPVAKSFRSNTNRVRVYSA............
S_sediminis_HAW-EB3_Ssed_1307                    793 .NDVDVGGCVNALTSQG........RPSPLAKGNPQHTNRVRIYKA............
S_sediminis_HAW-EB3_Ssed_1404                    755 .GDVDVGGCVNALTSQ.........RPTAIGKCNPQHTNLVEISLY............
S_sediminis_HAW-EB3_Ssed_2923                    774 EDGIDKGGNVNTITSQ.........RPTPLSKGNPQHTNRVEIRRA............
S_sp.KX20019_JK628_RS15600                       806 GHIIDEGGAINSLTRY.........QPSPVAKGNPQHTIRVAIKKI............
S_sp.KX20019_JK628_RS20550                       773 GGDVDKGGCLNVLTTQ.........RTTPVTKGNPQHTNLVEIRKV............
Ferrimonas_sp.SCSIO_43195_J8Z22_RS00580          835 .AGKDVGGCANTLTRQ.........QCSPLAKGNPMNTVKVNISKA............
Ferrimonas_sp.SCSIO_43195_J8Z22_RS11605          792 GKTIDKGGCLNTLTSQ.........RPTPISKGNGQHTIRVQINKA............
Pseudomonas_sp.SCT_BHD08_RS22425(idrA)           827 .DEMRPGVCQANFNVPSSPANAVVSAVPDPMTNNYRYKLGRGVLNKVGESPYKHNFTQ
Denitromonas_sp.IR12_I8J34_RS03780(idrA)         835 .EEMRPGVV.................................................
```

**S_oneidensis_MR-1_dmsA**		....
**S_oneidensis_MR-1_SO_4358**		....
**S_sp._LZH-2_JM642_14190**		....
**S_xiamenensis_NUITM-VS1_NUITMVS1_29540**		....
**S_putrefaciens_strain_FDAARGOS_681_FOB89_20615**		....
**S_sp._MR-4_Shewmr4_3676**		....
**S_fidelis_ATCC-BAA-318__L884__L884_RS0114160**		....
**S_piezotolerans_WP3_SWP_RS15450**		....
**S_schlegeliana_strain_JCM_JMA39_11561_RS09490**		....
**S_marisflavi_strain_EP1_CFF01_RS01175**		....
**S_sp.SUN_WT4_FJQ87_RS02760**		....
**S_sp.MBTL60-112-B2_K5Q73_RS09520**		....
**S_sp.MBTL60_112_B1_K5Q83_RS07010**		....
**S_enrypsychrophilus_strain_YLB-08_FM038_RS01260**		....
**S_enrypsychrophilus_strain_YLB-08_FM038_RS01535**		....
**S_enrypsychrophilus_strain_YLB-08_FM038_RS23490**		....
**S_enrypsychrophilus_strain_YLB-08_FM038_RS23525**		....
**S_sp.YLB_09_FS418_RS01245**		....
**S_sp.YLB_09_FS418_RS01520**		....
**S_sp.YLB_09_FS418_RS02865**		....
**S_sp.YLB_09_FS418_RS03140**		....
**S_sp.YLB_09_FS418_RS25020**		....
**S_sp.YLB_09_FS418_RS25055**		....
**S_sp._WPAGA9_IGB07_RS19235**		....
**S_sp._ARC9_LZ_GUY17_RS19100**		....
**S_psychromarinicola_strain_M2_EGC80_RS00660**		....
**S_psychromarinicola_strain_M2_EGC80_RS06745**		....
**S_psychromarinicola_strain_M2_EGC80_RS11440**		....
**S_sp.Actino-trap-3_CXF80_RS10115**		....
**S_sp.Actino-trap-3_CXF80_RS15635**		....
**S_livingstonensis_strain_LMG_19866_EGC82_RS20515**		....
**Ferrimonas_lipolytica_strain_S7_HER31_RS14690**		....
**Ferrimonas_lipolytica_strain_S7_HER31_RS15965**		....
**Ferrimonas_lipolytica_strain_S7_HER31_RS04980**		....
**S_sp.ISTPL2_CCLCJOKE_1_HUB64_RS13995**		....
**S_japonica_strain_KCTC_22435_SJ2017_RS00815**		....
**S_sp.8A_M2897_RS06500**		....
**S_woodyi_ATCC_51908_Swoo_0233**		....
**Ferrimonas_balerica_DSM_9799_Fbal_2476**		....
**Ferrimonas_balerica_DSM_9799_Fbal_3621**		....
**S_frigidimarina_NCIMB_400_Sfri_3128**		....
**S_frigidimarina_NCIMB_400_Sfri_3684**		....
**S_sediminis_HAW-EB3_Ssed_0229**		....
**S_sediminis_HAW-EB3_Ssed_0350**		....
**S_sediminis_HAW-EB3_Ssed_0359**		....
**S_sediminis_HAW-EB3_Ssed_1307**		....
**S_sediminis_HAW-EB3_Ssed_1404**		....
**S_sediminis_HAW-EB3_Ssed_2923**		....
**S_sp.KX20019_JK628_RS15600**		....
**S_sp.KX20019_JK628_RS20550**		....
**Ferrimonas_sp.SCSIO_43195_J8Z22_RS00580**		....
**Ferrimonas_sp.SCSIO_43195_J8Z22_RS11605**		....
**Pseudomonas_sp.SCT_BHD08_RS22425(idrA)**	**884**	MSLKPRNIV
**Denitromonas_sp.IR12_I8J34_RS03780(idrA)**		....
