# Supplementary material for: Global occurrence of the bacteria with capability for extracellular reduction of iodate
Source: Front Microbiol. 2022 Nov 25;13:1070601. doi: 10.3389/fmicb.2022.1070601 (PMC9732548; doi:10.3389/fmicb.2022.1070601)
Supplement: Supplementary file 8 [file Data_Sheet_2.PDF]

```
S_oneidensis_MR-1_dmsB                                   1  ...MTQQT..QYGFYFDSSKCTGCKTCHIACKDRMTGLVRAKDDVMNNGVA...NLPGI
S_oneidensis_MR-1_SO_4357                                1  ...MQEPT..QYGFYVDTTKCSGCKTCQVACKDRSD..................IEVGK
S_sp._LZH-2__JM642_14185                                 1  ...MTQQT..QYGFYFDSSKCTGCKTCHIACKDRMTGLVRAKDDVMNNGVA...NLPGI
S_xiamenensis_NUITM-VS1_NUITMVS1_29530                   1  ...MTQQT..QYGFYFDSSKCTGCKTCHIACKDRMTGLVRAKDDVMNNGVA...NLPGI
S_putrefaciens_strain_FDAARGOS_681_FOB89_20610           1  ...MTQQT..QYGFYFDSSKCTGCKTCHIACKDRMTGLVRAKDDVLNNGVA...NLPGI
S_sp._MR-4__Shewmr4_3678                                 1  ...MTQET..QYGFYFDSSKCTGCKACHIACKDRMTGLIRANDG..NGSVA...NLPGV
S_fidelis_ATCC-BAA-318__L884__L884_RS0114165             1  ...MTEAT..QYGFYVDSTKCTGCKACHVSCKDRQGNEIRNNTNPEQNGVP...ALNGV
S_piezotolerans_WP3_SWP_RS15445                          1  ...MTEPT..QYGFYVDSTKCTGCKACHVSCKDRQSNEIRNNSNPQDNTLP...ALNGV
S_schlegeliana_strain_JCM_JMA39_11561_RS09485            1  ...MTEPT..QYGFYVDSTKCTGCKACHVSCKDRQGNEIRNNTNPEANGVP...ALNGV
S_marisflavi_strain_EP1_CFF01_RS01170                    1  ...MTEQT..QYGFYLDTTKCTGCKACHVSCKDRQGDIIR.TTTSPEQGIP...ALTGV
S_japonica_strain_KCTC_22435_SJ2017_RS00810              1  ...MSDAI..QYSFYVDSTKCTGCKACHISCKDRQGEQIRNATKPVENGVP...SLNGV
S_sp.SUN_WT4_FJQ87_RS02765                               1  ...MTQPT..QYGFYVDTTKCTGCKACHVSCKDRQADTIRATHNPMNGGVP...SLAGV
S_sp.MBTL60-112-B2_K5Q73_RS09515                         1  ...MTEAT..QYGFYVDSTKCTGCKACHVSCKDRQGNEIRNNTNPEQNGVP...ALNGV
S_sp.MBTL60_112_B1_K5Q83_RS07015                         1  ...MTEAT..QYGFYVDSTKCTGCKACHVSCKDRQGNEIRNNTNPEQNGVP...ALNGV
S_enrypsychrophilus_strain_YLB-08_FM038_RS01265          1  ...MTQAT..QYGFYVDSTKCSGCKACHVSCKDRQSNEIRNNSNPEGNALP...ALEGV
S_enrypsychrophilus_strain_YLB-08_FM038_RS01530          1  MTKATENQ..AFAFYFDASRCNGCKGCQVSCLDGRG..................LSLDR
S_enrypsychrophilus_strain_YLB-08_FM038_RS23495          1  .....MTQ..QYGFYVDVATCSGCKTCMVSCKDNKD..................LEIGR
S_enrypsychrophilus_strain_YLB-08_FM038_RS23530          1  .......M..QLGFYVDTEICSGCKACMVSCKDKND..................LNVGR
S_sp.YLB_09_FS418_RS01250                                1  ...MTQAT..QYGFYVDSTKCSGCKACHVSCKDRQSNEIRNNSNPEGNALP...ALEGV
S_sp.YLB_09_FS418_RS01515                                1  MTKATENQ..AFAFYFDASRCNGCKGCQVSCLDGRG..................LSLDR
S_sp.YLB_09_FS418_RS02870                                1  ...MTQAT..QYGFYVDSTKCSGCKACHVSCKDRQSNEIRNNSNPEGNALP...ALEGV
S_sp.YLB_09_FS418_RS03135                                1  MTKATENQ..AFAFYFDASRCNGCKGCQVSCLDGRG..................LSLDR
S_sp.YLB_09_FS418_RS25025                                1  .....MTQ..QYGFYVDVATCSGCKTCMVSCKDNKD..................LEIGR
S_sp.YLB_09_FS418_RS25060                                1  .......M..QLGFYVDTEICSGCKACMVSCKDKND..................LNVGR
S_sp._WPAGA9__IGB07_RS19230                              1  ...MSDAI..QYSFYVDSTKCTGCKACHISCKDRQGEQIRNATKPVENGVP...SLNGV
S_sp._ARC9_LZ_GUY17_RS19095                              1  ...MTQET..QLGFYFDSSKCTGCKTCHIACKDRMVGKTRSKDDVTNQGVS...AMPGM
S_psychromarinicola_strain_M2_EGC80_RS00655              1  ...MSNNI..QYGFHVDASKCTGCKTCQISCKDRKD..................LPVGI
S_psychromarinicola_strain_M2_EGC80_RS06750              1  ...MTQET..QLGFYFDSSKCTGCKTCHIACKDRMVGITRNKDDVSTQGVS...ALPGM
S_psychromarinicola_strain_M2_EGC80_RS11435              1  ...MADSF..QLGFHFDSSKCSGCKACDIICKSKFD..................AKPTQ
S_sp.Actino-trap-3__CXF80_RS10110                        1  ...MSNNI..QYGFHVDASKCTGCKTCQISCKDRKD..................LPVGI
S_sp.Actino-trap-3__CXF80_RS15640                        1  ...MTQET..QLGFYFDSSKCTGCKTCHIACKDRMVGITRNKDDVSTQGVS...ALPGM
S_livingstonensis_strain_LMG_19866_EGC82_RS20510         1  ...MTQET..QLGFYFDSSKCTGCKTCHIACKDRMVGKTRSKDDVTNQGVS...AMPGM
Ferrimonas_lipolytica_strain_S7_HER31_RS14685            1  ...MTDST..QYGFFIDTAKCTGCKTCHIACKDRKD..................LPLGI
Ferrimonas_lipolytica_strain_S7_HER31_RS15970            1  ...MTEQV..QFGFFIDTTKCTGCKTCHVACKDDNSRLATGSILPDGSNEPTNLMPVEG
Ferrimonas_lipolytica_strain_S7_HER31_RS04975            1  ...MTDET..QLAFHFDQTKCNGCKACHMACKVKLE..................SNLNE
S_sp.ISTPL2_CCLCJOKE_1_HUB64_RS13990                     1  ...MTQQT..QYGFYFDSSKCTGCKTCHIACKDRMTGLVRVKDDVLNNGVA...NLPGI
S_sp.8A_M2897_RS06505                                    1  ...MTQQT..QYGFYFDSSKCTGCKTCHIACKDRMTGLVRAKDDVMNNGVA...NLPGI
S_woodyi_ATCC_51908_Swoo_0234                            1  ...MTQPT..QYGFYVDSTKCTGCKACHVSCKDRQSNEIRNNSNPEGNALP...ALEGV
Ferrimonas_balerica_DSM_9799_Fbal_2475                   1  ...MMEKTTQQYGFYFDASRCSGCKACHVACKDRMN..................APEGV
Ferrimonas_balerica_DSM_9799_Fbal_3622                   1  ...MNEPK..QYGFYVDTTKCTGCKTCHVSCKDRKD..................LPKDV
S_frigidimarina_NCIMB_400_Sfri_3127                      1  ...MSNNI..QYGFHVDASKCTGCKTCQISCKDRKD..................LPVGI
S_frigidimarina_NCIMB_400_Sfri_3683                      1  ...MTEQT..QYGFYLDTTKCTGCKACHISCKDRQGDIIR.KTNPIEQGVP...ALEGI
S_sediminis_HAW-EB3_Ssed_0230                            1  ...MTQPT..QYGFYVDSTKCSGCKACHVSCKDRQSNEIRNNSNPEGNALP...ALEGV
S_sediminis_HAW-EB3_Ssed_0349                            1  ...MSNNI..QYGFHVDASKCTGCKTCQVSCKDRKD..................LPVGI
S_sediminis_HAW-EB3_Ssed_0358                            1  .......M..QLGFYVDTSICSGCKACMVSCKDKND..................LNVGR
S_sediminis_HAW-EB3_Ssed_1308                            1  ...MTNNT..QYGFHYDASKCNGCKGCHIICKSRFD..................GEDGV
S_sediminis_HAW-EB3_Ssed_1405                            1  ...MTDKI..QYGFFVDTTKCTGCKTCQVACKDRSD..................LSLGV
S_sediminis_HAW-EB3_Ssed_2922                            1  ...MSNNV..QYGFYVDSSKCTGCKTCQVTCKDRKD..................LPVGI
S_sp.KX20019_JK628_RS15595                               1  ...MTEAT..QYGFYVDSTQCTGCKACHVSCKDRQSNEIRNNSNPQDNTLP...ALDGV
S_sp.KX20019_JK628_RS20555                               1  ...MSNNI..QYGFHVDASKCTGCKTCQISCKDRKD..................LPVGI
Ferrimonas_sp.SCSIO_43195_J8Z22_RS00575                  1  MAELNQNT..NYAFYFDASRCNGCKGCQIACKDGRS..................LSLDR
Ferrimonas_sp.SCSIO_43195_J8Z22_RS11610                  1  ...MSDNK..QYGFYVDTSKCTGCKTCQISCKDRSD..................LPVGV
Pseudomonas_sp.SCT_BHD08_RS22420(idrB)                   1  ...MSENIIPVRAVPAHDHEHDGERACMS..............................
Denitromonas_sp.IR12_I8J34_RS03785(idrB)                 1  ...MTTHPIHLH...HDDPAHGGERACMS..............................
```

S_oneidensis_MR-1_dmsB	52	IWRRVYEYGGGNWSQN.VDGSFE..QNVFAYYMSIGCNHCSQPVCVKACPTGAMHKRRE
S_oneidensis_MR-1_SO_4357	37	QWRRTYEYCGGNWTAD.GQGAYH..QDVFAYYISISCNHCSNPVCVKACPTGAMYKERS
S_sp._LZH-2__JM642_14185	52	IWRRVYEYGGGNWSQN.IDGSFE..QNVFAYYMSIGCNHCSEPVCVKACPTGAMHKRRE
S_xiamenensis_NUITM-VS1_NUITMVS1_29530	52	IWRRVYEYGGGNWSQN.LDGSFE..QNVFAYYMSIGCNHCSEPVCVKACPTGAMHKRRE
S_putrefaciens_strain_FDAARGOS_681_FOB89_20610	52	IWRRVYEYGGGSWSQN.GDGSFE..QNVFAYYMSIGCNHCNEPVCVKACPTGAMHKRRE
S_sp._MR-4__Shewmr4_3678	50	LWRRVTEYGGGNWSQN.ADGSFE..QNVFAYYTSIGCNHCSEPVCVKACPTGAMHKRKD
S_fidelis_ATCC-BAA-318__L884__L884_RS0114165	52	TWRRVYEYGGGQWTEG.QNGCFE..QSVFAYYMSIGCNHCSEPVCVKAACPTGAMHKRRE
S_piezotolerans_WP3_SWP_RS15445	52	TWRRVYEYGGGEWTVG.NNGCFE..QNVFAYYMSIGCNHCSEPVCVKACPTGAMHKRRE
S_schlegeliana_strain_JCM_JMA39_11561_RS09485	52	TWRRVYEYGGGQWTEG.QNGCFE..QSVFAYYMSIGCNHCSEPVCVKACPTGAMHKRRE
S_marisflavi_strain_EP1_CFF01_RS01170	51	NWRRVYEYGGGSWSTN.ADGSFE..QNVFTYYASISCNHCSEPVCVKACPTGAMHKRRE
S_japonica_strain_KCTC_22435_SJ2017_RS00810	52	NWRRVYEYGGGTWNVDPATGTFE..QDVFAYYMSVGCNHCSEPVCVKACPTGAMHKRRE
S_sp.SUN_WT4_FJQ87_RS02765	52	TWRRVYEYGGGECSMNPATKVFE..NNVFAYYMSIGCNHCSEPVCVKACPTGAMHKRRE
S_sp.MBTL60-112-B2_K5Q73_RS09515	52	TWRRVYEYGGGQWTEG.QNGCFE..QSVFAYYMSIGCNHCSEPVCVKACPTGAMHKRRE
S_sp.MBTL60_112_B1_K5Q83_RS07015	52	TWRRVYEYGGGQWTEG.QNGCFE..QSVFAYYMSIGCNHCSEPVCVKACPTGAMHKRRE
S_enrypsychrophilus_strain_YLB-08_FM038_RS01265	52	TWRRVYEYGGGEWTKG.NNGCFE..QNVFAYYMSIGCNHCSEPVCVKACPTGAMHKRRE
S_enrypsychrophilus_strain_YLB-08_FM038_RS01530	40	NFRRVYEYGGGTFTKN.ADGTMS..QDAFAYYASVSCNHCEKPACTAACPTGAMHKKAN
S_enrypsychrophilus_strain_YLB-08_FM038_RS23495	35	NFRRVYEYAGGSCSEN.ANGVLS..QSVFAYYVSMACNHCDTPACTKACPTGAMYKEPE
S_enrypsychrophilus_strain_YLB-08_FM038_RS23530	33	NFRRVYEYAGGSCTDN.GNGVFS..QSVFAYYTSISCNHCDIPACTKACPTGAMHKRPE
S_sp.YLB_09_FS418_RS01250	52	TWRRVYEYGGGEWTKG.NNGCFE..QNVFAYYMSIGCNHCSEPVCVKACPTGAMHKRRE
S_sp.YLB_09_FS418_RS01515	40	NFRRVYEYGGGTFTKN.ADGTMS..QDAFAYYASVSCNHCEKPACTAACPTGAMHKKAN
S_sp.YLB_09_FS418_RS02870	52	TWRRVYEYGGGEWTKG.NNGCFE..QNVFAYYMSIGCNHCSEPVCVKACPTGAMHKRRE
S_sp.YLB_09_FS418_RS03135	40	NFRRVYEYGGGTFTKN.ADGTMS..QDAFAYYASVSCNHCEKPACTAACPTGAMHKKAN
S_sp.YLB_09_FS418_RS25025	35	NFRRVYEYAGGSCSEN.ANGVLS..QSVFAYYVSMACNHCDTPACTKACPTGAMYKEPE
S_sp.YLB_09_FS418_RS25060	33	NFRRVYEYAGGSCTDN.GNGVFS..QSVFAYYTSISCNHCDIPACTKACPTGAMHKRPE
S_sp._WPAGA9__IGB07_RS19230	52	NWRRVYEYGGGTWNVDPATGTFE..QDVFAYYMSVGCNHCSEPVCVKACPTGAMHKRRE
S_sp._ARC9_LZ_GUY17_RS19095	52	LWRRVYEYGGGSWDQN.GDESYN..QNVFAYYASVGCNHCNEPVCVKACPTGAMHKRKE
S_psychromarinicola_strain_M2_EGC80_RS00655	37	NWRRVYEYGGGQWTEH.GDGTVS..QNVFAYYLSIACNHCSEPVCVKACPTGAMHKRRE
S_psychromarinicola_strain_M2_EGC80_RS06750	52	LWRRVYEYGGGTWEQN.SDESYE..QNIFAYYASIGCNHCNEPVCVKACPTGAMHKRKE
S_psychromarinicola_strain_M2_EGC80_RS11435	37	NTRRVYEYVGGSVTEN.SDNTVS..SSVFAYYVSIGCNHCSEPVCVKACPTGACHKQSS
S_sp.Actino-trap-3__CXF80_RS10110	37	NWRRVYEYGGGQWAEH.GDGTVS..QNVFAYYLSIACNHCSEPVCVKACPTGAMHKRRE
S_sp.Actino-trap-3__CXF80_RS15640	52	LWRRVYEYGGGTWEQN.SDESYE..QNIFAYYASIGCNHCNEPVCVKACPTGAMHKRKE
S_livingstonensis_strain_LMG_19866_EGC82_RS20510	52	LWRRVYEYGGGSWDQN.GDESYN..QNVFAYYASVGCNHCNEPACVKACPTGAMHKRKE
Ferrimonas_lipolytica_strain_S7_HER31_RS14685	37	KWRRVYEFGGGQWTEN.ENGSFD..QDVYSYYVSLGCNHCSNPVCVKACPTGACYKRSQ
Ferrimonas_lipolytica_strain_S7_HER31_RS15970	55	KWRRVYEFGGGTMGTN.GKGAFTGFSGVFSYYASIGCNHCSHPVCVQACPTGACHKRQK
Ferrimonas_lipolytica_strain_S7_HER31_RS04975	37	IPRRVYEYVGGGCVDN.GDNTFT..TTVFGYYTSVGCNHCSEPVCVKACPTGAMYKQAS
S_sp.ISTPL2_CCLCJOKE_1_HUB64_RS13990	52	IWRRVYEYGGGSWSLN.GDGSFE..QNVFAYYMSIGCNHCNEPVCVKACPTGAMHKRRE
S_sp.8A_M2897_RS06505	52	IWRRVYEYGGGNWSQN.IDGSFE..QNVFAYYMSIGCNHCSEPVCVKACPTGAMHKRRE
S_woodyi_ATCC_51908_Swoo_0234	52	TWRRVYEYGGGEWTVG.NNGCFE..QNVFAYYMSIGCNHCSEPVCVKACPTGAMHKRRQ
Ferrimonas_balerica_DSM_9799_Fbal_2475	39	IPRRVYEYSGGGFIRD.EQGAMT..ASVFSYYTSVGCNHCSHPVCVKACPTGACHKSRS
Ferrimonas_balerica_DSM_9799_Fbal_3622	37	KWRRVYEYGGGSWQEN.ADGSVE..QNVFSYYVSIGCNHCSNPVCVKACPTGAMHKRRS
S_frigidimarina_NCIMB_400_Sfri_3127	37	NWRRVYEYGGGQWTEH.GDGTVS..QNVFAYYMSIACNHCSEPVCVKACPTGAMHKRRE
S_frigidimarina_NCIMB_400_Sfri_3683	51	NWRRVYEYGGGDWSTN.ADGSFE..QNVFSYYTSISCNHCNEPVCVKACPTGAMHKRRE
S_sediminis_HAW-EB3_Ssed_0230	52	TWRRVYEYGGGEWTKG.NNGCFE..QNVFAYYMSIGCNHCSEPVCVKACPTGAMHKRRE
S_sediminis_HAW-EB3_Ssed_0349	37	NWRRVYEYGGGQWTEN.GDGTVS..QNVFAYYMSIGCNHCSEPVCVKACPTGAMHKRRE
S_sediminis_HAW-EB3_Ssed_0358	33	NFRRVYEYAGGSCTDN.GNGVFS..QSVFAYYTSISCNHCDTPACTKACPTGAMHKRSE
S_sediminis_HAW-EB3_Ssed_1308	37	IPRRVYEYVGGGYADN.NDGTVT..SNTFAYYTSISCNHCSEPVCVKACPTGAMHKRSK
S_sediminis_HAW-EB3_Ssed_1405	37	KWRRVYEYGGGSWTEN.EDGTFN..NNVFAHYTSISCNHCSHPVCVKACPTGACHKRKS
S_sediminis_HAW-EB3_Ssed_2922	37	NWRRVYEYGGGLWTEN.ADGTVN..QNVFAYYTSIGCNHCSEPACVKACPVGAMHKRKQ
S_sp.KX20019_JK628_RS15595	52	TWRRVYEYGGGDWTVG.NNGCFE..QNVFAYYMSIGCNHCSEPVCVKACPTGAMHKRRE
S_sp.KX20019_JK628_RS20555	37	NWRRVYEYGGGQWTEH.GDGTVS..QNVFAYYMSIGCNHCSEPVCVKACPTGAMHKRRE
Ferrimonas_sp.SCSIO_43195_J8Z22_RS00575	40	HFRRVYEYAGGTFSQS.ADGVMH..QSAFAYYLSVSCNHCDEPACTKACPTGAMHKRTD
Ferrimonas_sp.SCSIO_43195_J8Z22_RS11610	37	NFRRVYEFGGGQWTRK.EDGTAT..QNVFAYYASISCNHCSEPVCTKACPTGAMHKRRS
Pseudomonas_sp.SCT_BHD08_RS22420(idrB)	27	..RRRFLLFGGTSVALLSIASLP..G.VAQVMQALKADYARQRIGSLSALKTGEPLDFN
Denitromonas_sp.IR12_I8J34_RS03785(idrB)	24	..RRSFLLAGG...AMVTLASLP..GTAVAALKALKADYPAVKIGKLSRLKTGEPLEFA

S_oneidensis_MR-1_dmsB	108	DGLVQVATELCIGCESCARACPYDAPQLDIERKVMTKCDGCSDR........LAEGKKP
S_oneidensis_MR-1_SO_4357	93	TGLVKVNQDLCIGCESCARACPYDAPQIDPQRKVMTKCDGCSDR........VAKGLKP
S_sp._LZH-2__JM642_14185	108	DGLVQVATELCIGCESCARACPYDAPQLDIERKVMTKCDGCSDR........LAEGKKP
S_xiamenensis_NUITM-VS1_NUITMVS1_29530	108	DGLVQVATELCIGCESCARACPYDAPQLDIERKVMTKCDGCSDR........LAEGKKP
S_putrefaciens_strain_FDAARGOS_681_FOB89_20610	108	DGLVQVATELCIGCESCARACPYDAPQLDIERKVMTKCDGCSDR........LAEGKKP
S_sp._MR-4__Shewmr4_3678	106	NGLVLVESSICIGCGSCARACPYDAPQLDTQRKVMTKCDGCYDR........LADGKKP
S_fidelis_ATCC-BAA-318__L884__L884_RS0114165	108	DGLVHVAQDLCIGCESCARACPYDAPQIDRERKVMTKCDGCFER........IAEGRKP
S_piezotolerans_WP3_SWP_RS15445	108	DGLVHVAEDICIGCESCSRACPYDAPQIDRERKVMTKCDGCFER........IADGRKP
S_schlegeliana_strain_JCM_JMA39_11561_RS09485	108	DGLVHVAQDLCIGCESCARACPYDAPQIDRERKVMTKCDGCFER........IAEGRKP
S_marisflavi_strain_EP1_CFF01_RS01170	107	DGLVHVATELCIGCSSCAKACPYDAPQLDTERKVMTKCDGCYDR........LAEGKQP
S_japonica_strain_KCTC_22435_SJ2017_RS00810	109	DGLVLVAEELCIACESCSRACPYDAPQLDQERKVMTKCDGCYDR........LAEGRKP
S_sp.SUN_WT4_FJQ87_RS02765	109	DGLVHVAQDLCIGCESCARACPYDAPQIDRERKVMTKCDGCYER........LAEGKKP
S_sp.MBTL60-112-B2_K5Q73_RS09515	108	DGLVHVAQDLCIGCESCARACPYDAPQIDRERKVMTKCDGCFER........IADGRKP
S_sp.MBTL60_112_B1_K5Q83_RS07015	108	DGLVHVAQDLCIGCESCARACPYDAPQIDRERKVMTKCDGCFER........IADGRKP
S_enrypsychrophilus_strain_YLB-08_FM038_RS01265	108	DGLVHIASELCIGCESCARACPYDAPQIDRERKVMTKCDGCFER........IADGRKP
S_enrypsychrophilus_strain_YLB-08_FM038_RS01530	96	.GIVAVHDDICIGCNSCAEACPYDAPQLDKTRGKMTKCDGCFER........LEFGLKP
S_enrypsychrophilus_strain_YLB-08_FM038_RS23495	91	RGLVLVHDDICIGCNACAQACPYDAPQMDTERSKMTKCDGCQDR........LNVGLKP
S_enrypsychrophilus_strain_YLB-08_FM038_RS23530	89	DGLVMVHDDICIGCSSCAQACPYDAPQLDEARGKMTKCDGCIDR........LNAGKQP
S_sp.YLB_09_FS418_RS01250	108	DGLVHIASELCIGCESCARACPYDAPQIDRERKVMTKCDGCFER........IADGRKP
S_sp.YLB_09_FS418_RS01515	96	.GIVAVHDDICIGCNSCAEACPYDAPQLDKTRGKMTKCDGCFER........LEFGLKP
S_sp.YLB_09_FS418_RS02870	108	DGLVHIASELCIGCESCARACPYDAPQIDRERKVMTKCDGCFER........IADGRKP
S_sp.YLB_09_FS418_RS03135	96	.GIVAVHDDICIGCNSCAEACPYDAPQLDKTRGKMTKCDGCFER........LEFGLKP
S_sp.YLB_09_FS418_RS25025	91	RGLVLVHDDICIGCNACAQACPYDAPQMDTERSKMTKCDGCQDR........LNVGLKP
S_sp.YLB_09_FS418_RS25060	89	DGLVMVHDDICIGCSSCAQACPYDAPQLDEARGKMTKCDGCIDR........LNAGKQP
S_sp._WPAGA9__IGB07_RS19230	109	DGLVLVAEELCIACESCSRACPYDAPQLDQERKVMTKCDGCYDR........LAEGRKP
S_sp._ARC9_LZ_GUY17_RS19095	108	DGLVLVQESLCIGCESCSRACPYDAPQIDIERKVMTKCDGCFDR........LAEGKKP
S_psychromarinicola_strain_M2_EGC80_RS00655	93	DGLVHVAADLCIGCESCARACPYDAPQIDKDRKVMTKCDGCFER........LAEGKQP
S_psychromarinicola_strain_M2_EGC80_RS06750	108	DGLVLVQESLCIGCESCSRACPYDAPQIDIERKVMTKCDGCSDR........LAEGKKP
S_psychromarinicola_strain_M2_EGC80_RS11435	93	NGLVKIEASLCIGCGSCSRACPYDAPQMDPVRKVMTKCDGCEDE........VSRGEKP
S_sp.Actino-trap-3__CXF80_RS10110	93	DGLVHVAADLCIGCESCARACPYDAPQIDKDRKVMTKCDGCFER........LAEGKQP
S_sp.Actino-trap-3__CXF80_RS15640	108	DGLVLVQESLCIGCESCSRACPYDAPQIDIERKVMTKCDGCSDR........LAEGKKP
S_livingstonensis_strain_LMG_19866_EGC82_RS20510	108	DGLVLVQESLCIGCESCSRACPYDAPQIDIERKVMTKCDGCFDR........LAEGKKP
Ferrimonas_lipolytica_strain_S7_HER31_RS14685	93	DGLVLIDDSVCIGCESCSRACPYDAPQIDADRGVMTKCDGCYER........LAEGKQP
Ferrimonas_lipolytica_strain_S7_HER31_RS15970	113	DGLVHIDQNVCIGCQSCERACPYDAPQFDNDRGVMTKCDGCYDHIDTDLSIPLASRRKP
Ferrimonas_lipolytica_strain_S7_HER31_RS04975	93	DGLVKIDQSVCIGCGSCAQACPYDAPQLDTARQVMTKCDGCSDR........LAQGKQP
S_sp.ISTPL2_CCLCJOKE_1_HUB64_RS13990	108	DGLVQVATELCIGCESCARACPYDAPQLDIERKVMTKCDGCSDR........LAQGKKP
S_sp.8A_M2897_RS06505	108	DGLVQVATELCIGCESCARACPYDAPQLDIERKVMTKCDGCSDR........LAEGKKP
S_woodyi_ATCC_51908_Swoo_0234	108	DGLVHVAQDLCIGCESCARACPYDAPQIDSERKVMTKCDGCFER........IAEGRKP
Ferrimonas_balerica_DSM_9799_Fbal_2475	95	TGLVSIDRGVCIGCASCARACPYDAPQLNQATGTMMKCDGCADR........LAEGKAP
Ferrimonas_balerica_DSM_9799_Fbal_3622	93	DGLVHVDQGICVGCEACARACPYDAPQIDKDRGVMTKCDGCYER........IAENRKP
S_frigidimarina_NCIMB_400_Sfri_3127	93	DGLVHVAADLCIGCESCARACPYDAPQIDKDRKVMTKCDGCFER........LAEGKQP
S_frigidimarina_NCIMB_400_Sfri_3683	107	DGLVLVHTDLCIGCNSCAEACPYDAPQLDPQRKVMTKCDGCYDR........LAQGKQP
S_sediminis_HAW-EB3_Ssed_0230	108	DGLVHVAQELCIGCESCARACPYDAPQIDRERKVMTKCDGCFER........IAEGRKP
S_sediminis_HAW-EB3_Ssed_0349	93	DGLVHVAADLCIGCESCARACPYDAPQIDKDRKVMTKCDGCYER........LAEGKQP
S_sediminis_HAW-EB3_Ssed_0358	89	DGLVMIHDDICIGCSSCEQACPYDAPQLDEARGKMTKCDACIDR........LNAGKQP
S_sediminis_HAW-EB3_Ssed_1308	93	DGLVHVSSDLCIGCSSCAKACPYDAPQLDPVKKVMVKCDGCFEL........IAEGRKP
S_sediminis_HAW-EB3_Ssed_1405	93	DGLVHIEASLCVGCQSCSRACPYDAPQFDSERGVMTKCDGCYDR........LEQGLNP
S_sediminis_HAW-EB3_Ssed_2922	93	DGLVHVASDLCIGCESCARACPYDAPQIDKARKVMTKCDGCFER........LAEGKNP
S_sp.KX20019_JK628_RS15595	108	DGLVHVAQDICIGCESCSRACPYDAPQIDRERKVMTKCDGCYER........IAEGRKP
S_sp.KX20019_JK628_RS20555	93	DGLVHVATDLCIGCESCARACPYDAPQIDKERKVMTKCDGCYER........LAEGKQP
Ferrimonas_sp.SCSIO_43195_J8Z22_RS00575	96	.GLVAIHSDICIGCASCAEACPYDAPQLEVSQQVMTKCDGCAER........LAYNLPP
Ferrimonas_sp.SCSIO_43195_J8Z22_RS11610	93	DGLVMVDPQVCIGCESCARACPYDAPQIDTAKGVMTKCDGCVER........IELGRKP
Pseudomonas_sp.SCT_BHD08_RS22420(idrB)	81	YPYPDVR.NILVKLGVAAGGGIGADKDIVAFNQQCTHMGGPLDG.........TYKAEH
Denitromonas_sp.IR12_I8J34_RS03785(idrB)	76	YPYPDVN.NILVKLGAEAGGGVGPQADVVAFNQQCTHMGGPLQG.........TYKAKH

```
                                                          .            .            .            .            .            .
S_oneidensis_MR-1_dmsB                                159 ICVDSCPLRALDFDTMDNLRAKYGEG..DGHIAPLPSPSITSP.NLIIKANRNGQPVG.
S_oneidensis_MR-1_SO_4357                             144 SCVMSCPQRALDFGLIAELKQQYGN...DSDISGLPSSSITHP.NLVLKVHAKS...GQ
S_sp._LZH-2__JM642_14185                              159 ICVDSCPLRALDFDTMDNLRAKYGEG..DGHIAPLPSPSITSP.NLIIKANRNGQPVG.
S_xiamenensis_NUITM-VS1_NUITMVS1_29530                159 ICVDSCPLRALDFDTMDNLRAKYGEG..DGHIAPLPSPSITSP.NLIIKANRNGQPVG.
S_putrefaciens_strain_FDAARGOS_681_FOB89_20610        159 ICVDSCPLRALDFDTMENLKAKYGEG..DGHIAPLPSASITSP.NLIIKANRNGQPIG.
S_sp._MR-4__Shewmr4_3678                              157 ICVNACPLRALDFDTMENLKAKYGEG..DGHIAPLPSSSITSP.NLIVKKNPNAQ....
S_fidelis_ATCC-BAA-318__L884__L884_RS0114165          159 VCVESCPLRALDFDTMDNLRAKYGNG..DGHIAPLPPESITSP.NLIIKANVNG...GQ
S_piezotolerans_WP3_SWP_RS15445                       159 VCVESCPLRALDFDTMDNLREKYGDG..DGHIAPLPSPSITSP.NLIIKANVNGSPAGS
S_schlegeliana_strain_JCM_JMA39_11561_RS09485         159 VCVESCPLRALDFDTMDNLRAKYGNG..DGHIAPLPPESITSP.NLIIKANVHG...GQ
S_marisflavi_strain_EP1_CFF01_RS01170                 158 ICVGSCPLRALDFDTMENLKAKYGNS..DGHVAPLPPESITSP.NLIIKANKHG....G
S_japonica_strain_KCTC_22435_SJ2017_RS00810           160 VCVESCPLRALDFDTTENIQAKYGSG..DGHIAPLPNPSITTP.NLIIKANRNGQPAGG
S_sp.SUN_WT4_FJQ87_RS02765                            160 TCVESCPLRALDFDTMENLEAKYGKG..DSHIAPLPSPAITSP.NLIIKANRHGQPAGS
S_sp.MBTL60-112-B2_K5Q73_RS09515                      159 VCVESCPLRALDFDTMDNLRAKYGNG..DGHIAPLPPESITSP.NLIIKANVNG...GQ
S_sp.MBTL60_112_B1_K5Q83_RS07015                      159 VCVESCPLRALDFDTMDNLRAKYGNG..DGHIAPLPPESITSP.NLIIKANVNG...GQ
S_enrypsychrophilus_strain_YLB-08_FM038_RS01265       159 VCVESCPLRALDFDTMDNLREKYGDG..DGHIAPLPSPSITSP.NLIIKANVNGRPSGS
S_enrypsychrophilus_strain_YLB-08_FM038_RS01530       146 FCVQSCSQRALDCGTVDELQAKYGGAPGVGHIAPLPDAGITQP.NLLIRGAKCSRPTGD
S_enrypsychrophilus_strain_YLB-08_FM038_RS23495       142 MCVASCIHRALDFGPIEELRQKYG...ATAEIAPLPPASVTKP.NLVINLHAQAQPAGS
S_enrypsychrophilus_strain_YLB-08_FM038_RS23530       140 MCVDSCIHRALDFGPIDELRAKYG...DTAEIAPLPSASITKP.NLILRVHPDGQPVGS
S_sp.YLB_09_FS418_RS01250                             159 VCVESCPLRALDFDTMDNLREKYGDG..DGHIAPLPSPSITSP.NLIIKANVNGRPSGS
S_sp.YLB_09_FS418_RS01515                             146 FCVQSCSQRALDCGTVDELQAKYGGAPGVGHIAPLPDAGITQP.NLLIRGAKCSRPTGD
S_sp.YLB_09_FS418_RS02870                             159 VCVESCPLRALDFDTMDNLREKYGDG..DGHIAPLPSPSITSP.NLIIKANVNGRPSGS
S_sp.YLB_09_FS418_RS03135                             146 FCVQSCSQRALDCGTVDELQAKYGGAPGVGHIAPLPDAGITQP.NLLIRGAKCSRPTGD
S_sp.YLB_09_FS418_RS25025                             142 MCVASCIHRALDFGPIEELRQKYG...ATAEIAPLPPASVTKP.NLVINLHAQAQPAGS
S_sp.YLB_09_FS418_RS25060                             140 MCVDSCIHRALDFGPIDELRAKYG...DTAEIAPLPSASITKP.NLILRVHPDGQPVGS
S_sp._WPAGA9__IGB07_RS19230                           160 VCVESCPLRALDFDTTENIQAKYGSG..DGHIAPLPNPSITTP.NLIIKANRNGQPAGG
S_sp._ARC9_LZ_GUY17_RS19095                           159 ICVESCPLRALDFDTMENLRAKYGNG..DGHFAPLPSESITSP.NLIVKASKKAQPVGA
S_psychromarinicola_strain_M2_EGC80_RS00655           144 SCVESCPMRAIDFGPMDELKAKYPGA.ISPAVAPLPQSSVTTP.NLLITPNRHSRPSGA
S_psychromarinicola_strain_M2_EGC80_RS06750           159 ICVESCPLRALDFDTMENLRAKYGNG..DGHFAPLPSESITSP.NLIVKGSRKAQPVGS
S_psychromarinicola_strain_M2_EGC80_RS11435           144 RCVAGCPQRALDFGVMDELKAKYPNA.TRGDIAPLPDPSITTP.SLLITRSRVGRGTGS
S_sp.Actino-trap-3__CXF80_RS10110                     144 SCVESCPMRAIDFGPMDELKAKYPGA.ISPAVAPLPQSSVTTP.NLLITPNRHSRPSGA
S_sp.Actino-trap-3__CXF80_RS15640                     159 ICVESCPLRALDFDTMENLRAKYGNG..DGHFAPLPSESITSP.NLIVKGSRKAQPVGS
S_livingstonensis_strain_LMG_19866_EGC82_RS20510      159 ICVESCPLRALDFDTMENLRAKYGNG..DGHFAPLPSESITSP.NLIVKASKKAQPVGA
Ferrimonas_lipolytica_strain_S7_HER31_RS14685         144 TCIESCPLRAMEFGPIDELRAKYGE...AADINPLPSSSTTNP.NLVIKVNSRN...DP
Ferrimonas_lipolytica_strain_S7_HER31_RS15970         172 HCVESCPLRAIDFGTIDELRALYGS...NADIQGLPSSSITDP.NLIIKVNPVS...GK
Ferrimonas_lipolytica_strain_S7_HER31_RS04975         144 YCVASCTQRALDFGTVDEMISKYPNA.ARADTAPLPSVSITTP.NLFISANPNAKASGS
S_sp.ISTPL2_CCLCJOKE_1_HUB64_RS13990                  159 ICVDSCPLRALDFDTMENLKAKYGEG..DGHIAPLPSASITSP.NLIIKANRNGQPIG.
S_sp.8A_M2897_RS06505                                 159 ICVDSCPLRALDFDTMDNLRAKYGEG..DGHIAPLPSPSITSP.NLIIKANRNGQPVG.
S_woodyi_ATCC_51908_Swoo_0234                         159 VCVESCPLRALDFDTMDNLRAKYGDG..DGHIAPLPSPSITSP.NLIIKANVNGRPAGS
Ferrimonas_balerica_DSM_9799_Fbal_2475                146 ICVAACPMRALDFGPMDELKQRYPHA.SIAAVAPLPDPGITSP.NLLIGANANAQPSGS
Ferrimonas_balerica_DSM_9799_Fbal_3622                144 ICVESCPLRALDFGPIDELRAKYGS...NADINPLPSSSITAP.NLCIKQN.RN...AQ
S_frigidimarina_NCIMB_400_Sfri_3127                   144 SCVESCPMRAIDFGPMEELMAKYPGA.ISPAVAPLPQSSITTP.NLLITPNRHSRPSGD
S_frigidimarina_NCIMB_400_Sfri_3683                   158 ICVGSCPLRALDFDTMENLRAKYGNG..DGHIAPLPPQSITSP.NLIIKANKNG....G
S_sediminis_HAW-EB3_Ssed_0230                         159 VCVESCPLRALDFDTMDNLRAKYGDG..DGHIAPLPSPSITSP.NLIIKANVNGRPAGS
S_sediminis_HAW-EB3_Ssed_0349                         144 SCVESCPMRAIDFGPMDELKAKYPGA.ISPDVAPLPESSVTTP.NLLITPNRHTRPSGA
S_sediminis_HAW-EB3_Ssed_0358                         140 MCVDSCIHRALDFGPIDELRAKYG...DTAEIAPLPSASITKP.NLILRVHPDGQPVGS
S_sediminis_HAW-EB3_Ssed_1308                         144 LCVEGCPQRALDFGLIEDLKAKYPGS.SIANVAPLPQASITSP.NLLISTNRLSRPSGS
S_sediminis_HAW-EB3_Ssed_1405                         144 TCVDSCPLRALEFGPIDELRNKHGD...NADIQPLPSSDITSP.NLIIKVN.RN...AQ
S_sediminis_HAW-EB3_Ssed_2922                         144 TCVESCPMRAIDFGTMDALKEKYPDA.VKPNFAPLPSSSITSP.NLLMKPNRHARASGS
S_sp.KX20019_JK628_RS15595                            159 VCVESCPMRALDFDTMENLTDKYGDG..DGHIAPLPSPSITSP.NLIIKANVNGSPAGS
S_sp.KX20019_JK628_RS20555                            144 SCVESCPMRAIDFGPMEELKAKYPAA.INPDVAPLPQSSVTTP.NLLITPNRHSRPSGA
Ferrimonas_sp.SCSIO_43195_J8Z22_RS00575               146 LCVQACSQRALDFGRVSDLQAKYGGLVGASHCAPLPSPSQTKP.NLLIRLSPNHQPVGS
Ferrimonas_sp.SCSIO_43195_J8Z22_RS11610               144 VCVESCPMRALDFGTMDELRARYPDA.VQPDIAPLPGSGITHP.NLLIKANRHARPSGS
Pseudomonas_sp.SCT_BHD08_RS22420(idrB)                130 QILGPCPLHLTTFDLTRHGMVASGHA.....TESLPQIVLEVQGDDIYAIGVLGLVYGF
Denitromonas_sp.IR12_I8J34_RS03785(idrB)              125 QALGPCPLHLTTFDLTRHGMVISGHA.....TESLPQIVLEVRGDDIYAVGVQGLIYGY
```

```
                                                                 .
S_oneidensis_MR-1_dmsB                               214  GSGQLLNPAEV
S_oneidensis_MR-1_SO_4357                            196  .RGEIINITEV
S_sp._LZH-2__JM642_14185                             214  GSGQLLNPAEV
S_xiamenensis_NUITM-VS1_NUITMVS1_29530               214  GSGQLLNPAEV
S_putrefaciens_strain_FDAARGOS_681_FOB89_20610       214  GSGQLLNPAEV
S_sp._MR-4__Shewmr4_3678                             209  SGGQVLNWSEI
S_fidelis_ATCC-BAA-318__L884__L884_RS0114165         212  G.GDILNPTEV
S_piezotolerans_WP3_SWP_RS15445                      215  GEGSILNPSEV
S_schlegeliana_strain_JCM_JMA39_11561_RS09485        212  G.GDILNPTEV
S_marisflavi_strain_EP1_CFF01_RS01170                210  RQGEILNFAEV
S_japonica_strain_KCTC_22435_SJ2017_RS00810          216  GAGNILNPKEV
S_sp.SUN_WT4_FJQ87_RS02765                           216  NMGRILNLSEV
S_sp.MBTL60-112-B2_K5Q73_RS09515                     212  G.GDILNPTEV
S_sp.MBTL60_112_B1_K5Q83_RS07015                     212  G.GDILNPTEV
S_enrypsychrophilus_strain_YLB-08_FM038_RS01265      215  GEGQILNLSEV
S_enrypsychrophilus_strain_YLB-08_FM038_RS01530      204  TNGSVLNPNEV
S_enrypsychrophilus_strain_YLB-08_FM038_RS23495      197  TAGTVINPNEC
S_enrypsychrophilus_strain_YLB-08_FM038_RS23530      195  GAGSVVNPNEV
S_sp.YLB_09_FS418_RS01250                            215  GEGQILNLSEV
S_sp.YLB_09_FS418_RS01515                            204  TNGSVLNPNEV
S_sp.YLB_09_FS418_RS02870                            215  GEGQILNLSEV
S_sp.YLB_09_FS418_RS03135                            204  TNGSVLNPNEV
S_sp.YLB_09_FS418_RS25025                            197  TAGTVINPNEC
S_sp.YLB_09_FS418_RS25060                            195  GAGSVVNPNEV
S_sp._WPAGA9__IGB07_RS19230                          216  GAGNILNPKEV
S_sp._ARC9_LZ_GUY17_RS19095                          215  STGRILNVREV
S_psychromarinicola_strain_M2_EGC80_RS00655          201  TDGEVLNYAEV
S_psychromarinicola_strain_M2_EGC80_RS06750          215  SMGRILNVREV
S_psychromarinicola_strain_M2_EGC80_RS11435          201  TDGNIANITEV
S_sp.Actino-trap-3__CXF80_RS10110                    201  TDGEVLNYAEV
S_sp.Actino-trap-3__CXF80_RS15640                    215  SMGRILNVREV
S_livingstonensis_strain_LMG_19866_EGC82_RS20510     215  STGRILNVREV
Ferrimonas_lipolytica_strain_S7_HER31_RS14685        196  .NAQHLNAFEV
Ferrimonas_lipolytica_strain_S7_HER31_RS15970        224  GTASHLNPFEV
Ferrimonas_lipolytica_strain_S7_HER31_RS04975        201  AEGYIINLKEV
S_sp.ISTPL2_CCLCJOKE_1_HUB64_RS13990                 214  GSGQVLNHAEV
S_sp.8A_M2897_RS06505                                214  GSGQLLNPAEV
S_woodyi_ATCC_51908_Swoo_0234                        215  GEGQILNFSEV
Ferrimonas_balerica_DSM_9799_Fbal_2475               203  TLGRVTNEREV
Ferrimonas_balerica_DSM_9799_Fbal_3622               195  AGGEVLNWFEV
S_frigidimarina_NCIMB_400_Sfri_3127                  201  TDGEVLNYAEV
S_frigidimarina_NCIMB_400_Sfri_3683                  210  RQGEVLNYSEV
S_sediminis_HAW-EB3_Ssed_0230                        215  GEGQILNLSEV
S_sediminis_HAW-EB3_Ssed_0349                        201  TDGEVLNYSEV
S_sediminis_HAW-EB3_Ssed_0358                        195  GAGSVVNPNEV
S_sediminis_HAW-EB3_Ssed_1308                        201  SEGKIENLTEV
S_sediminis_HAW-EB3_Ssed_1405                        195  PGGEILNEFEV
S_sediminis_HAW-EB3_Ssed_2922                        201  IDGVVLNHSEV
S_sp.KX20019_JK628_RS15595                           215  GEGRILNPSEV
S_sp.KX20019_JK628_RS20555                           201  TDGDVLNYSEV
Ferrimonas_sp.SCSIO_43195_J8Z22_RS00575              204  TAGKVINIREL
Ferrimonas_sp.SCSIO_43195_J8Z22_RS11610              201  LDGQVLNWAEV
Pseudomonas_sp.SCT_BHD08_RS22420(idrB)               184  DSMNDVQPA..
Denitromonas_sp.IR12_I8J34_RS03785(idrB)             179  SSNRAGR....
```
