# Supplementary material for: Global occurrence of the bacteria with capability for extracellular reduction of iodate
Source: Front Microbiol. 2022 Nov 25;13:1070601. doi: 10.3389/fmicb.2022.1070601 (PMC9732548; doi:10.3389/fmicb.2022.1070601)
Supplement: Supplementary file 9 [file Data_Sheet_3.PDF]

```
S_oneidensis_MR-1_mtrC                              1  MMNAQKSK...IALLLAASAVTMALTGCGG...SDGNNGNDGSDGGEPAGSIQTLNLDI
S_oneidensis_MR-1_mtrF                              1  MNKFASFT....TQYSLMLLIATLLSACGG...SDGDDGSPGEPGKPPAMTISSLNISV
S_sp._LZH-2_JM642_12750                             1  MMNAKKSK...FALLLAASAVTMALTGCGG...SDGNDGKPGEPGGEPAPAIQKLNFTF
S_sp._LZH-2_JM642_12740                             1  MNKFAKLT....TQYSLMLLLATLVSACGG...NDGNDGSPGEPGKPPAITITSLNIMV
S_xiamenensis_NUITM-VS1_NUITMVS1_26680              1  MMNAKKSK...FALLLAASAVTMALTGCGG...SDGNDGKPGEPGGEPAPAIQKLNFTF
S_xiamenensis_NUITM-VS1_NUITMVS1_26650              1  MNKFAKLT....TQYSLMLLLATLVSACGG...NDGNDGSPGEPGKPPAITITSLNIMV
S_sp._MR-4_Shewmr4_2510                             1  MMNAQKSK...IALLLAASVVTVALTGCGG...SDGNDGKPGEPGGEPAGSIHKLNLDI
S_sp._MR-4_Shewmr4_2508                             1  MNKFARFS....TQLSLLLALTTLLTACGG...SDGNDGDPGEPGKPPAMTITSLNIMV
S_putrefaciens_strain_FDAARGOS_681_FOB89_16330      1  MMNAYKSK...IALLLAASAVSMALVGCGG...SDGNDGNPGETGGEPAGAIQTLNFNF
S_fidelis_ATCC-BAA-318_L884_L884_RS0114715          1  MMNVKKSK...IALLLAAGAVSMALTGCGG...DDGNDGNNGKPGGPAAEYINTLNLKV
S_fidelis_ATCC-BAA-318_L884_L884_RS0114740          1  ...........MNKALISLLFVSSLAACSG...DDGTPGNPGEPGGPPASEINALNIEV
S_piezotolerans_WP3_SWP_RS14705                     1  MMNVKKSK...IALLLAAGAVSMALTGCGG...DDGTDGNPGNPGGPAADYINTLNLKV
S_piezotolerans_WP3_SWP_RS14685                     1  MKLITQS....RLHKSIPLGLIAMLAACGG...DDGSPGNPGEPGGPPASVINELNITI
S_schlegeliana_strain_JCM_11561_JMA39_RS08985       1  MMNTNKSK...IALLLAAGAVSMALTGCGG...DDGNDGNNGKPGGPAADVINVLNLKV
S_schlegeliana_strain_JCM_11561_JMA39_RS08965       1  MKRVTQS....LAIKSIPLMLIAMLAGCSG...DDGNPGNPGEPGGPPASEINSLDITV
S_marisflavi_strain_EP1_CFF01_RS06660               1  MMNVQHNK...FKLLLAAGAVSMALTGCGG...SDGKDGNPGNPGGPAADAIKVLHLDV
S_marisflavi_strain_EP1_CFF01_RS06680               1  MMKMNNP.....ICYVALLGLMTLLSACGG...DDGKPGNPGEPGGPPAMVIDDLHITF
S_sp.SUN_WT4__FJQ87_RS07980                         1  MMNTQIKK...IALLIASSMLSVGLAGCGS....DGDNGSTGKPGGPAANTVAQLKLDV
S_sp.MBTL60-112-B2_K5Q73_RS09070                    1  MMNTNKSK...IALLLAAGAVSMALTGCGG...DDGNDGDNGNPGGPAADVINVLNLKV
S_sp.MBTL60-112-B2_K5Q73_RS09050                    1  MKRVTQS....LAARMFPLALLAMLAGCGG...DDGSPGNPGEPGGPPATEINSLNITV
S_sp.MBTL60_112_B1_K5Q83_RS07460                    1  MMNTNKSK...IALLLAAGAVSMALTGCGG...DDGNDGDNGNPGGPAADVINVLNLKV
S_sp.MBTL60_112_B1_K5Q83_RS07480                    1  MKRVTQS....LAARMFPLALLAMLAGCGG...DDGSPGNPGEPGGPPATEINSLNITV
S_enrypsychrophilus_strain_YLB-08_FM038_RS07470     1  MMNVKNNK...FALLLAASAVSLALTGCGG...DDGNSGEDGNPGGPAANVVNVLNLDV
S_enrypsychrophilus_strain_YLB-08_FM038_RS07490     1  MKLITNIKSMLSKKSVSLMTLAALVAACGS...DDGEPGNPGEPGGPPASDIASLIVEV
S_sp.YLB_09_FS418_RS09045                           1  MMNVKNNK...FALLLAASAVSLALTGCGG...DDGNSGEDGNPGGPAANVVNVLNLDV
S_sp.YLB_09_FS418_RS09065                           1  MKLITNIKSMLSKKSVSLMTLAALVAACGS...DDGEPGNPGEPGGPPASDIASLIVEV
S_sp._WPAGA9_IGB07_RS05190                          1  MMNKKNSK...IALMLVASAVSVALTGCGSD.GDDGKDGTPGLPGGTPAEAIEQLNLEV
S_sp._ARC9_LZ_GUY17_RS06940                         1  MMNKKYTK...MALLLAMSAS.MTLVGCGG..GDDGEAGNPGNPGGEPAEAIKTLNLDI
S_psychromarinicola_strain_M2_EGC80_RS12750         1  MMNKKYTK...MALLLAMSAS.MTMVGCGG..GDDGEAGNPGNPSGEPAAAINTLNLDI
S_sp.Actino-trap-3_CXF80_RS00760                    1  MMNKKYTK...MALLLAMSAS.MTMVGCGS..GDDGEAGNPGNPSGEPAAAINTLNLDI
S_livingstonensis_strain_LMG_19866_EGC82_RS08000    1  MMNKKYIK...KALLLAMSAS.MTLVGCGG..GDDGVAGNPGTPGGEPAEAIKTLNLDI
Ferrimonas_lipolytica_strain_S7_HER31_RS03430       1  .MTTQKSH...MRLTALAALVAAALTGCSD..GSDGNSGEPGRPGGEPAMTITDLNLAV
S_sp.ISTPL2_CCLCJOKE_1_HUB64_RS04610                1  MMNAQTTK...IALLLAASAVTMALAGCGG...SDGNDGNPGEPGGEPAPAIQTLNFAF
S_sp.ISTPL2_CCLCJOKE_1_HUB64_RS04620                1  MNKFAKIR....TQFSLMLALVSLLSACGG...DNGQDGNPGEPAKPPALTITSLNIIV
S_japonica_strain_KCTC_22435_SJ2017_RS07855         1  MMNKKNSK...IALMLVASAVSVALTGCGSD.GDDGKDGNPGLPGGTPAEAIEQLNLEV
S_sp.8A_M2897_RS07845                               1  MMNAKKSK...FALLLAASAVTMALTGCGG...SDGNDGKPGEPGGEPAPAIQKLNFTF
S_sp.8A_M2897_RS07855                               1  MNKFAKLT....TQYSLMLLLTTLVSACGG...NDGNDGSPGEPGKPSAITITSLNIMV
S_woodyi_ATCC_51908_Swoo_3125                       1  MMNVQNNK...FALLLAASAVSMALTGCGG...SDGNDGDSGKPGGPAAEVVNVLNLDV
Ferrimonas_balerica_DSM_9799_Fbal_1357              1  MMTNNKMQ...WRLTACAAIMATALVGCGDD.GNDGNDGDPGRPGGNPAETIAQLNLEV
Ferrimonas_balerica_DSM_9799_Fbal_1362              1  MKTGSDLSS..LRLLLAGVMALSLLSACKDG..NDGAQGPSGPDTPPPAQEVTEINANI
S_sediminis_HAW-EB3_Ssed_1525                       1  MMNVKNNK...FALLLAASAVSMALTGCGG...SDGDDGNNGNPGGPVAEYINTLNLDV
S_sediminis_HAW-EB3_Ssed_1528                       1  MMNKSTKSNTVSLYRLIPLTLTLLLAACGSG..DDGAPGNPGEPGGPPASDISTLMVEV
S_sp.KX20019_JK628_RS15030                          1  MMNVKKSK...IALLLAASAVSMALTGCGG...DDGTDGNPGNPGGPAADYINTLNLKV
Ferrimonas_sp.SCSIO_43195_J8Z22_RS15465             1  MMMMNKPN...WRLLATAAVVSAALTACGGDDGNDGNSGEDGRPGGEPATSVSTLNLKV
```

```
S_oneidensis_MR-1_mtrC                             54  TKVSYE.NGAPMVTVFATNEADMPVIGLANLEIKKALQLIPEGATGPGNSANWQGLG..
S_oneidensis_MR-1_mtrF                             53  DKVAIS.DGIAQVDYQVSNQENQAVVGIPSATFI.AAQLLPQGATGAGNSSEWQHFTSE
S_sp._LZH-2_JM642_12750                            54  NKSVVT.NGVPSVEFTVTNEDDLPVVGLQKMRFA.AAQLLPQGATGAGNASQWQYFGDE
S_sp._LZH-2_JM642_12740                            53  DKVTVT.DGIAQVDYQVSNQDDEAVVGIPSATFI.AAQLLPQGATGAGNSSEWQHFTSE
S_xiamenensis_NUITM-VS1_NUITMVS1_26680             54  NKSVVT.NGVPSVEFTVTNEDDLPVVGLQKMRFA.AAQLLPQGATGAGNASQWQYFGDE
S_xiamenensis_NUITM-VS1_NUITMVS1_26650             53  DKVTVT.DGIAQVDYQVSNQDDEAVVGIPSATFI.AAQLLPQGATGAGNNSEWQHFTSE
S_sp._MR-4_Shewmr4_2510                            54  TKVNYE.NGSPVVTVFATNEDDLPIVGLTNLEIKKALQLIPQGATGPGNSANWQGLG..
S_sp._MR-4_Shewmr4_2508                            53  DKVAVT.DGIAQVDYQVSNQDDEAVVGIPSATFI.AAQLLPQGATGAGNSSEWQHFTSE
S_putrefaciens_strain_FDAARGOS_681_FOB89_16330     54  EKALIK.DGLPSLQFRVTNEDDMPVVGLQYFKFY.AEQLVPQGATGAGDASKWQYLIDE
S_fidelis_ATCC-BAA-318_L884_L884_RS0114715         54  TDVTYA.DGIPTVNVFATNEEDLPVVGLQDLGIT.GAQLTPQGATGAGNSAQWTRTARV
S_fidelis_ATCC-BAA-318_L884_L884_RS0114740         46  SEVVFS.SEIATVNYRITNENDEPVVGVPSATYL.AAQLLPQGFTNAGDASQWQYFTSE
S_piezotolerans_WP3_SWP_RS14705                    54  TDVTYT.DGQPTVNVFATNEEDLPVIGLQDLGIT.AAQLTPQGATGAGNSAQWTRTARV
S_piezotolerans_WP3_SWP_RS14685                    53  KDVAFD.TGIATVNYRVTNEEDEPVVGISSATYL.AAQLLPMGYTNAGNASQWQYFTSE
S_schlegeliana_strain_JCM_11561_JMA39_RS08985      54  TDVTYA.DGVPTINVFATNEEDLPVVGLTALEVKKVVQLIPQGATGAGNSAEWQYIGSQ
S_schlegeliana_strain_JCM_11561_JMA39_RS08965      53  NEVLFD.SGVATVNYRITNEADEPVVGIPSATYI.AAQLLPQGYTNAGNASEWQYFTAE
S_marisflavi_strain_EP1_CFF01_RS06660              54  TKVDYD.NGIPTITVFATNEEDLPVIGLKDLEVKKVVQLLPQGATGAGNAAEWQFIGSQ
S_marisflavi_strain_EP1_CFF01_RS06680              52  DQVDME.GGVATVNYRVTNQNDEPVVGVPTATFI.AAQLLPQGYTNAGNSSQWQYFTSE
S_sp.SUN_WT4__FJQ87_RS07980                        53  TDVNHQ.GSESQVTVFATNEQDLPVVGLKDLEIKKLSQLIPHGATGAGNSAAWQTLGAT
S_sp.MBTL60-112-B2_K5Q73_RS09070                   54  TDVTYA.DGVPTINVFATNEEDLPVVGLTALEVKKVVQLIPQGATGAGNSAEWQYIGSQ
S_sp.MBTL60-112-B2_K5Q73_RS09050                   53  NEVLFE.SGVATVNYRITNESDEPVVGIPSATYI.AAQLLPQGYTNAGNASQWQYFTSE
S_sp.MBTL60_112_B1_K5Q83_RS07460                   54  TDVTYA.DGVPTINVFATNEEDLPVVGLTALEVKKVVQLIPQGATGAGNSAEWQYIGSQ
S_sp.MBTL60_112_B1_K5Q83_RS07480                   53  NEVLFE.SGVATVNYRITNESDEPVVGIPSATYI.AAQLLPQGYTNAGNASQWQYFTSE
S_enrypsychrophilus_strain_YLB-08_FM038_RS07470    54  TKVTYQ.DGMPTVKVFATNEEDLPVVGLIDLGVV.AAQLTPQGATGAGNSAQWTRTARV
S_enrypsychrophilus_strain_YLB-08_FM038_RS07490    57  DDVALN.NGIATINYRVSNQDDEPVVGIPSSTYI.AAQLLPQGFTNAGNSSQWQYFTSE
S_sp.YLB_09_FS418_RS09045                          54  TKVTYQ.DGMPTVKVFATNEEDLPVVGLIDLGVV.AAQLTPQGATGAGNSAQWTRTARV
S_sp.YLB_09_FS418_RS09065                          57  DDVALN.NGIATINYRVSNQDDEPVVGIPSSTYI.AAQLLPQGFTNAGNSSQWQYFTSE
S_sp._WPAGA9_IGB07_RS05190                         56  LSVTYA.DNIPTVKVYATNEDDEAVVGLKDFSIENAAQLIPAGASGAGNSANWQKLGST
S_sp._ARC9_LZ_GUY17_RS06940                        54  TKVTYQ.DSKPTIEVFATNEKDLPVAGLKDFEIKKVVQLIPAGASGAGNSAQWQFIGSE
S_psychromarinicola_strain_M2_EGC80_RS12750        54  TKVTYQ.DGKPTIEVFATNEQDLPVAGLKDFEVKKVVQLIPVGASGAGNSAQWQFIGSE
S_sp.Actino-trap-3_CXF80_RS00760                   54  TKVTYQ.DGKPTIEVFATNEQDLPVAGLKDFEVKKVVQLIPVGASGAGNSAQWQFIGSE
S_livingstonensis_strain_LMG_19866_EGC82_RS08000   54  TKVTYQ.DSKPTIEVFATNEKDLPVAGLKDFEIKKVVQLIPAGASGAGNSAQWQFIGSE
Ferrimonas_lipolytica_strain_S7_HER31_RS03430      54  TDVQQV.DGQPQVTVLATNQDDESVVGLTDIEVK.QYELYPMGYAESGSSARWKSNGNN
S_sp.ISTPL2_CCLCJOKE_1_HUB64_RS04610               54  DKSVVT.NGIPSVEFTVTNENDLPVVGLQKMRFA.AAQLIPQGATGAGNASQWQYFGDE
S_sp.ISTPL2_CCLCJOKE_1_HUB64_RS04620               53  DKVAMT.DGIAQVDFQVSNQDDEAVIGIPSATFI.AAQLLPQGYTGAGNSSEWQHFTAE
S_japonica_strain_KCTC_22435_SJ2017_RS07855        56  LSVTYA.DNIPTVKVYATNEDDEAVVGLKDFSIENAAQLIPAGASGAGNSANWQKLGST
S_sp.8A_M2897_RS07845                              54  NKSVVT.NGVPSVEFTVTNEDDLPVVGLQKMRFA.AAQLLPQGATGAGNASQWQYFGDE
S_sp.8A_M2897_RS07855                              53  DKVTVT.DGIAQVDYQVSNQDDEAVVGIPSATFI.AAQLLPQGATGAGNSSEWQHFTSE
S_woodyi_ATCC_51908_Swoo_3125                      54  TKVTYQ.DGNPSITVFATNEEDLPVVGLTALEVKAVAQLLPQGATGAGDSAQWQKTGSE
Ferrimonas_balerica_DSM_9799_Fbal_1357             56  TKVEYV.DGQPQVTVLATNEEDESVVGLQSMEVK.NFQLYPQGFFQAGDSARWASAGSS
Ferrimonas_balerica_DSM_9799_Fbal_1362             56  ERYAINEDGQFTLRLLVTNQNDEGVAGLPSAVIL.AAQLLPTGFTGAGNSSEWRFLGSE
S_sediminis_HAW-EB3_Ssed_1525                      54  TKVTYV.DGMPSIEVFATNEEDLPVVGLKDFEVKKVAQLLPQGSTGAGDNAQWQVIGSQ
S_sediminis_HAW-EB3_Ssed_1528                      58  TGVTTD.AGIATVNYRVSNQDDEPVVGISTGTYI.AAQLLPQGYTNAGNSSQWQYFVSE
S_sp.KX20019_JK628_RS15030                         54  TDVTYD.NGQPTVNVFATNEEDLPIIGLQDLTLT.AAQLTPQGATGAGNSAQWTRTARL
Ferrimonas_sp.SCSIO_43195_J8Z22_RS15465            57  TDASFN.NGLPVFTLLATNEDDEAVVGLQGLKVN.VAQLLPAGHQVVGDPTKWQFAGDD
```

```
S_oneidensis_MR-1_mtrC                              110  ..........SSKSYVDNKNGSYTFKFDA...FDSNKVFNAQLTQRFNVVSAAG.....
S_oneidensis_MR-1_mtrF                              110  TCAAS.....CPGTFVDHKNGHYSYRFSATFNGMNGVTFLSDATQRLVIKIGGD.....
S_sp._LZH-2_JM642_12750                             111  TCDLA...STCPGTFVDKKNGHYSYTFKMNLTANAKITYNDQLAQRVLIRAYN.....T
S_sp._LZH-2_JM642_12740                             110  TCAAS.....CPGTFVDHKNGHYSYRFSATFNGMNGVTFVNDATQRVVIKLGGD.....
S_xiamenensis_NUITM-VS1_NUITMVS1_26680              111  TCDLA...STCPGTFVDKKNGHYSYTFKMNLTANAKITYNDQLAQRVLIRAYN.....T
S_xiamenensis_NUITM-VS1_NUITMVS1_26650              110  TCAAS.....CPGTFVDHKNGHYSYRFSATFNGMNGVTFVNDATQRLVIKLGGD.....
S_sp._MR-4_Shewmr4_2510                             110  ..........SSKNYVDNKNGSYTFKFDT...FDSNKVFNAQLTQRFNVVSAAG.....
S_sp._MR-4_Shewmr4_2508                             110  TCAAS.....CPGTFVDYKNGHYSYRFSATFNGMNGVSFLNDATQRVVIKLGGD.....
S_putrefaciens_strain_FDAARGOS_681_FOB89_16330      111  TCDLTPAVKKCTGTLVDHKNGTYSYDFGTNLKTSTRATYNGELAQRIVLNNYVRGSTPA
S_fidelis_ATCC-BAA-318_L884_L884_RS0114715          111  SG.........TDAYTDNKNGSYTFTFE.......PSEYDADLTQRFNVYAGGE...GS
S_fidelis_ATCC-BAA-318_L884_L884_RS0114740          103  SCSNS.....CDGDFVDHKNGQYSYTFSGAFNGMNNIAFMPGATQRVVIKVGGD.....
S_piezotolerans_WP3_SWP_RS14705                     111  SG.........TDSFTDNKDGSYTFTFE.......LDEYNEDMTQRFNVYAGGE...GS
S_piezotolerans_WP3_SWP_RS14685                     110  SCADV.....CDGEFVDHKNGQYSYTFSGAFNGMNDISFMSGGTQRVVIKIGGD.....
S_schlegeliana_strain_JCM_11561_JMA39_RS08985       112  K............EFVDQKNGNYSFTIP.......VEGYNSELTQRYNIIAS.....AS
S_schlegeliana_strain_JCM_11561_JMA39_RS08965       110  SCSSS.....CDGEFVDHKNGKYSYTFSGPFDGMNDITYIDGATQRVVIKVGGD.....
S_marisflavi_strain_EP1_CFF01_RS06660               112  K............AFTDHKDGNYTFTID.......VEGYNSELTQRYNVIAS.....AS
S_marisflavi_strain_EP1_CFF01_RS06680               109  NCNAS.....CPGSYVDHKNGNYSYTFSAAFDGMNDISYQAGATQRIVVKMGGD.....
S_sp.SUN_WT4__FJQ87_RS07980                         111  K............VFIDNKDGSYSFKVD.......VKNFDPALTQRFNIIAA.....AS
S_sp.MBTL60-112-B2_K5Q73_RS09070                    112  K............EFKDQKNGNYSFTIP.......VEGYNSELTQRYNVIAS.....AS
S_sp.MBTL60-112-B2_K5Q73_RS09050                    110  SCSSS.....CDGEFVDHKNGKYSYTFSGAFDGMNDITYINGATQRVVVKVGGD.....
S_sp.MBTL60_112_B1_K5Q83_RS07460                    112  K............EFKDQKNGNYSFTIP.......VEGYNSELTQRYNVIAS.....AS
S_sp.MBTL60_112_B1_K5Q83_RS07480                    110  SCSSS.....CDGEFVDHKNGKYSYTFSGAFDGMNDITYINGATQRVVVKVGGD.....
S_enrypsychrophilus_strain_YLB-08_FM038_RS07470     111  SG.........ADNYVDNKDGSYTFTLE.......LSEYNVDMTQRYNVTAGGE...GS
S_enrypsychrophilus_strain_YLB-08_FM038_RS07490     114  SCSSS.....CTGELVDHKNGKYSYTFSATFDGMNEMSYMSGATQRIVIKIGGD.....
S_sp.YLB_09_FS418_RS09045                           111  SG.........ADNYVDNKDGSYTFTLE.......LSEYNVDMTQRYNVTAGGE...GS
S_sp.YLB_09_FS418_RS09065                           114  SCSSS.....CTGELVDHKNGKYSYTFSATFDGMNEMSYMSGATQRIVIKIGGD.....
S_sp._WPAGA9_IGB07_RS05190                          114  S............TFVDNKNGSYDFTFESF....DTDTFNSELTQRFNVVAE.....AS
S_sp._ARC9_LZ_GUY17_RS06940                         112  K............AFTDHGNGNYSFTVN.......VEGFNPELTQRYNVIAS.....AS
S_psychromarinicola_strain_M2_EGC80_RS12750         112  K............TFTDHGNGNYSFNVN.......VEGFNPELTQRYNIIAS.....AS
S_sp.Actino-trap-3_CXF80_RS00760                    112  K............TFTDHGNGNYSFNVN.......VEGFNSELTQRYNIIAS.....AS
S_livingstonensis_strain_LMG_19866_EGC82_RS08000    112  K............AFTDHGNGNYSFTVN.......VEGFNPDLTQRYNVIAS.....AS
Ferrimonas_lipolytica_strain_S7_HER31_RS03430       111  S............AVVDQGNGYYTFTFAE.......LEQNAALTQRYNVVSEAG.....
S_sp.ISTPL2_CCLCJOKE_1_HUB64_RS04610                111  TCDVA...ATCPGTFVDKKNGHYSYTFSMNLTANTKVTYNEQLAQRVLIRAYN.....T
S_sp.ISTPL2_CCLCJOKE_1_HUB64_RS04620                110  TCAAT.....CPGSLTDHKNGHYSYTFSAAFNGMNGVTFMPEATQRMVVKIGGD.....
S_japonica_strain_KCTC_22435_SJ2017_RS07855         114  S............TFVDNKNGSYDFTFESF....DTDTFNSELTQRFNVVAE.....AS
S_sp.8A_M2897_RS07845                               111  TCDLA...STCPGTFVDKKNGHYSYTFKMNLTANAKITYNDQLAQRVLIRAYN.....T
S_sp.8A_M2897_RS07855                               110  TCAAS.....CLGTFVDHKNGHYSYRFSATFNGMNGVTFVNDATQRLVIKLGGD.....
S_woodyi_ATCC_51908_Swoo_3125                       112  K............VFVDHKNGNYTFDIA.......LEGYNPELTQRYNVIAS.....AS
Ferrimonas_balerica_DSM_9799_Fbal_1357              113  K............VYEDHGNGYYTFSFEG.......IEQNSDLTQRYNVLAGNN.....
Ferrimonas_balerica_DSM_9799_Fbal_1362              114  TCTPQG...ECPGVWTDFGNGFYDYQTQFSVNDANGVEYSADATQRLVVKVGGD.....
S_sediminis_HAW-EB3_Ssed_1525                       112  K............TFIDNQDGSYTFDIE.......VEGYDSALTQRYNVIAK.....AS
S_sediminis_HAW-EB3_Ssed_1528                       115  SCSSG.....CTGELIDHKNGSYSYTFSAAFDGLNDVSYQPGSTQRVMIKIGGD.....
S_sp.KX20019_JK628_RS15030                          111  KG.........ADKYTDNKDGSYTFTFE.......LEEYNADMTQRFNVHAGGE...GS
Ferrimonas_sp.SCSIO_43195_J8Z22_RS15465             114  SRN.......GAAEIVDQKNGYYTVTFTS....LDKAQLDPNFTRRLNIVSP.....AG
```

```
S_oneidensis_MR-1_mtrC                              151  KLADG.TTVPVAEMVEDFDGQ...GNAPQYTKNIVSHEVCASCHVEGE...KIYH....
S_oneidensis_MR-1_mtrF                              159  ALADGTVLPITNQHYDWQSSGN....MLAYTRNLVSIDTCNSCHSNL.....AFHG...
S_sp._LZH-2_JM642_12750                             162  PLPDG.TLVPNSNAFVDFTADT..GAEPGYSRKIVATESCNTCHQDLA...QVKHGG..
S_sp._LZH-2_JM642_12740                             159  ALADGTALPITNQHYDWQTTGN....TLAYTRNLITIETCNSCHNNL.....AFHG...
S_xiamenensis_NUITM-VS1_NUITMVS1_26680              162  PLPDG.TLVPNSNAFVDFTADT..GAEPGYSRKIVATESCNTCHQDLA...QVKHGG..
S_xiamenensis_NUITM-VS1_NUITMVS1_26650              159  ALADGTALPITNQHYDWQTSGN....TLAYTRNLITIETCNSCHNNL.....AFHG...
S_sp._MR-4_Shewmr4_2510                             151  KLPDG.TSVPVAEMVEDFDGQ...GNAPLYTKNIVSHEVCTTCHVEGE...KIYH....
S_sp._MR-4_Shewmr4_2508                             159  ALADGTALPITNQHYDWQTSGN....TLAYSRNLITIETCNSCHSNL.....AFHG...
S_putrefaciens_strain_FDAARGOS_681_FOB89_16330      170  PLPDG.TTLPVFTGIFDYMADT..GADATYSRKIVATESCNTCHDKVI...NAKH....
S_fidelis_ATCC-BAA-318_L884_L884_RS0114715          151  TLADGTTSVPRQEIVADFDGQ...GYQAKYTKNIVSHETCTNCHAEGKPLTTRHSS...
S_fidelis_ATCC-BAA-318_L884_L884_RS0114740          152  TLPDGTALPVTNQHFDWQDNGS....EPAYTRNLIEMQTCNTCHNDL.....AFHG...
S_piezotolerans_WP3_SWP_RS14705                     151  TLADGVTSVPRREMVADFDGQ...GYEAKYTKNIVSHETCTNCHAEGEPLTTRHSS...
S_piezotolerans_WP3_SWP_RS14685                     159  SLPDGTALPITNQHYDWQDKGD....TPAYTRNLIEMETCNSCHSDL.....AFHG...
S_schlegeliana_strain_JCM_11561_JMA39_RS08985       147  TLADGETAVPRTELSQDFSGE...GYEATYTKDVVATASCNSCHAEGK...KIYHG...
S_schlegeliana_strain_JCM_11561_JMA39_RS08965       159  SLPDGTALPITNQYLDWQDEGS....EPAYTRDILDIQSCNTCHDDL.....AFHGPAG
S_marisflavi_strain_EP1_CFF01_RS06660               147  TLLDGVTVVPRTEIAEDFSGE...GYEALYTKNVVDTASCNSCHAEGE...KIYHS...
S_marisflavi_strain_EP1_CFF01_RS06680               158  TLADGTSLPITNQHFDWQAETDGTSSEPAYSRNLIKIDTCNSCHNDL.....AFHR...
S_sp.SUN_WT4__FJQ87_RS07980                         146  TLQDGTTSVPRTEYTEDLASD...GSAPRYTKNVVATETCNTCHAEGV...KIYHG...
S_sp.MBTL60-112-B2_K5Q73_RS09070                    147  TLADGTTTVPRTELSQDFSGE...GYKATYTKDVVATASCNSCHAEGK...KIYHG...
S_sp.MBTL60-112-B2_K5Q73_RS09050                    159  TLPDGTDLPITNQHYDWQDNGS....EPAYTRNLIEMSTCNTCHNDL.....AFHG...
S_sp.MBTL60_112_B1_K5Q83_RS07460                    147  TLADGTTTVPRTELSQDFSGE...GYKATYTKDVVATASCNSCHAEGK...KIYHG...
S_sp.MBTL60_112_B1_K5Q83_RS07480                    159  TLPDGTDLPITNQHYDWQDNGS....EPAYTRNLIEMSTCNTCHNDL.....AFHG...
S_enrypsychrophilus_strain_YLB-08_FM038_RS07470     151  TLADGVTSVPRTEMVEDFSGE...GYEALYTKNIVSHETCTNCHAEGKPLTTRHSS...
S_enrypsychrophilus_strain_YLB-08_FM038_RS07490     163  SLPDGTVLPTTNQHFDWQDSG.....EAAYTRNLIVMETCNTCHDDL.....AFHG...
S_sp.YLB_09_FS418_RS09045                           151  TLADGVTSVPRTEMVEDFSGE...GYEALYTKNIVSHETCTNCHAEGKPLTTRHSS...
S_sp.YLB_09_FS418_RS09065                           163  SLPDGTVLPTTNQHFDWQDSG.....EAAYTRNLIVMETCNTCHDDL.....AFHG...
S_sp._WPAGA9_IGB07_RS05190                          152  TLLDGVTEVPVSEIVADFDGE...GYEALYTKNIVSHEACAACHAEGE...KIYHK...
S_sp._ARC9_LZ_GUY17_RS06940                         147  TLQDGVTGVPRTEITKDFDGE...GFEAKYTKNIVSGATCNTCHAKGE...SIYHS...
S_psychromarinicola_strain_M2_EGC80_RS12750         147  TLQDGVTGVPRTEISEDFDGE...GFEAKYTKNIVSGATCNTCHAKGE...SIYHS...
S_sp.Actino-trap-3_CXF80_RS00760                    147  TLQDGVTGVPRTEITEDFDGE...GFEAKYTKNIVSGATCNTCHAKGE...SIYHS...
S_livingstonensis_strain_LMG_19866_EGC82_RS08000    147  TLQDGVTGVPRTEITEDFDGE...GFEAKYTKNIVSGATCNTCHAEGE...SIYHS...
Ferrimonas_lipolytica_strain_S7_HER31_RS03430       146  TLADGTTEVPHNELSYDVDHA...GNPALYTKDVVSHDSCAACHTEGEALTRRHSK...
S_sp.ISTPL2_CCLCJOKE_1_HUB64_RS04610                162  PLPDG.TQVPNSNAFVDFTADT..GALPTYSRKIVATESCNTCHQDLA...NVKHGG..
S_sp.ISTPL2_CCLCJOKE_1_HUB64_RS04620                159  KLADNTPLPITNQHYDWQSSGS....NVAYTRNLVTIETCNSCHSNL.....AFHG...
S_japonica_strain_KCTC_22435_SJ2017_RS07855         152  TLLDGVTEVPVSEIVADFDGE...GYEALYTKNIVSHEACAACHAEGE...KIYHK...
S_sp.8A_M2897_RS07845                               162  PLPDG.TLVPNSNAFVDFTADT..GAEPSYSRKIVATESCNTCHQDLA...QVKHGG..
S_sp.8A_M2897_RS07855                               159  ALADGTALPITNQHYDWQTSGN....TLAYTRNLITIETCNSCHNNL.....AFHG...
S_woodyi_ATCC_51908_Swoo_3125                       147  TLQDGITTVPRTELSEDFSGD...GYQALYTKDIVSTQTCNTCHAQGE...KIYHG...
Ferrimonas_balerica_DSM_9799_Fbal_1357              148  TLPDG.TPVPRNEISEDFDGQ...GNDALYTKDVISHDACTACHVEGEPLTTRHSS...
Ferrimonas_balerica_DSM_9799_Fbal_1362              165  ALPDGTALPVVNTFVDFTPPNS....DPHYTRLIAATESCESCHTDLG...SVRHG...
S_sediminis_HAW-EB3_Ssed_1525                       147  TLLDGVTTVPQAEIAEDFSGE...GYEATYTKDIVSTASCNACHAEGE...KIYHS...
S_sediminis_HAW-EB3_Ssed_1528                       164  TLPDGSPLPTTNQHFDWSDSG.....EPAYTRDLIAIETCNSCHNDL.....AFHGPSG
S_sp.KX20019_JK628_RS15030                          151  TLADGVTSVPRREIVADFDGQ...GFEAKYTKNIVSHETCTNCHAEGEPLTTRHSD...
Ferrimonas_sp.SCSIO_43195_J8Z22_RS15465             157  TLIDGTTEVPNGQASFDYNAA...GAAADYTRNIVATDSCAACHGEGN...GIHHS...
```

S_oneidensis_MR-1_mtrC	199	..QATEVETCISCHTQEFADGRGKPHVAFSHLIHNVHNANKAWGKDN......KIPTVA
S_oneidensis_MR-1_mtrF	206	.GRYNQVETCVTCHN...SKKVSNAADIFPQMIHSKHLT...................G
S_sp._LZH-2_JM642_12750	213	..AYSDVNYCATCHT....AGKVGVGKEFNALVHAKHKDLTLG................
S_sp._LZH-2_JM642_12740	206	.GRYNQVETCVTCHN...SKKVSNPADIFPQMIHSKHLT...................G
S_xiamenensis_NUITM-VS1_NUITMVS1_26680	213	..AYSDVNYCATCHT....AGKVGVGKEFNALVHAKHKDLTLG................
S_xiamenensis_NUITM-VS1_NUITMVS1_26650	206	.GRYNQVETCVTCHN...SKKVSNPADIFPQMIHSKHLT...................G
S_sp._MR-4_Shewmr4_2510	199	..QATEVETCISCHTQEFADGRGKPHVAFSHLIHNVHNANKAWGKNN......KVPEVA
S_sp._MR-4_Shewmr4_2508	206	.GRYNQVETCVTCHN...SKKVSDPADIFPQMIHSKHLA...................G
S_putrefaciens_strain_FDAARGOS_681_FOB89_16330	219	..YTNDVNFCASCHT....PGRVKAGNEFNVLVHAKHKDLTLN................
S_fidelis_ATCC-BAA-318_L884_L884_RS0114715	204	...YYKEETCATCHSSSMST.....ESQWNHLIHNIHNTAKTFEDKY...GKEYTGEAA
S_fidelis_ATCC-BAA-318_L884_L884_RS0114740	199	.SKYNEVETCVTCHN...ADKVSNPDNVFAPMVHSKHLT...................G
S_piezotolerans_WP3_SWP_RS14705	204	...YYTQETCATCHTSKYG......ESQWNHLIHNIHNTAKTFEDKY...GKEYTGEAA
S_piezotolerans_WP3_SWP_RS14685	206	.SKYNEVETCVTCHN...TDKVSNPENVFAPMVHGKHLT...................G
S_schlegeliana_strain_JCM_11561_JMA39_RS08985	197	...YTSSETCAACHTQEMADSKGKPQVAYNHLIHNVHNNAKMYGRNM...DKS..ADTA
S_schlegeliana_strain_JCM_11561_JMA39_RS08965	209	KGKYNEVQACVTCHN...LAKVSNPGNVFAPMVHTKHLT...................G
S_marisflavi_strain_EP1_CFF01_RS06660	197	...YTEVESCVSCHTQEMADDKGKPQVAFGHLVHNVHNDAKMYGRN.....MEKSAETA
S_marisflavi_strain_EP1_CFF01_RS06680	209	.GNYNQIETCVTCHN...ADRVSNPDNIFPQMIHGKHLT...................G
S_sp.SUN_WT4__FJQ87_RS07980	196	...YTSLESCTTCHNDDMAKDKNKVQVGFNHLIHNVHNSNKMYGKNL.....DKSAETA
S_sp.MBTL60-112-B2_K5Q73_RS09070	197	...YTTSETCAACHTQEMADEKGKPQVAYNHLIHNVHNDAKMYGKNM...DKS..AETA
S_sp.MBTL60-112-B2_K5Q73_RS09050	206	.SKYDEVETCVTCHN...LTKVNNPGNVFAPMVHTKHLT...................G
S_sp.MBTL60_112_B1_K5Q83_RS07460	197	...YTTSETCAACHTQEMADEKGKPQVAYNHLIHNVHNDAKMYGKNM...DKS..AETA
S_sp.MBTL60_112_B1_K5Q83_RS07480	206	.SKYDEVETCVTCHN...LTKVNNPGNVFAPMVHTKHLT...................G
S_enrypsychrophilus_strain_YLB-08_FM038_RS07470	204	...YYTQETCATCHSSSMST.....ESQWNHLIHNIHNTAKTFEDKY...GKEYTGEAA
S_enrypsychrophilus_strain_YLB-08_FM038_RS07490	209	.SKYNEVETCVTCHS...EGKVSSNDNIFPQMIHAKHLT...................G
S_sp.YLB_09_FS418_RS09045	204	...YYTQETCATCHSSSMST.....ESQWNHLIHNIHNTAKTFEDKY...GKEYTGEAA
S_sp.YLB_09_FS418_RS09065	209	.SKYNEVETCVTCHS...EGKVSSNDNIFPQMIHAKHLT...................G
S_sp._WPAGA9_IGB07_RS05190	202	...ATTVETCITCHTQEWADGRGKPEVAFAHLVHNVHNSNKVWGRN......DYTAETA
S_sp._ARC9_LZ_GUY17_RS06940	197	...YTDLETCTSCHTNEMAIEKDKVQVEFSHLIHNVHNNAKMYGRNL.....DKSAEAA
S_psychromarinicola_strain_M2_EGC80_RS12750	197	...YTDLETCTSCHTNELAIEKDKVQDEFSHLIHNVHNNAKMYGRNM.....DKSAETA
S_sp.Actino-trap-3_CXF80_RS00760	197	...YTDLETCTSCHTNELAIEKDKVQDEFSHLIHNVHNNAKMYGRNM.....DKSAETA
S_livingstonensis_strain_LMG_19866_EGC82_RS08000	197	...YTDLETCTSCHTNELAIEKGKVQDEFSHLIHNVHNNAKMYGRNL.....DKSAEVA
Ferrimonas_lipolytica_strain_S7_HER31_RS03430	199	...YQEVETCINCHNETRMAGE...RSSFQHLVHFIHNDDQSLEDKN...GEIYDGVAA
S_sp.ISTPL2_CCLCJOKE_1_HUB64_RS04610	213	..AYSDVNYCATCHT....AGKVGVGKEFNVLVHAKHKDLTLG................
S_sp.ISTPL2_CCLCJOKE_1_HUB64_RS04620	206	.SRYNQIETCVTCHN...SKKVSNP.DIFPQMIHSKHLT...................S
S_japonica_strain_KCTC_22435_SJ2017_RS07855	202	...ATTVETCITCHTQEWADGRGKPEVAFAHLVHNVHNSNKVWGRN......DYTAETA
S_sp.8A_M2897_RS07845	213	..AYSDVNYCATCHT....AGKVGVGKEFNALVHAKHKDLTLG................
S_sp.8A_M2897_RS07855	206	.GRYNQVETCVTCHN...SKRVSNPADIFPQMIHSKHLT...................G
S_woodyi_ATCC_51908_Swoo_3125	197	...YTSAETCASCHTQEWAEGRGKPEVAFTHMVHNVHNSAKGYKADRTTGEFTKDAKKA
Ferrimonas_balerica_DSM_9799_Fbal_1357	200	...YFEAETCINCHNEDRMTGK...RASFQHLVHTIHNTAATFTDKN...DREYTGQAA
Ferrimonas_balerica_DSM_9799_Fbal_1362	214	.GRYTELETCTVCHA...DNRISNPANVLSGLAHSAHGQ...................S
S_sediminis_HAW-EB3_Ssed_1525	197	...YMTQETCATCHTKEYAEERGKPEIEWNHLVHNVHNSAKGYKADRDTGEFTKDAVTA
S_sediminis_HAW-EB3_Ssed_1528	213	FEKYNEVETCVTCHN...ADRVSNPDNIFPQMIHTKHLV...................G
S_sp.KX20019_JK628_RS15030	204	...YYTQETCATCHTSKYG......ESQWNHLIHNIHNTAKTFEDKY...GKEYTGEAA
Ferrimonas_sp.SCSIO_43195_J8Z22_RS15465	207	..NYIEPETCATCHDGLKAEG.GRTSRSFAVLVHDVHKVAAEKDENG.........KSE

S_oneidensis_MR-1_mtrC	250	QNIVQDNCQVCHVE.SDM....LTEAKNWSRIPTMEVCSSCHVD....IDFAAGKGHSQ
S_oneidensis_MR-1_mtrF	242	FPQSISNCQTCHAD.....NPDLADRQNWYRVPTMEACGACHTQ....INFPAGQGHPA
S_sp._LZH-2_JM642_12750	250	...SLESCQSCHTE.NEA....TPDWGNWSRIPTAATCGSCHST....VDFAAGKGHSQ
S_sp._LZH-2_JM642_12740	242	FPQSISNCQTCHVD.....NPDLAERQNWHRVPTMEACGACHTQ....INFPAGQGHPA
S_xiamenensis_NUITM-VS1_NUITMVS1_26680	250	...SLESCQSCHTE.NEA....TPDWGNWSRIPTAATCGSCHST....VDFAAGKGHSQ
S_xiamenensis_NUITM-VS1_NUITMVS1_26650	242	FPQSISNCQTCHID.....NPDLAERQNWHRVPTMEACGACHTQ....INFPAGQGHPA
S_sp._MR-4_Shewmr4_2510	250	QHIVQDNCQVCHVE.SDM....LTEAKNWSRIPTMEVCSSCHVD....IDFAAGKGHSQ
S_sp._MR-4_Shewmr4_2508	242	FPQSISNCQTCHVD.....NPDLAEAQNWHRVPTMEACGACHTQ....INFPAGQGHPA
S_putrefaciens_strain_FDAARGOS_681_FOB89_16330	256	...ALDSCQSCHAE.SDA....APDWSNWSRIPTAATCGSCHST....VDFAAGKGHSQ
S_fidelis_ATCC-BAA-318_L884_L884_RS0114715	252	EALIQNNCKSCHVA.PEEGSDELSEWGNWSSVPTMETCTSCHTG....IDFKAGKGHSQ
S_fidelis_ATCC-BAA-318_L884_L884_RS0114740	235	FPVSLADCQTCHVA.....DETLTENMNWARVPTMQACGSCHTN....IDFPSGQGHPA
S_piezotolerans_WP3_SWP_RS14705	251	EALIQNNCKSCHVE.PEADSGELTEWGNWSRIPTMETCTSCHTG....IDFEAGKGHSQ
S_piezotolerans_WP3_SWP_RS14685	242	FPGSLADCQTCHAP.....DETLTENMNWARVPTMAACGSCHTN....IDFPAGQGHPT
S_schlegeliana_strain_JCM_11561_JMA39_RS08985	248	NAIVQDKCTTCHVE.PTEGSDELGEWGNWSRVPTMETCTSCHVN....IDFKAGQGHSQ
S_schlegeliana_strain_JCM_11561_JMA39_RS08965	246	FPVSLADCQTCHAP.....DEELAQNMNWARVPTMEACGSCHTN....IDFPAGQGHPT
S_marisflavi_strain_EP1_CFF01_RS06660	248	HAIVQDNCQTCHVQ.SEE....LTEWGNWSRVPTMETCTSCHVN....VDFKAGKGHSQ
S_marisflavi_strain_EP1_CFF01_RS06680	245	FPGSLANCQTCHAQ.....DESLSEQMNWARVPTMETCGSCHTY....IDFKAGEGHPA
S_sp.SUN_WT4__FJQ87_RS07980	247	HNLIQDNCQACHIP.PQADDNGLTEWNNWSRVPTMETCTSCHTT....IDFKAGKGHSQ
S_sp.MBTL60-112-B2_K5Q73_RS09070	248	NGIVQDNCTTCHVA.PTEDSNELTEWGNWSSVPTMETCTSCHVN....IDFQAGQGHSQ
S_sp.MBTL60-112-B2_K5Q73_RS09050	242	FPVSLADCQTCHAA.....DEALTENMNWARVPTMEACGSCHTN....IDFPAGQGHPT
S_sp.MBTL60_112_B1_K5Q83_RS07460	248	NGIVQDNCTTCHVA.PTEDSNELTEWGNWSSVPTMETCTSCHVN....IDFQAGQGHSQ
S_sp.MBTL60_112_B1_K5Q83_RS07480	242	FPVSLADCQTCHAA.....DEALTENMNWARVPTMEACGSCHTN....IDFPAGQGHPT
S_enrypsychrophilus_strain_YLB-08_FM038_RS07470	252	EALIQNNCKSCHVE.PDADSGELAEWGNWSRVPTMETCTSCHTN....IDFKAGKGHSQ
S_enrypsychrophilus_strain_YLB-08_FM038_RS07490	245	FPGSLADCQTCHVA.....DETLTENMNWARVPGMEACGSCHTD....INFPAGEGHPA
S_sp.YLB_09_FS418_RS09045	252	EALIQNNCKSCHVE.PDADSGELAEWGNWSRVPTMETCTSCHTN....IDFKAGKGHSQ
S_sp.YLB_09_FS418_RS09065	245	FPGSLADCQTCHVA.....DETLTENMNWARVPGMEACGSCHTD....INFPAGEGHPA
S_sp._WPAGA9_IGB07_RS05190	252	HAIVQDNCQSCHIE.SEE....LTEWGNWTRIPTMETCTSCHTN....IDFKAGEGHSQ
S_sp._ARC9_LZ_GUY17_RS06940	248	HAIVQDNCTTCHVD.SEQ....LSESNNWSRIPTMEACSSCHTD....IDFKTGQGHPQ
S_psychromarinicola_strain_M2_EGC80_RS12750	248	HAIVQDNCTTCHVD.SEQ....LSESSNWTRIPTMETCSSCHTD....IDFKTGQGHSQ
S_sp.Actino-trap-3_CXF80_RS00760	248	HAIVQDNCTTCHVD.SEQ....LSESSNWTRIPTMETCSSCHTD....IDFKIGQGHSQ
S_livingstonensis_strain_LMG_19866_EGC82_RS08000	248	HAIVQDNCTTCHVN.SEQ....LSESNNWSRIPTMEACSSCHTD....IDFKTGQGHPQ
Ferrimonas_lipolytica_strain_S7_HER31_RS03430	249	EALIGNNCQACHIVTEE.....LAESGNWNRVPTKETCSACHNDGYDADKNTIYSRHLE
S_sp.ISTPL2_CCLCJOKE_1_HUB64_RS04610	250	...SLESCQSCHAA.NEA....TPDWSNWSRIPTAASCGSCHSA....VDFAAGKGHSQ
S_sp.ISTPL2_CCLCJOKE_1_HUB64_RS04620	241	FPQPISDCQTCHVD.....KAELTEQQNWRRVPTMQACGACHTQ....INFPAGEGHPV
S_japonica_strain_KCTC_22435_SJ2017_RS07855	252	HAIVQDNCQSCHIE.SEE....LTEWGNWTRIPTMETCTSCHTN....IDFKAGEGHSQ
S_sp.8A_M2897_RS07845	250	...SLESCQSCHTE.NEA....TPDWGNWSRIPTAATCGSCHST....VDFAAGKGHSQ
S_sp.8A_M2897_RS07855	242	FPQSISNCQTCHVD.....NPDLAERQNWHRVPTMEACGACHTQ....INFPAGQGHPA
S_woodyi_ATCC_51908_Swoo_3125	253	HALLQDNCKTCHVA.PAEDSDELSEWGNWSRVPTMETCTSCHVN....IDFKAGKGHSQ
Ferrimonas_balerica_DSM_9799_Fbal_1357	250	EALLQNNCQACHVASEE.....LAEWGNWTRVPTKETCSACHNNGYDG.KDVVHLRHLA
Ferrimonas_balerica_DSM_9799_Fbal_1362	250	ELANFLNCETCHTG.....GEALPESDNWALFPSQLACGSCHSN....IDFVAGVGHPA
S_sediminis_HAW-EB3_Ssed_1525	253	QHIVQNDCTNCHVA.PEDGTEGLTEWGNWTRVPTMETCTSCHVN....IDFEAGKGHSQ
S_sediminis_HAW-EB3_Ssed_1528	250	FPGELANCQTCHAS.....DETLTENMNWARVPGMEACGSCHTN....IDFPAGEGHPA
S_sp.KX20019_JK628_RS15030	251	EHLIQNNCKSCHVE.PEADSGELTEWGNWSRIPTMETCTSCHVN....IDFVAGKGHSQ
Ferrimonas_sp.SCSIO_43195_J8Z22_RS15465	254	YPTELSSCNVCHETSAPAEELAITEWGNWSAVPSKENCASCHAD....NNHIM.....E

```
S_oneidensis_MR-1_mtrC                              300  QLDNSNCIACHNSD......WTAELHTAKTTATKNLINQYGIETTSTINT...ETKAAT
S_oneidensis_MR-1_mtrF                              292  QTDNSNCVACHNAD......WTANVHSN..AAQTSALAQFNASISSASM.DANGTITVA
S_sp._LZH-2_JM642_12750                             297  QLDNSNCIACHNST......WTAELHTAKTADKKAVIAQLGMS.ATLAAQ...ADNTAV
S_sp._LZH-2_JM642_12740                             292  QADNSNCVACHNAD......WTASVHGN..EDQMAALAQFSPSISSASM.DANGTVTVA
S_xiamenensis_NUITM-VS1_NUITMVS1_26680              297  QLDNSNCIACHNST......WTAELHTAKTADKKAVIAQLGMS.ATLAAQ...ADNTAV
S_xiamenensis_NUITM-VS1_NUITMVS1_26650              292  QADNSNCVACHNAD......WTASVHGN..EDQMAALAQFSPSISSASM.DANGTVTVA
S_sp._MR-4_Shewmr4_2510                             300  QLDNSNCIACHNSN......WTAELHTGKTAEKKAVMAQLGMSAT.LAGQ...EDNTAV
S_sp._MR-4_Shewmr4_2508                             292  QADNSNCVACHNAD......WTASVHGN..EGQTAALAQFSPSISSASM.DANGTVTVA
S_putrefaciens_strain_FDAARGOS_681_FOB89_16330      303  QLDNSNCIACHNSA......WTAELHTVKTANKKVLINKYGIETSSIVNP...ETKAAT
S_fidelis_ATCC-BAA-318_L884_L884_RS0114715          306  QADNSNCIACHNAN......WTEELHTAGFADTSALINQYGINAASIIDA...TTQAAT
S_fidelis_ATCC-BAA-318_L884_L884_RS0114740          285  QVDNSNCVACHNPD......WTMSAHSQ..ADTDAVLAQFNAEVVSAEL.NGD.AVNFE
S_piezotolerans_WP3_SWP_RS14705                     305  QADNSNCVACHNAS......WTEELHTSNFGSTKALIDTYGINVTSTIDS...TTTAAT
S_piezotolerans_WP3_SWP_RS14685                     292  QQDNSNCVACHNSD......WTMSAHSQ..ADTNAVLGQFNVEIVSASL.TGT.TVDLA
S_schlegeliana_strain_JCM_11561_JMA39_RS08985       302  QADNSNCIACHNAS......WTEEIHTEGFTQTKALIDTYGMNTTLVVN....EDKTAT
S_schlegeliana_strain_JCM_11561_JMA39_RS08965       296  QVDNSSCVACHNPD......WTINAHSQ..EATDAVLGHFKAEIVSAEL.NGD.TVNFA
S_marisflavi_strain_EP1_CFF01_RS06660               298  QNDNSNCIACHNAS......WTEELHTEGFAQKKAVIDQIGMNATLVAVA.EDN..SAT
S_marisflavi_strain_EP1_CFF01_RS06680               295  QLNNANCVACHNAD......WTASVHND..GGNEQALAQFKPVITKAMR.SGD.TVSFT
S_sp.SUN_WT4__FJQ87_RS07980                         301  QVDNSNCVACHNSQ......WTTEIHTGKVNTKKELINSYGLSSELVAN....SNNTAT
S_sp.MBTL60-112-B2_K5Q73_RS09070                    302  QADNSNCIACHNAS......WTEEIHTEGFVQKKALIDTYGMNTTLVVN....DDSTAT
S_sp.MBTL60-112-B2_K5Q73_RS09050                    292  QTDNSNCVACHNSD......WTKSAHSQ..ADTDAVLGQFNAEVVSAEL.VGD.AVNFS
S_sp.MBTL60_112_B1_K5Q83_RS07460                    302  QADNSNCIACHNAS......WTEEIHTEGFVQKKALIDTYGMNTTLVVN....DDSTAT
S_sp.MBTL60_112_B1_K5Q83_RS07480                    292  QTDNSNCVACHNSD......WTKSAHSQ..ADTDAVLGQFNAEVVSAEL.VGD.AVNFS
S_enrypsychrophilus_strain_YLB-08_FM038_RS07470     306  QNDNSNCIACHNAS......WTEELHTGDFAQTKTLIDSYGMNTSLVVN....QDMTTT
S_enrypsychrophilus_strain_YLB-08_FM038_RS07490     295  QADNSNCVACHNSQ......WTMNVHND..GGDVEALAQFNAEIISASL.SGT.NVTFS
S_sp.YLB_09_FS418_RS09045                           306  QNDNSNCIACHNAS......WTEELHTGDFAQTKTLIDSYGMNTSLVVN....QDMTTT
S_sp.YLB_09_FS418_RS09065                           295  QADNSNCVACHNSQ......WTMNVHND..GGDVEALAQFNAEIISASL.SGT.NVTFS
S_sp._WPAGA9_IGB07_RS05190                          302  QSDNSNCIACHNAS......WTEEIHTGDFVQKKAFIDMYGMTSSLIVN....SDKTAT
S_sp._ARC9_LZ_GUY17_RS06940                         298  QVDNSNCIACHNAS......WTEELHTTSYTQKKAFIDLYGMTATLTANKSSTEDKSAT
S_psychromarinicola_strain_M2_EGC80_RS12750         298  QVDNSNCVACHNAN......WTEELHTTAYTQKKAFIDLYGMTATLTANKTSPDDKSAT
S_sp.Actino-trap-3_CXF80_RS00760                    298  QVDNSNCVACHNAN......WTEELHTTAYTQKKAFIDLYGMTATLTANKASPDDKSAT
S_livingstonensis_strain_LMG_19866_EGC82_RS08000    298  QVDNSNCIACHNAS......WTEELHTTSYTQKKAFIDLYGMTATLTANKSSAEDKSAT
Ferrimonas_lipolytica_strain_S7_HER31_RS03430       303  EQSNSTCSACHTPS......QIEEYHIGDYLNADKVADALATTMTLTAS....VDGAVT
S_sp.ISTPL2_CCLCJOKE_1_HUB64_RS04610                297  QLDNSNCIACHNSD......WTAELHTGKTADKKAVIAQLGMS.AALAGQ...ADDTAV
S_sp.ISTPL2_CCLCJOKE_1_HUB64_RS04620                291  QTDNSNCVACHNAD......WTASVHSD..EAKTAALMQFSPSITSASM.DANGTVTVA
S_japonica_strain_KCTC_22435_SJ2017_RS07855         302  QSDNSNCIACHNAS......WTEEIHTGDFVQKKAFIDMYGMTSSLIVN....SDKTAT
S_sp.8A_M2897_RS07845                               297  QLDNSNCIACHNST......WTAELHTAKTADKKAVIAQLGMS.ATLAAQ...ADNTAV
S_sp.8A_M2897_RS07855                               292  QADNSNCVACHNAD......WTASVHGN..EDQMAALAQFSPSISSASM.DANGTVTVA
S_woodyi_ATCC_51908_Swoo_3125                       307  QDDNSNCIACHNAS......WTEELHTDGFAQKKAFVGQYGMDASLSAVA.TDTGSDTT
Ferrimonas_balerica_DSM_9799_Fbal_1357              303  EQDNSSCAACHNPT......QIESIHMKSHQDEATVVEQFGMTATSVVN....ADNSVT
Ferrimonas_balerica_DSM_9799_Fbal_1362              300  QADNSNCVACHNPD......WTRSAHLQ..TAKADALGQFSATIESITFDPAAASLSIV
S_sediminis_HAW-EB3_Ssed_1525                       307  QADNSNCVACHNAS......WTEELHTEDALQKKAVIDQIGMNATLTAVQ.DDK..SAN
S_sediminis_HAW-EB3_Ssed_1528                       300  QTDNSNCVACHNSD......WTANVHND..GGDDEALGQFNANIVSASL.TGN.TVTFN
S_sp.KX20019_JK628_RS15030                          305  QADNSNCVACHNAS......WTEELHTGSFGATKALIDTYGMNTTLVVN....QNLTAT
Ferrimonas_sp.SCSIO_43195_J8Z22_RS15465             304  QADSSRCASCHTAEGTGVVKGTIDAHLGEWNDAAEVISQWGSDVEMAYFA...DTDTTT
```

```
S_oneidensis_MR-1_mtrC                              350  ISVQVVDANGTAVDLKTILPKVQRLEIITNVGPNNATLGYS.......GKDSIFAIKNG
S_oneidensis_MR-1_mtrF                              342  VSLTNPTTGTAYADSADKLKFISDLRIYANWGTS..........FDYSSRSARSIRLP
S_sp._LZH-2_JM642_12750                             346  LTVSILDKDGNAIDAASVQNKIKRLETITNVGPNFPIMGYNKSPGSGTAKVAKDLVKDG
S_sp._LZH-2_JM642_12740                             342  VTLTNPSTGTAYVDSADKLKFISDLRVYANWGTS..........FDYSTRSARSIRLP
S_xiamenensis_NUITM-VS1_NUITMVS1_26680              346  LTVSILDKDGNAIDAASVQNKIKRLETITNVGPNFPIMGYNKSPGSGTAKVAKDLVKDG
S_xiamenensis_NUITM-VS1_NUITMVS1_26650              342  VTLTNPSTGTAYLDSADKLRFISDLRVYANWGTS..........FDYSTRSARSIRLP
S_sp._MR-4_Shewmr4_2510                             349  LTVTVFDKDGNAIDAASVQDKIKRLETITNVGPNFPIMGYNPSPVTGYKKVAKDLVKDG
S_sp._MR-4_Shewmr4_2508                             342  VTLSNPSTGTVYSDSADKLKFISDLRVYANWGTS..........FDYSTRSARSIRLP
S_putrefaciens_strain_FDAARGOS_681_FOB89_16330      353  ISIQVTDSKGAPVDITALLPQIQRVEIITNVGPNNITLSYFT.......KDSVIAVKNG
S_fidelis_ATCC-BAA-318_L884_L884_RS0114715          356  ISIQVVDSNGEQVDIDTLLPKIQRFEVITNVGPNNVTLGY......SGKDSVNAIMNG
S_fidelis_ATCC-BAA-318_L884_L884_RS0114740          334  IRLSNPATNEVYSDSADKLDFINDLRVYANFGIS..........VDYTTRSAKSIKLQ
S_piezotolerans_WP3_SWP_RS14705                     355  ISIQVVDSAGAEVDINTILPMVQRFEVVTNVGPNNVTLGY......GGKDSINAIKNG
S_piezotolerans_WP3_SWP_RS14685                     341  VRLSNPATNEIYTDSADKLDFVNDLRIYANWGVS..........VDYTTRSAKSIKLQ
S_schlegeliana_strain_JCM_11561_JMA39_RS08985       351  VTVSLIDANGEAVNATTLLPKLQRVEATTNVGPNNVQLGY......YGKDSLNLVLNG
S_schlegeliana_strain_JCM_11561_JMA39_RS08965       345  VRLSNPATNEVYSSSADKLNFINDLRVYANWGIS..........VDYTTRSAKSIKLQ
S_marisflavi_strain_EP1_CFF01_RS06660               348  LTIAFTDANGNALDVNEILPQIDHLESITNVGPNYPIMGYNINPDNGEHKVAIDLIKNN
S_marisflavi_strain_EP1_CFF01_RS06680               344  LALENPSSGERYTSSADQLDFISDLRVYANWGTS..........FDYATRSARSIRLH
S_sp.SUN_WT4__FJQ87_RS07980                         350  LTVTITDKAGTAVDAASLLPKIKQLETITNVGTQYAIMGYNPAPETGFKKVAQDLVKNG
S_sp.MBTL60-112-B2_K5Q73_RS09070                    351  ITVALIDANGEAVNATTLLPKLQRVEATTNVGPNNVQLGY......YGKDSLNLVLNG
S_sp.MBTL60-112-B2_K5Q73_RS09050                    341  IRLSNPATNEIYSDSADQLNFINDLRVYANFGLS..........VDYTTRSAKSIKLQ
S_sp.MBTL60_112_B1_K5Q83_RS07460                    351  ITVALIDANGEAVNATTLLPKLQRVEATTNVGPNNVQLGY......YGKDSLNLVLNG
S_sp.MBTL60_112_B1_K5Q83_RS07480                    341  IRLSNPATNEIYSDSADQLNFINDLRVYANFGLS..........VDYTTRSAKSIKLQ
S_enrypsychrophilus_strain_YLB-08_FM038_RS07470     355  VTVSFVDASGEAVNASSLLPQIQRVEATTNVGPNNVKLGY......YGKDSLNLVLNG
S_enrypsychrophilus_strain_YLB-08_FM038_RS07490     344  IKLSNPTSGEVYADSADKLSFVDDLRIVANWGTS..........FDYATRSAKSIKLQ
S_sp.YLB_09_FS418_RS09045                           355  VTVSFVDASGEAVNASSLLPQIQRVEATTNVGPNNVKLGY......YGKDSLNLVLNG
S_sp.YLB_09_FS418_RS09065                           344  IKLSNPTSGEVYADSADKLSFVDDLRIVANWGTS..........FDYATRSAKSIKLQ
S_sp._WPAGA9_IGB07_RS05190                          351  LSVTITDAAGTAIDASTLVSKIQRIETITNVGPNFPIMGYNASPGNGQAKVAYDLVRNG
S_sp._ARC9_LZ_GUY17_RS06940                         351  LSVTILDANGTAIDAASLVSKIQRIETITNVGPNFPIMGYNPSPGSGLAKISKDFIKAG
S_psychromarinicola_strain_M2_EGC80_RS12750         351  LSVTILDANGTAIDASSLVSKIQRLETNTNVGPNFPIMGYKASPGSGLAKISKDFITAG
S_sp.Actino-trap-3_CXF80_RS00760                    351  LSVTILDANGTAIDATSLVSKIQRVETITNVGPNFPIMGYKASPGSGLAKISKDFITAG
S_livingstonensis_strain_LMG_19866_EGC82_RS08000    351  LSVTILDANGTAIDATSLVSKIQRVETITNVGPNFPIMGYNPSPGSGLAKISKDLIKAG
Ferrimonas_lipolytica_strain_S7_HER31_RS03430       352  ATIGLVDANGTAVDAANYADSLDFLEVIGNINPQATQLNYG........EKAKAVLRG
S_sp.ISTPL2_CCLCJOKE_1_HUB64_RS04610                346  LTVSILDKDGNAIDAATVQDKIKRLESVTNVGPNFPIMGYNPSPGSGKAKIAKDLVKDG
S_sp.ISTPL2_CCLCJOKE_1_HUB64_RS04620                341  VKLMNPATGTVYSESADKLKFINDLRVYVNWGTS..........FDYATRSARSIKLP
S_japonica_strain_KCTC_22435_SJ2017_RS07855         351  LSVTITDAAGTAIDASTLVSKIQRIETITNVGPNFPIMGYNASPGNGQAKVAYDLVKNG
S_sp.8A_M2897_RS07845                               346  LTVSILDKDGNAIDAASVQNKIKRLETITNVGPNFPIMGYNKSPGSGTAKVAKDLVKDG
S_sp.8A_M2897_RS07855                               342  VTLINPSTGTAYLDSADKLRFISDLRVYANWGTS..........FDYSTRSARSIRLP
S_woodyi_ATCC_51908_Swoo_3125                       359  LSVTITNAEGIALDASALVANIQQLETITNVGPNFPIMGYNPNP.AGLKKVAIDFVKAG
Ferrimonas_balerica_DSM_9799_Fbal_1357              352  ISVNLTE.NGQPIDAGSVVNSLDMVEYVSNVGPKFPVLGYY........SKDSKAFHG
Ferrimonas_balerica_DSM_9799_Fbal_1362              351  VNLSNPVTGEALS.SPDQLPYVNDLRLYANWGTS..........FDYSTTSAPNIRLE
S_sediminis_HAW-EB3_Ssed_1525                       357  LTISFTDSAGNVLDVTNLLPKIKRVESITNVGPNFPIMGYNPSPGNGEHKVALDLVQSG
S_sediminis_HAW-EB3_Ssed_1528                       349  ITLTNPVTNTVYADSADKLDFIDDLRVYANWGTS..........FDYAP.SAKSLKLQ
S_sp.KX20019_JK628_RS15030                          354  VTVSLVDANGQAVNASTLVPQIQRVEATTTVGPNNVQGGY......NGHDSLNLVLNG
Ferrimonas_sp.SCSIO_43195_J8Z22_RS15465             360  VTVSITDANGQKLAADQVLPQIKRLEVLTNLGPNYPVLSY......YTGSHLDAVSNG
```

S_oneidensis_MR-1_mtrC	402	ALDPKATINDAGKLVYTTTKDLKLGQ....NGADSDTAFSFVGWSMCSSEGKFVDCADP
S_oneidensis_MR-1_mtrF	390	ESTPIAGSN.GTYSYNISGLTVPAGT.....ESDRG.GLAIQG.RVCAKDSVLVDCSTE
S_sp._LZH-2_JM642_12750	405	ALQADVTIADG.KLVFTTP.ALPFGA....G..DTDTAFTFIGLEMCNTGTTLTACTAN
S_sp._LZH-2_JM642_12740	390	DSTPVSGSN.GTYVYTISGLTVPAGT.....EADHG.GLAIQG.RVCAKDKVLVDCSTE
S_xiamenensis_NUITM-VS1_NUITMVS1_26680	405	ALQADVTIADG.KLVFTTP.ALPFGA....G..DTDTAFTFIGLEMCNTGTTLTACTAN
S_xiamenensis_NUITM-VS1_NUITMVS1_26650	390	ESTPVSGSN.GTYVYTISGLTVPAGT.....EADHG.GLAIQG.RVCAKDKVLVDCSTE
S_sp._MR-4_Shewmr4_2510	408	ALQDGVTLVDG.KLVFTTP.ALPFG......AGDTDTAFTFIGLEMCNTGTTLTACTAD
S_sp._MR-4_Shewmr4_2508	390	ESTPVSGNN.GTYTYTISGLTVPAGT.....EADHG.GLAIQG.RVCAKDKVLVDCSTE
S_putrefaciens_strain_FDAARGOS_681_FOB89_16330	405	VLDSNASIVDG.KLLYTTTKPLPFGA....AKTDTDTSVTFVNWAMCSLNGQFVTCAEP
S_fidelis_ATCC-BAA-318_L884_L884_RS0114715	408	VVDAKAKIEAGKLVYTTTK.DLKLG....AAGEDAETAFTFVGWAMCSENGEFVTCDDP
S_fidelis_ATCC-BAA-318_L884_L884_RS0114740	382	DIEPISGSN.GTYQYQIAGLTLSDDP.....TTDKG.TLALQG.RLCSNGAMLVNCDDT
S_piezotolerans_WP3_SWP_RS14705	407	VVDAKAAIEDGKLVYTTTK.PLNLNT...EVGADDETAFTFAGWAMCSANGEFVKCDDP
S_piezotolerans_WP3_SWP_RS14685	389	EITPISGND.GVFNYQIAGLTVPAEL.....VDDTG.TLAIQG.KLCSDEDMLANCADI
S_schlegeliana_strain_JCM_11561_JMA39_RS08985	403	KLDATAAINEEGNIVYTTK.PLTFGT......DDADTAFTFSGLSMCSENGEFVDCDKV
S_schlegeliana_strain_JCM_11561_JMA39_RS08965	393	DATPISGSN.GVYHYRIAGLTLSADP.....ATDKG.ALALQG.KLCNDNAMLVDCAET
S_marisflavi_strain_EP1_CFF01_RS06660	407	ELSSAVQVVD.GSLAVNTG.KLPF....GESGSDADTAFSFIGLSMCAENGKLVNCGEG
S_marisflavi_strain_EP1_CFF01_RS06680	392	EITPLAGSN.GAFDYQVTGLSVPAGS.....EMDKG.TLAIQG.RICAADGVLGSCDNE
S_sp.SUN_WT4__FJQ87_RS07980	409	ALVAPVALVN.GKLVFTTP.VLPFN....QGAADTNTAFSFIGLGMCNDGTAFVNCADG
S_sp.MBTL60-112-B2_K5Q73_RS09070	403	KLDAAAAINEDGDIVYTTK.ALKFGT......GDADTAFTFSGLSLCSENGEFVNCDTV
S_sp.MBTL60-112-B2_K5Q73_RS09050	389	DTTPISGSN.GVYHYQIAGLTLSAEP.....ASDKG.TLALQG.KLCSTESMLADCADT
S_sp.MBTL60_112_B1_K5Q83_RS07460	403	KLDAAAAINEDGDIVYTTK.ALKFGT......GDADTAFTFSGLSLCSENGEFVNCDTV
S_sp.MBTL60_112_B1_K5Q83_RS07480	389	DTTPISGSN.GVYHYQIAGLTLSAEP.....ASDKG.TLALQG.KLCSTESMLADCADT
S_enrypsychrophilus_strain_YLB-08_FM038_RS07470	407	QLNPAATITEAGAISYTTK.ALTFGA....TGEDADTAFTFTGLSLCSDKGEFVNCDTV
S_enrypsychrophilus_strain_YLB-08_FM038_RS07490	392	DTPPVSGKE.GTYTYEIAGLTIPVGS.....ETDHG.ALAVQG.KICSSDGELGDCSDE
S_sp.YLB_09_FS418_RS09045	407	QLNPAATITEAGAISYTTK.ALTFGA....TGEDADTAFTFTGLSLCSDKGEFVNCDTV
S_sp.YLB_09_FS418_RS09065	392	DTPPVSGKE.GTYTYEIAGLTIPVGS.....ETDHG.ALAVQG.KICSSDGELGDCSDE
S_sp._WPAGA9_IGB07_RS05190	410	ELGENVSVEA.GALQYTLP.ALPFDEYSPAGIQDTDTAFTFVGLEMCNDGTEAVDCGED
S_sp._ARC9_LZ_GUY17_RS06940	410	LLQADVVIES.GNLVYNIA.SLPF......GAGDTDTAFSFIGLEMCNDGIQAIDCVDG
S_psychromarinicola_strain_M2_EGC80_RS12750	410	LLQTDVVIES.GNLVYNIA.SLPF......GAGDTDTAFSFIGLEMCNDGIQAIDCADG
S_sp.Actino-trap-3_CXF80_RS00760	410	LLQTDVVIES.GNLVYNIA.SLPF......GAGDTDTAFSFIGLEMCNDGIQAIDCADG
S_livingstonensis_strain_LMG_19866_EGC82_RS08000	410	LLQADVVIES.GKLVYNIA.SLPF......GSGDTDTAFSFIGLEMCNDGLQAIDCVDS
Ferrimonas_lipolytica_strain_S7_HER31_RS03430	402	..DEIAATIVDGNLVVVFD.DLLAGD......AATESALVIAGLRFCAEDGDFAPCSEE
S_sp.ISTPL2_CCLCJOKE_1_HUB64_RS04610	405	ALQEGVTLVDG.KLIFTTP.ALPFGA....G..DTDTAFTIIGLEVCSTGTSLTACTAD
S_sp.ISTPL2_CCLCJOKE_1_HUB64_RS04620	389	ESTPISGSN.GIYTYTIAGLTVPAGT.....ETDHG.GLAIQG.RICAKDKVLVDCTTE
S_japonica_strain_KCTC_22435_SJ2017_RS07855	410	ELGENVSVEA.GALQYTLP.ALPFDEYSPAGIQDTDTAFTFVGLEMCNDGTEAVDCGED
S_sp.8A_M2897_RS07845	405	ALQADVTIADG.KLVFTTP.ALPFGA....G..DTDTAFTFIGLEMCNTGTTLTACTAN
S_sp.8A_M2897_RS07855	390	DSTPVSGSN.GTYVYTISGLTVPAGT.....EADHG.GLAIQG.RVCAKDKVLVDCSTE
S_woodyi_ATCC_51908_Swoo_3125	417	ILDANVEIVD.GKFVTVIK.NLPY....GTG..DTDTAFSFVGLALCNDGTDLVACGEG
Ferrimonas_balerica_DSM_9799_Fbal_1357	401	..EEIAAAIEGGSVVYTTK.ALPFAE......GDADTALTMVGVRVCAENGVLAECSDN
Ferrimonas_balerica_DSM_9799_Fbal_1362	398	SLTPDTVLGPGQYQYTLPGLTIPPGT.....EADQGGAVAMQG.RICRSGDTLVPCEDA
S_sediminis_HAW-EB3_Ssed_1525	416	ELANNAEIAD.GNLVINTG.TLPF....GAAGTDTDTAFSFIGLEVCSDGAELINCAEG
S_sediminis_HAW-EB3_Ssed_1528	396	DSSPISGSN.GIYTYAISGLTIPAGT.....ESDRG.VLAIQG.KLCSSQGELGDCEED
S_sp.KX20019_JK628_RS15030	406	ELNAAATITEDGNISYTTA.VLPFPAD.VTAGADADTAFTFTGLSLCSEDGAFVNCDTV
Ferrimonas_sp.SCSIO_43195_J8Z22_RS15465	412	ELANNTTIVDGNFVLPITG..LPYGA....EGTDADTAFSFVGLAACSKGNAIVACADV

S_oneidensis_MR-1_mtrC	457	AFDGV...................DVTKYTGMKADLAFATLSGKAPSTRHVDSVNMTAC
S_oneidensis_MR-1_mtrF	441	L.........................AEVLVIKSSHSYFNMSALTTTGRRE.VISNAKC
S_sp._LZH-2_JM642_12750	456	..........................STTTSMKAELAFGTKSGKAPSTRHVNSVNFSTC
S_sp._LZH-2_JM642_12740	441	Q.........................AEVLVLKSSHSYFNMSALTSTGRRE.IISNANC
S_xiamenensis_NUITM-VS1_NUITMVS1_26680	456	..........................STTTSMKAELAFGTKSGKAPSTRHVNSVNFSTC
S_xiamenensis_NUITM-VS1_NUITMVS1_26650	441	Q.........................AEVLVIKSSHSYFNMSALTSTGRRE.IISNANC
S_sp._MR-4_Shewmr4_2510	459	STT..........................TSMKADLVFGTKSSNAPSMRHVNSVNFSAC
S_sp._MR-4_Shewmr4_2508	441	L.........................AEVLVIKASHSYFDMSALSTTGRRE.VISNANC
S_putrefaciens_strain_FDAARGOS_681_FOB89_16330	459	TFDGA...................DVSKYTSMKADIAFATLSGEKPSTRHVDSVNFSTC
S_fidelis_ATCC-BAA-318_L884_L884_RS0114715	462	SFDGK...................DTSKYTAMKSDLAFATLSGEAPSMRHVDSVNFTSC
S_fidelis_ATCC-BAA-318_L884_L884_RS0114740	433	E.........................TTTT.IDSSYQFFSATEVTDQGRRM.VVSNETC
S_piezotolerans_WP3_SWP_RS14705	462	DFDGV...................DTSKYTGMKADLAFATLSGEAASMRHVDSVNFSSC
S_piezotolerans_WP3_SWP_RS14685	440	D.........................NTTN.LKSSHQFFSASAISDVGRRV.VVTNETC
S_schlegeliana_strain_JCM_11561_JMA39_RS08985	455	AVEGNLDE............KGYLLDAFYTGMKADLAFATFSGEAPSMRHVDSVNFDTC
S_schlegeliana_strain_JCM_11561_JMA39_RS08965	444	D.........................STTI.IKSSHQFFSATAVTDLGRRV.VVTNETC
S_marisflavi_strain_EP1_CFF01_RS06660	460	..........................VPTVTMKADLAHGTLSGEAPSYRHTDSVNFASC
S_marisflavi_strain_EP1_CFF01_RS06680	443	A.........................NSQIVIKSSHSFFSSEALSDSGRRV.VVTNDTC
S_sp.SUN_WT4__FJQ87_RS07980	462	..........................VDFTGMKADLAFAAQGGKVT.TRHIDSVAIDTC
S_sp.MBTL60-112-B2_K5Q73_RS09070	455	VVEENLDTDWQG........NKSLNSAYYTGMKADIAFATFSGEAVSKRHVDSVNFDTC
S_sp.MBTL60-112-B2_K5Q73_RS09050	440	D.........................TTTT.IKSSHQFFSATAITEQGRRV.VVTNETC
S_sp.MBTL60_112_B1_K5Q83_RS07460	455	VVEENLDTDWQG........NKSLNSAYYTGMKADIAFATFSGEAVSKRHVDSVNFDTC
S_sp.MBTL60_112_B1_K5Q83_RS07480	440	D.........................TTTT.IKSSHQFFSATAITEQGRRV.VVTNETC
S_enrypsychrophilus_strain_YLB-08_FM038_RS07470	461	AVDGN..LDEDG..........YLVDAYYTGMKADLTFATLSGEAPSMRHVDSVNFNTC
S_enrypsychrophilus_strain_YLB-08_FM038_RS07490	443	S.........................FSTVNIKSSHQFFNQSALSTEGRRV.VVTNETC
S_sp.YLB_09_FS418_RS09045	461	AVDGN..LDEDG..........YLVDAYYTGMKADLTFATLSGEAPSMRHVDSVNFNTC
S_sp.YLB_09_FS418_RS09065	443	S.........................FSTVNIKSSHQFFNQSALSTEGRRV.VVTNETC
S_sp._WPAGA9_IGB07_RS05190	467	..........................IATTSMKADLVFGTFSGEAPSMRHTDSVAFNTC
S_sp._ARC9_LZ_GUY17_RS06940	461	..........................VATTSMKAEFTVGTFSGNAPSYRHTDSVNFGSC
S_psychromarinicola_strain_M2_EGC80_RS12750	461	..........................VATTSMKAELTVGTFSGNAPSYRHIDSVNFGSC
S_sp.Actino-trap-3_CXF80_RS00760	461	..........................VATTSMKAEFTVGTFSGNAPSYRHIDSVNFGSC
S_livingstonensis_strain_LMG_19866_EGC82_RS08000	461	..........................VATTSMKAEFTVGTFSGNAPSYRHTDSVNFGSC
Ferrimonas_lipolytica_strain_S7_HER31_RS03430	452	..........................ADYVSIDSAAGTVAIEGVTINNRHYDSIDSSTC
S_sp.ISTPL2_CCLCJOKE_1_HUB64_RS04610	456	..........................SATTSMKAELAFGTKSGKAPSMRHVNSVEFGTC
S_sp.ISTPL2_CCLCJOKE_1_HUB64_RS04620	440	L.........................AEVLVIKSSHSYFDMAALSNEGRRS.VASNASC
S_japonica_strain_KCTC_22435_SJ2017_RS07855	467	..........................IATTSMKADLVFGTFSGEAPSMRHTDSVAFNTC
S_sp.8A_M2897_RS07845	456	..........................STTTSMKAELAFGTKSGKAPSTRHVNSVNFSTC
S_sp.8A_M2897_RS07855	441	Q.........................AEVLVIKSSHSYFNMSALTSTGRRE.VISNANC
S_woodyi_ATCC_51908_Swoo_3125	468	..........................VESTGMKAELAHGTLSGEAPSVRHTDTVEFTAC
Ferrimonas_balerica_DSM_9799_Fbal_1357	451	..........................ADKIALDAYTSFVSKSGAALSERHN.SLDNASC
Ferrimonas_balerica_DSM_9799_Fbal_1362	451	N.........................AQTTPIASFTEFFTAG..TGTARRD.VVSNETC
S_sediminis_HAW-EB3_Ssed_1525	469	..........................VATTSMKAELAHGTLSGEAPSTRHIDSVNFATC
S_sediminis_HAW-EB3_Ssed_1528	447	A.........................FSTVNLKSSHQFFSAAELTDTGRRV.VVTNDTC
S_sp.KX20019_JK628_RS15030	463	YKEGNYVLDDKGEVKLDSKGNPYLIDALYTGMKADLTFATLSGDAASMRHVDSVNFSAC
Ferrimonas_sp.SCSIO_43195_J8Z22_RS15465	465	ADPDN....................TDNYTGMKANMAFVTKSGFAPSKRHTDSLEFSKC

S_oneidensis_MR-1_mtrC	497	ANCHTAEFE..IHKGK..QHAGFVMTEQLSHTQDANGKAIVGLDACVTCHTPDGTYSF.
S_oneidensis_MR-1_mtrF	474	ASCHGDQQLNIHGAR.....NDLAGQCQLCHNPNMLADATATNPSMTSFDFKQLIHGLH
S_sp._LZH-2_JM642_12750	489	QGCHSDTFE..IHKG...HHAGFVMSEQVSHAKDANGKPIVGVDACVACHTPDGTYANG
S_sp._LZH-2_JM642_12740	474	ANCHGDQQLNIHGAR.....NDLAGQCQLCHNPNMQADATAANPSITSFDFKQLIHGIH
S_xiamenensis_NUITM-VS1_NUITMVS1_26680	489	QGCHSDTFE..IHKG...HHAGFVMSEQVSHAKDANGKPIVGVDACVACHTPDGTYANG
S_xiamenensis_NUITM-VS1_NUITMVS1_26650	474	ASCHGDQQLNIHGAR.....NDLAGQCQLCHNPNMQADATAANPSITSFDFKQLIHGIH
S_sp._MR-4_Shewmr4_2510	492	QNCHSETFE..IHKG...YHAGFVMTEQVSHSKDASDKPIVGVDACVTCHTPEGAGYS.
S_sp._MR-4_Shewmr4_2508	474	ASCHGDQQLNIHGAR.....NDLAGQCQLCHNPNMQADATAVNPSITSFDFKQLIHGIH
S_putrefaciens_strain_FDAARGOS_681_FOB89_16330	499	ANCHGTEWESRYHKGK..NSPGFVMSEQLAHSKDAAGKPIVGLDGCATCHTPHGTYASG
S_fidelis_ATCC-BAA-318_L884_L884_RS0114715	502	ANCHSDDFQIH.KGG...HHAGFVMTEQLAHTNDANGEPIIGVDACTTCHTPDGTYAGG
S_fidelis_ATCC-BAA-318_L884_L884_RS0114740	465	GSCHGDQQLNYHGSR.....NDLEQQCQLCHNPNMQADASAINPAASTADYKHLAHTLH
S_piezotolerans_WP3_SWP_RS14705	502	ANCHSDEFQVH.KGS...HHAGFVMSDQLAHTNDADGMPIIGVDACVACHTPDGTYAGG
S_piezotolerans_WP3_SWP_RS14685	472	GSCHGDQELNYHGSR.....NDLEGQCQVCHNRNMQAEAGAANAAASTADYKHLAHTLH
S_schlegeliana_strain_JCM_11561_JMA39_RS08985	502	VSCHGDTFEIH.KGS...HHPGFVMSEQLAQIVDG..KTIVGVDGCVACHTPDGTYAGG
S_schlegeliana_strain_JCM_11561_JMA39_RS08965	476	GSCHGDQQLNYHGSR.....NDLEQQCQLCHNPNMLADASATNPGASTADYKHLAHTLH
S_marisflavi_strain_EP1_CFF01_RS06660	493	KNCHGSEWPLNSHTK...YHTGFVLSEQLGRPNAD.GEMIVGIDGCVTCHTPDGTYASG
S_marisflavi_strain_EP1_CFF01_RS06680	476	GSCHGDQQLNFHGSR.....NDLEGQCQLCHNPNMVAEASE.NPSTTTADFKHLIHAIH
S_sp.SUN_WT4__FJQ87_RS07980	494	INCHGDSFDIH..KG...YHAGFVLSEQLGREIDG..KLMTGIDGCVSCHTPHGTYGSG
S_sp.MBTL60-112-B2_K5Q73_RS09070	506	ISCHGDTFEIH.KGS...HHPGFVMSEQLAQIVDG..KTIVGVDGCVACHTPDGTYANG
S_sp.MBTL60-112-B2_K5Q73_RS09050	472	GSCHGDQQLNYHGSR.....NDLEGQCQLCHNPNMQADATVTNPGASTADYKHLAHTLH
S_sp.MBTL60_112_B1_K5Q83_RS07460	506	ISCHGDTFEIH.KGS...HHPGFVMSEQLAQIVDG..KTIVGVDGCVACHTPDGTYANG
S_sp.MBTL60_112_B1_K5Q83_RS07480	472	GSCHGDQQLNYHGSR.....NDLEGQCQLCHNPNMQADATVTNPGASTADYKHLAHTLH
S_enrypsychrophilus_strain_YLB-08_FM038_RS07470	508	INCHGDSFEVH.KGT...HHPGFVMSEQLAQIVNG..ETIVGVDGCVACHTPDGTYAGG
S_enrypsychrophilus_strain_YLB-08_FM038_RS07490	476	GSCHGDQALNFHGSR.....NDLEQQCQLCHNNNMMADASAANPSIATADFKHLIHGLH
S_sp.YLB_09_FS418_RS09045	508	INCHGDSFEVH.KGT...HHPGFVMSEQLAQIVNG..ETIVGVDGCVACHTPDGTYAGG
S_sp.YLB_09_FS418_RS09065	476	GSCHGDQALNFHGSR.....NDLEQQCQLCHNNNMMADASAANPSIATADFKHLIHGLH
S_sp._WPAGA9_IGB07_RS05190	500	ENCHGETFEIH..KG...YHAGFVMTEQLGRENDE.GEVVVGVDACVTCHTPDGTYASG
S_sp._ARC9_LZ_GUY17_RS06940	494	DNCHGDTFELH..KG...HHAGFVMSEQLARELN..GEVTIGLDGCVACHTPDGTYASG
S_psychromarinicola_strain_M2_EGC80_RS12750	494	DNCHGDTFELH..KG...HHAGFVMTEQLARELN..GEVTVGLDACVVCHTPDGTYASG
S_sp.Actino-trap-3_CXF80_RS00760	494	DNCHGDTFELH..KG...HHAGFVMTEQLARELN..GELTVGLDACVVCHTPDGTYASG
S_livingstonensis_strain_LMG_19866_EGC82_RS08000	494	DNCHGDTFELH..KG...HHAGFVMSEQLARELN..GEVTIGLDGCVACHTPDGTYASG
Ferrimonas_lipolytica_strain_S7_HER31_RS03430	485	FNCHGDNFQVHNSGVEDSHHSGYTFND............NMQVGDCAACHNTYGTYAYG
S_sp.ISTPL2_CCLCJOKE_1_HUB64_RS04610	489	QGCHGDSFE..IHKG...HHAGFVMTEQLSHAKDASGKPIVGVDACVACHTPDGTYANG
S_sp.ISTPL2_CCLCJOKE_1_HUB64_RS04620	473	GNCHGDQKLNIHGSR.....NDLDGQCQLCHNANMLADATAANPSITSFDFKQLIHGIH
S_japonica_strain_KCTC_22435_SJ2017_RS07855	500	ENCHGETFEIH..KG...YHAGFVMTEQLGRENDE.GEVVVGVDACVTCHTPDGTYASG
S_sp.8A_M2897_RS07845	489	QGCHSDTFE..IHKG...HHAGFVMSEQVSHAKDANGKPIVGVDACVACHTPDGTYANG
S_sp.8A_M2897_RS07855	474	ASCHGDQQLNIHGAR.....NDLAGQCQLCHNPNMQADATAANPSITSFDFKQLIHGIH
S_woodyi_ATCC_51908_Swoo_3125	501	ESCHGTEWGIH.QGS...RHPGFVMTEQLGRVNEA.GEMIVGIDGCASCHTPDGTYASG
Ferrimonas_balerica_DSM_9799_Fbal_1357	483	LGCHGETFQLHNG....SHHAGFVLND...........NIKVGDCAACHTPEGTYAP.
Ferrimonas_balerica_DSM_9799_Fbal_1362	482	GSCHGDQQLNFHGAR.....NDLSQQCQLCHNGQMVATAGADNVSATNANYSHMIHAIH
S_sediminis_HAW-EB3_Ssed_1525	502	ESCHGETFEL..HKG...YHAGFIMTEQLGR.EVN.GQMTVGVDGCVACHTPDGTYMSG
S_sediminis_HAW-EB3_Ssed_1528	480	GSCHGVQALNFHGAR.....NDLQGQCQLCHNPNMMADATASNPSVSTADYKHLIHSLH
S_sp.KX20019_JK628_RS15030	522	STCHSAEFQVH.KGT...HHPGFVMSEQLSHATDANDQPIIGVDGCVACHTPDGTYAGG
Ferrimonas_sp.SCSIO_43195_J8Z22_RS15465	504	EGCHNDNWQIH..KG...HHSGFVMTEQVAHDEEG...KVIGLDGCVTCHTPDGTYAP.

S_oneidensis_MR-1_mtrC	551	ANRGALELKLHKKHV.EDAYGLIGGNCASCHSDFNLESFKKKGALNTAAAA.....DKT
S_oneidensis_MR-1_mtrF	528	SSQFAGFEDLNYPGN........IGNCAQCHINDSTGISTVALPLNAAVQP...LALNN
S_sp._LZH-2_JM642_12750	543	ANKGGFEMKLHVVHG.EQG...VIKECTQCHNDFNLDAFKVKGALATS..........A
S_sp._LZH-2_JM642_12740	528	TSQFTGFADLNYPGK........IGNCAQCHIKDAAGVSTVALPLNTAVQP...LALNN
S_xiamenensis_NUITM-VS1_NUITMVS1_26680	543	ANKGGFEMKLHVVHG.EQG...VIKECTQCHNDFNLDAFKVKGALATS..........A
S_xiamenensis_NUITM-VS1_NUITMVS1_26650	528	TSQFAGFADLNYPGK........IGNCAQCHIKDAAGVSTVALPLNTAVQP...LPLNN
S_sp._MR-4_Shewmr4_2510	545	GTGMALEIRAHKTHA.EKANGLIGDNCTQCHNDFNLDAFKVKGAMEFN..........K
S_sp._MR-4_Shewmr4_2508	528	TSQFAGFEDLNYPGK........IGNCAQCHIKDAAGVSTVALPLNAAVQP...LALNN
S_putrefaciens_strain_FDAARGOS_681_FOB89_16330	556	ANKGALEMKLHVVHG.KQG...VIKECTQCHNDFNLDSFKVKGALATA..........A
S_fidelis_ATCC-BAA-318_L884_L884_RS0114715	557	ANQGALEMKLHKVHSQGDYAVIPGLNCDQCHNDFDRDAFKKKGAIATDGGQ........
S_fidelis_ATCC-BAA-318_L884_L884_RS0114740	519	AGSRESYPDINYPGN........IGNCAQCHTTDEAGILTAALPLSAAVQP...LAFND
S_piezotolerans_WP3_SWP_RS14705	557	SNMGALEMKLHKTHSQGDYAVIPGMNCDQCHSDFNRDAFKKKGALATDVQDPYTP.TQK
S_piezotolerans_WP3_SWP_RS14685	526	AGSRENYPDINYPAN........IGNCAQCHTEDASGVLTAALPLNSAVQP...LAFDD
S_schlegeliana_strain_JCM_11561_JMA39_RS08985	555	ANMGALELKLHKVHSSGDYAAIAGMNCDQCHNDLNLDAFKQKGALAT..........AG
S_schlegeliana_strain_JCM_11561_JMA39_RS08965	530	AGSRESYPNINYPGK........IGNCAQCHTTDAAGVLTAALPLNKAVQP...LAFSD
S_marisflavi_strain_EP1_CFF01_RS06660	548	ANKGALEMKLHVVH..GEQGIIK..DCAQCHNDFNLDAFKAKGALAT..........AA
S_marisflavi_strain_EP1_CFF01_RS06680	529	SGQRAGYEAINYPGD........IGNCAQCHSDND.GVLSPALPLDPGVKP...LAISD
S_sp.SUN_WT4__FJQ87_RS07980	546	ANKGALELKLHKTHS.AFYGLIGG.NCSQCHNDFNLDAFKAKGALAT..........DG
S_sp.MBTL60-112-B2_K5Q73_RS09070	559	ANMGALEMKLHKVHSSGDHAVITGMTCNQCHNDLNLDAFKKKSAFATKVDEVSTGGEAV
S_sp.MBTL60-112-B2_K5Q73_RS09050	526	AGSRESYPNINYPGN........IGNCAQCHTTDESGILTAALPLNSAVQP...LAFKD
S_sp.MBTL60_112_B1_K5Q83_RS07460	559	ANMGALEMKLHKVHSSGDHAVITGMTCNQCHNDLNLDAFKKKSAFATKVDEVSTGGEAV
S_sp.MBTL60_112_B1_K5Q83_RS07480	526	AGSRESYPNINYPGN........IGNCAQCHTTDESGILTAALPLNSAVQP...LAFKD
S_enrypsychrophilus_strain_YLB-08_FM038_RS07470	561	ANKGALEMKLHKTHSQGDYAVIPGMNCDQCHNDFNFDAFKKKGALAT..........AS
S_enrypsychrophilus_strain_YLB-08_FM038_RS07490	530	SSQREGYEDLTYPGQ........IGNCAQCHTNSDAGVLTAALPLNSAVQP...LSLDD
S_sp.YLB_09_FS418_RS09045	561	ANKGALEMKLHKTHSQGDYAVIPGMNCDQCHNDFNFDAFKKKGALAT..........AS
S_sp.YLB_09_FS418_RS09065	530	SSQREGYEDLTYPGQ........IGNCAQCHTNSDAGVLTAALPLNSAVQP...LSLDD
S_sp._WPAGA9_IGB07_RS05190	553	GNQGALEMKLHSVH..GEEGIIS..DCAQCHNDFNLDAFKQKGALAT..........SA
S_sp._ARC9_LZ_GUY17_RS06940	546	ANQGAIEMKLHSVH..SEQGIIK..DCTQCHNDFNLDTFKVKGALAT..........SQ
S_psychromarinicola_strain_M2_EGC80_RS12750	546	ANQGAIEMKLHSVH..GEQGIIK..DCTQCHNDFNLDAFRVKGALAT..........SA
S_sp.Actino-trap-3_CXF80_RS00760	546	ANQGAIEMKLHSVH..GEQGIIK..DCTQCHNDFNLDAFRVKGALAT..........SA
S_livingstonensis_strain_LMG_19866_EGC82_RS08000	546	ANKGAIEMKLHSVH..GEQGIIK..DCTQCHNDFNLDAFKVKGALAT..........SA
Ferrimonas_lipolytica_strain_S7_HER31_RS03430	532	ANQGALEMKLHSAHSSDVADPIVAGDCTKCHTGFNTDSFEDKTAMATGS....FYSAET
S_sp.ISTPL2_CCLCJOKE_1_HUB64_RS04610	543	ANKGALEMKLHVVHG.KQG...VIKECTQCHNDFNLDAFKVKGALATT..........A
S_sp.ISTPL2_CCLCJOKE_1_HUB64_RS04620	527	TSQFTGFENLNYPGK........IGNCAQCHVNDTAGISTVALPLNSAVQP...LALNN
S_japonica_strain_KCTC_22435_SJ2017_RS07855	553	GNQGALEMKLHSVH..GEEGIIS..DCAQCHNDFNLDAFKQKGALAT..........SA
S_sp.8A_M2897_RS07845	543	ANKGGFEMKLHVVHG.EQG...VIKECTQCHNDFNLDAFKVKGALATS..........A
S_sp.8A_M2897_RS07855	528	TSQFAGFADLNYPGK........IGNCAQCHIKDAAGVSTVALPLNTAVQP...LALNN
S_woodyi_ATCC_51908_Swoo_3125	555	ANLGSIEMKLHKTHSQGDYGAIPGMNCSQCHSDFKQEAFAKKGALAT..........DG
Ferrimonas_balerica_DSM_9799_Fbal_1357	525	TNMGAIELKLHKVHG....EQWIVADCAQCHTDFEVGSFNVKGALNTG.....AAEDGT
Ferrimonas_balerica_DSM_9799_Fbal_1362	536	TGQRAGYEELVYPAP........VGNCRQCHISGDEGDS.FALPLLTSLPP...MALDD
S_sediminis_HAW-EB3_Ssed_1525	554	GNKGAFEMKLHVVH..GEQGIIK..DCAQCHNDFNLDAFKVKGALAT..........PA
S_sediminis_HAW-EB3_Ssed_1528	534	SAQREGYEGLNYPGQ........IGNCAQCHASDDTGLLSAALPLNLGVKP...LAIDN
S_sp.KX20019_JK628_RS15030	577	TNKGALEMKLHVVH..GAQQVIN..DCTQCHTGLNLEAFKVKSAFATKVVDAYTP.SQK
Ferrimonas_sp.SCSIO_43195_J8Z22_RS15465	554	TKNGAWEQKLHVVHG..DKNIIN..DCTQCHTSFNLNAFAKKGAINTG.....VTVDGN

```
S_oneidensis_MR-1_mtrC                           604  GLYSTPITATCTTCHTVGSQYM..VHTKETLESFG....AVVDGTKDDATSAAQSETCF
S_oneidensis_MR-1_mtrF                           576  GTFTSPIAAVCSNCHSSD.......ATQNHMRQQG.....AVFAGTKADATAGTETCA
S_sp._LZH-2_JM642_12750                          588  GKYTTPITATCTSCHAP.......ESIGHGLENMG....AIVNGDYTQANQAAQSETCF
S_sp._LZH-2_JM642_12740                          576  GTFTSPIAAVCSNCHSSD.......ATHNHMKQQG.....AVFAGSKADATAGSETCA
S_xiamenensis_NUITM-VS1_NUITMVS1_26680           588  GKYTTPITATCTSCHAP.......ESIGHGLENMG....AIVNGDYTQANQAAQSETCF
S_xiamenensis_NUITM-VS1_NUITMVS1_26650           576  GTFTSPIAAVCSNCHSSD.......ITHNHMKQQG.....AVFAGSKADATAGTETCA
S_sp._MR-4_Shewmr4_2510                          593  GSYTTPITATCTSCHAPES.......IGHGLENMG....AIVNGDYTQANQAAQSETCF
S_sp._MR-4_Shewmr4_2508                          576  GTFTSPIAAVCSNCHSSD.......ATHNHMMQQG.....AVFAGTKADATAGTETCA
S_putrefaciens_strain_FDAARGOS_681_FOB89_16330   601  GKYTTPITATCISCHTP.......ESIGHGLENMG....AIVNGDYTQANQAVQSETCF
S_fidelis_ATCC-BAA-318_L884_L884_RS0114715       608  ..YSTPIAATCVSCHSYNMDS.....FKAHVEGQG....ALVGVSKQEATDAAQLESCF
S_fidelis_ATCC-BAA-318_L884_L884_RS0114740       567  GSFTSATSAICSACHLSD.......SAKAHMTQQG.....GVFNGSEADATAGTESCA
S_piezotolerans_WP3_SWP_RS14705                  615  SLYTTPIAATCASCHSYNMDK.....FKSHVEGQG....ALVNVSKEAANDAAQLETCF
S_piezotolerans_WP3_SWP_RS14685                  574  GSFTSPASAICSACHTSD.......TSKSHMTQQG.....GVFNGTEADATSGTESCA
S_schlegeliana_strain_JCM_11561_JMA39_RS08985    604  GSYTTPIAATCMSCHVYSPESE..AKFAAHAEGQG....ALVNVDKALASDAAQLETCF
S_schlegeliana_strain_JCM_11561_JMA39_RS08965    578  GTFTSATSAICSDCHLSD.......TAISHMTQQG.....GVFKGTEADATAGTESCA
S_marisflavi_strain_EP1_CFF01_RS06660            593  GQYSSPIAATCTSCHTS..DS.....VKAHAEKQG....AVFNDFKETASNA..VETCF
S_marisflavi_strain_EP1_CFF01_RS06680            576  GSYTSPTAAICSDCHSSE.......TNKNHMIQQG.....AVFMGTLSDATAGTESCA
S_sp.SUN_WT4__FJQ87_RS07980                      593  GVYTTPITATCGSCHIIGSEY.....FDAHTQAQLEGFGAVINGDFTAATQAAQSETCL
S_sp.MBTL60-112-B2_K5Q73_RS09070                 618  SLYTTPIAATCMSCHVYSPESE..AKFTAHAEGQG....AKVNVPVAIADDAAQLETCF
S_sp.MBTL60-112-B2_K5Q73_RS09050                 574  GTFTSATSAICSDCHLSD.......TAINHMTQQG.....GVFKGSKADATAGTESCA
S_sp.MBTL60_112_B1_K5Q83_RS07460                 618  SLYTTPIAATCMSCHVYSPESE..AKFTAHAEGQG....AKVNVPVAIADDAAQLETCF
S_sp.MBTL60_112_B1_K5Q83_RS07480                 574  GTFTSATSAICSDCHLSD.......TAINHMTQQG.....GVFKGSKADATAGTESCA
S_enrypsychrophilus_strain_YLB-08_FM038_RS07470  610  GTYTTPIAATCVSCHGYNMDS.....FKAHVEGQG....ALVNVTKEEATEAAQLETCF
S_enrypsychrophilus_strain_YLB-08_FM038_RS07490  578  GTFTSATAAICSDCHSS........ASNHMIQQG.....AVFMGTEEDAIAGTESCA
S_sp.YLB_09_FS418_RS09045                        610  GTYTTPIAATCVSCHGYNMDS.....FKAHVEGQG....ALVNVTKEEATEAAQLETCF
S_sp.YLB_09_FS418_RS09065                        578  GTFTSATAAICSDCHSS........ASNHMIQQG.....AVFMGTEEDAIAGTESCA
S_sp._WPAGA9_IGB07_RS05190                       598  GEYTTPITATCASCHGF..DE.....IKAHAEGQG....AVVNGSYDDANASAQLEFCM
S_sp._ARC9_LZ_GUY17_RS06940                      591  GLYTTPITATCASCHGF..DT.....ISQHAQSQG....AVVNGSYQEANDAAQLETCF
S_psychromarinicola_strain_M2_EGC80_RS12750      591  GLYTTPITATCASCHGF..DE.....IKQHVELQGG...AIVNGSYQEANDAAQLETCF
S_sp.Actino-trap-3_CXF80_RS00760                 591  GLYTTPITATCASCHGF..EA.....IQQHAENQG....AVVNGNYQEANDAAQLETCL
S_livingstonensis_strain_LMG_19866_EGC82_RS08000 591  GLYTTPITATCASCHGF..DT.....ISQHAQSQG....AVVNGSYQEANDAAQLETCF
Ferrimonas_lipolytica_strain_S7_HER31_RS03430    587  AQFSTPWAATCASCHGFDDEIT.....IEHMKLAGG...AVING..TEAEANQAIETCS
S_sp.ISTPL2_CCLCJOKE_1_HUB64_RS04610             588  GKYTTPITATCTSCHAP.......ESIGHGLENMG....AIVNGDYVQANQAAQSETCF
S_sp.ISTPL2_CCLCJOKE_1_HUB64_RS04620             575  GTFTSPIAAVCSNCHSDT.......STHNHMAQQG.....AVFAGTKSDATAGTETCA
S_japonica_strain_KCTC_22435_SJ2017_RS07855      598  GEYTTPITATCASCHGF..DE.....IKAHAEGQG....AVVNGSYDDANASAQLEFCM
S_sp.8A_M2897_RS07845                            588  GKYTTPITATCTSCHTIGTDKMT.KHSADTLVSFG....AVIDGDYTQANQAAQSETCF
S_sp.8A_M2897_RS07855                            576  GTFTSPIAAVCSNCHSSD.......TTHNHMMQQG.....AVFAGTKADATAGTETCA
S_woodyi_ATCC_51908_Swoo_3125                    604  G.YTTPIAATCYSCHSYSGDK.....FKNHVESQG....AKVNADYTVANEAAQLETCF
Ferrimonas_balerica_DSM_9799_Fbal_1357           575  ALYSTPRAATCISCHNPADTLAGGETLQQHIVNVGG...GLVDVPRDQADAGAQVETCF
Ferrimonas_balerica_DSM_9799_Fbal_1362           583  GTFTSLEAATCAVCHSSA.......SAQGHMTQQG.....AVFGG.DFDMAAGDKGCA
S_sediminis_HAW-EB3_Ssed_1525                    599  G.YTTPIAATCTSCHTS..DS.....TKLHAVGQG....AIIDGSYEAANDAAQLETCF
S_sediminis_HAW-EB3_Ssed_1528                    582  GTFTSATAAICSDCHFSD.......TAKNHMTQQG.....AVFMGSEADATAGTESCS
S_sp.KX20019_JK628_RS15030                       631  SLYTTPIAATCTSCH..TAES.....TMTHAAGQG....ALFNVDKDVANDAAQLETCF
Ferrimonas_sp.SCSIO_43195_J8Z22_RS15465          604  AGYGTPITATCASCHSYPSAID.....HMTGETFGN...GKFNLDKADAQLAVSAENCF
```

```
S_oneidensis_MR-1_mtrC                           657  YCHTP.TVADHTKVKM.....
S_oneidensis_MR-1_mtrF                           622  FCHGQGTVADVLKVHPIN...
S_sp._LZH-2_JM642_12750                          636  YCHTP.TVADHTQVKM.....
S_sp._LZH-2_JM642_12740                          622  FCHGQGAVADVLKVHPIN...
S_xiamenensis_NUITM-VS1_NUITMVS1_26680           636  YCHTP.TVADHTQVKM.....
S_xiamenensis_NUITM-VS1_NUITMVS1_26650           622  FCHGQGAVADVLKVHPIN...
S_sp._MR-4_Shewmr4_2510                          641  YCHTP.TVADHTQVKM.....
S_sp._MR-4_Shewmr4_2508                          622  FCHGQGAVADVLKVHPIK...
S_putrefaciens_strain_FDAARGOS_681_FOB89_16330   649  YCHKP.TPTDHTQVKM.....
S_fidelis_ATCC-BAA-318_L884_L884_RS0114715       656  YCHAP.TPGDHTSVKM.....
S_fidelis_ATCC-BAA-318_L884_L884_RS0114740       613  TCHGQGAAVDVLKVHPIQ...
S_piezotolerans_WP3_SWP_RS14705                  665  YCHAP.TPADHTAVKM.....
S_piezotolerans_WP3_SWP_RS14685                  620  TCHGQGASVDVLAVHPIK...
S_schlegeliana_strain_JCM_11561_JMA39_RS08985    657  FCHEP.TPNDHTAVKM.....
S_schlegeliana_strain_JCM_11561_JMA39_RS08965    624  TCHGQGATVDVLKVHPIQ...
S_marisflavi_strain_EP1_CFF01_RS06660            639  YCHAP.VIEDHTAVKM.....
S_marisflavi_strain_EP1_CFF01_RS06680            622  TCHRLGAEADVLKVHPIK...
S_sp.SUN_WT4__FJQ87_RS07980                      647  FCHKP.TVANHGHVEM.....
S_sp.MBTL60-112-B2_K5Q73_RS09070                 671  FCHKP.TADDHTSVKM.....
S_sp.MBTL60-112-B2_K5Q73_RS09050                 620  TCHGQGAAVDVLKVHPIK...
S_sp.MBTL60_112_B1_K5Q83_RS07460                 671  FCHKP.TADDHTSVKM.....
S_sp.MBTL60_112_B1_K5Q83_RS07480                 620  TCHGQGAAVDVLKVHPIK...
S_enrypsychrophilus_strain_YLB-08_FM038_RS07470  660  MCHNP.SINDHTSVKM.....
S_enrypsychrophilus_strain_YLB-08_FM038_RS07490  622  TCHGQGATADVLAVHPIK...
S_sp.YLB_09_FS418_RS09045                        660  MCHNP.SINDHTSVKM.....
S_sp.YLB_09_FS418_RS09065                        622  TCHGQGATADVLAVHPIK...
S_sp._WPAGA9_IGB07_RS05190                       646  GCHKP.TIFDHTAVNM.....
S_sp._ARC9_LZ_GUY17_RS06940                      639  FCHAP.TIENHTQVKL.....
S_psychromarinicola_strain_M2_EGC80_RS12750      640  YCHAP.TIENHTQVKL.....
S_sp.Actino-trap-3_CXF80_RS00760                 639  FCHAP.TIENHTQVKL.....
S_livingstonensis_strain_LMG_19866_EGC82_RS08000 639  FCHAP.TIENHTQVKL.....
Ferrimonas_lipolytica_strain_S7_HER31_RS03430    636  NCHAADALTEQHGVKF.....
S_sp.ISTPL2_CCLCJOKE_1_HUB64_RS04610             636  YCHKP.TPTDHTQVKM.....
S_sp.ISTPL2_CCLCJOKE_1_HUB64_RS04620             621  FCHGQGGVADVLKVHPIN...
S_japonica_strain_KCTC_22435_SJ2017_RS07855      646  GCHKP.TIFDHTAVNM.....
S_sp.8A_M2897_RS07845                            642  YCHTP.TVADHTQVKM.....
S_sp.8A_M2897_RS07855                            622  FCHGQGAVADVLKVHPIN...
S_woodyi_ATCC_51908_Swoo_3125                    653  MCHNP.SINDHTSVKM.....
Ferrimonas_balerica_DSM_9799_Fbal_1357           631  YCHKP.ELNNHTVVSF.....
Ferrimonas_balerica_DSM_9799_Fbal_1362           628  TCHGPGRSYDVAPAHGLAPAQ
S_sediminis_HAW-EB3_Ssed_1525                    646  FCHNP.AISDHTQVKM.....
S_sediminis_HAW-EB3_Ssed_1528                    628  TCHGEGAPADILKVHPINE..
S_sp.KX20019_JK628_RS15030                       679  YCHAP.TAEDHTAVKM.....
Ferrimonas_sp.SCSIO_43195_J8Z22_RS15465          655  VCHAP.DVADHSNVKF.....
```
